# NNT is a key regulator of adrenal redox homeostasis and steroidogenesis in male mice

**DOI:** 10.1530/JOE-16-0638

**Published:** 2017-10-18

**Authors:** E Meimaridou, M Goldsworthy, V Chortis, E Fragouli, P A Foster, W Arlt, R Cox, L A Metherell

**Affiliations:** 1 Centre for EndocrinologyWilliam Harvey Research Institute, John Vane Science Centre, Queen Mary, University of London, London, UK; 2 MRC Harwell InstituteGenetics of Type 2 Diabetes, Mammalian Genetics Unit, Oxfordshire, UK; 3 Institute of Metabolism and Systems ResearchUniversity of Birmingham, Birmingham, UK; 4 Centre for EndocrinologyDiabetes and Metabolism, Birmingham Health Partners, Birmingham, UK

**Keywords:** RNA sequencing, nicotinamide nucleotide transhydrogenase, redox homeostasis, steroidogenesis, ROS scavengers

## Abstract

Nicotinamide nucleotide transhydrogenase, NNT, is a ubiquitous protein of the inner mitochondrial membrane with a key role in mitochondrial redox balance. NNT produces high concentrations of NADPH for detoxification of reactive oxygen species by glutathione and thioredoxin pathways. In humans, NNT dysfunction leads to an adrenal-specific disorder, glucocorticoid deficiency. Certain substrains of C57BL/6 mice contain a spontaneously occurring inactivating *Nnt* mutation and display glucocorticoid deficiency along with glucose intolerance and reduced insulin secretion. To understand the underlying mechanism(s) behind the glucocorticoid deficiency, we performed comprehensive RNA-seq on adrenals from wild-type (C57BL/6N), mutant (C57BL/6J) and BAC transgenic mice overexpressing *Nnt* (C57BL/6J^BAC^). The following results were obtained. Our data suggest that *Nnt* deletion (or overexpression) reduces adrenal steroidogenic output by decreasing the expression of crucial, mitochondrial antioxidant (*Prdx3* and *Txnrd2*) and steroidogenic (*Cyp11a1*) enzymes. Pathway analysis also revealed upregulation of heat shock protein machinery and haemoglobins possibly in response to the oxidative stress initiated by NNT ablation. In conclusion, using transcriptomic profiling in adrenals from three mouse models, we showed that disturbances in adrenal redox homeostasis are mediated not only by under expression of NNT but also by its overexpression. Further, we demonstrated that both under expression or overexpression of NNT reduced corticosterone output implying a central role for it in the control of steroidogenesis. This is likely due to a reduction in the expression of a key steroidogenic enzyme, Cyp11a1, which mirrored the reduction in corticosterone output.

## Background

**Figure 1:**
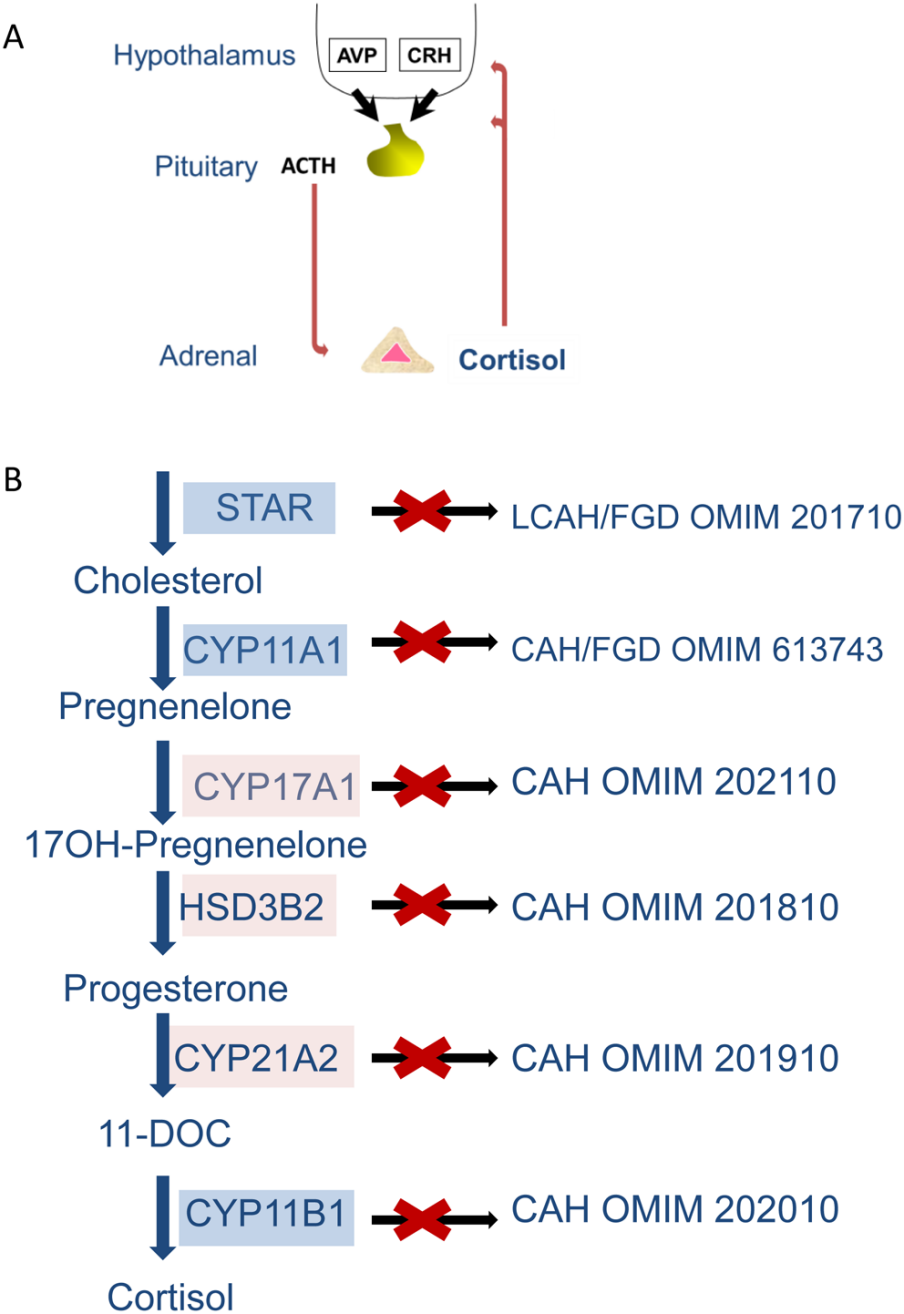
Hypothalamic–pituitary–adrenal (HPA) axis and enzymes responsible for cortisol production. (A) The HPA axis is a major component for adaptation of the stress response and cortisol release and consists of a complex set of feedback interactions that connect the central nervous and endocrine systems. In response to stress the paraventricular nucleus of the hypothalamus releases corticotropin-releasing hormone (CRH) and arginine vasopressin (AVP) that acts on the adrenal to stimulate glucocorticoid synthesis. (B) The conversion of cholesterol to cortisol is achieved by a series of catalytic reactions catalysed by mitochondrial (in blue) and microsomal (in pink) enzymes. Mutations in these key steroidogenic enzymes result in diseases of adrenal and gonadal insufficiency, indicated to the right with their reference numbers from online inheritance in man (OMIM). STAR, steroidogenic acute regulatory protein; CYP11A1, cytochrome P450 side chain cleavage enzyme; CYP17A1, 17-alpha hydroxylase; HSD3B2, 3-beta hydroxysteroid dehydrogenase, CYP21A2, 21-hydroxylase; LCAH, lipoid congenital adrenal hyperplasia; FGD, familial glucocorticoid deficiency; CAH, congenital adrenal hyperplasia.

**Figure 2:**
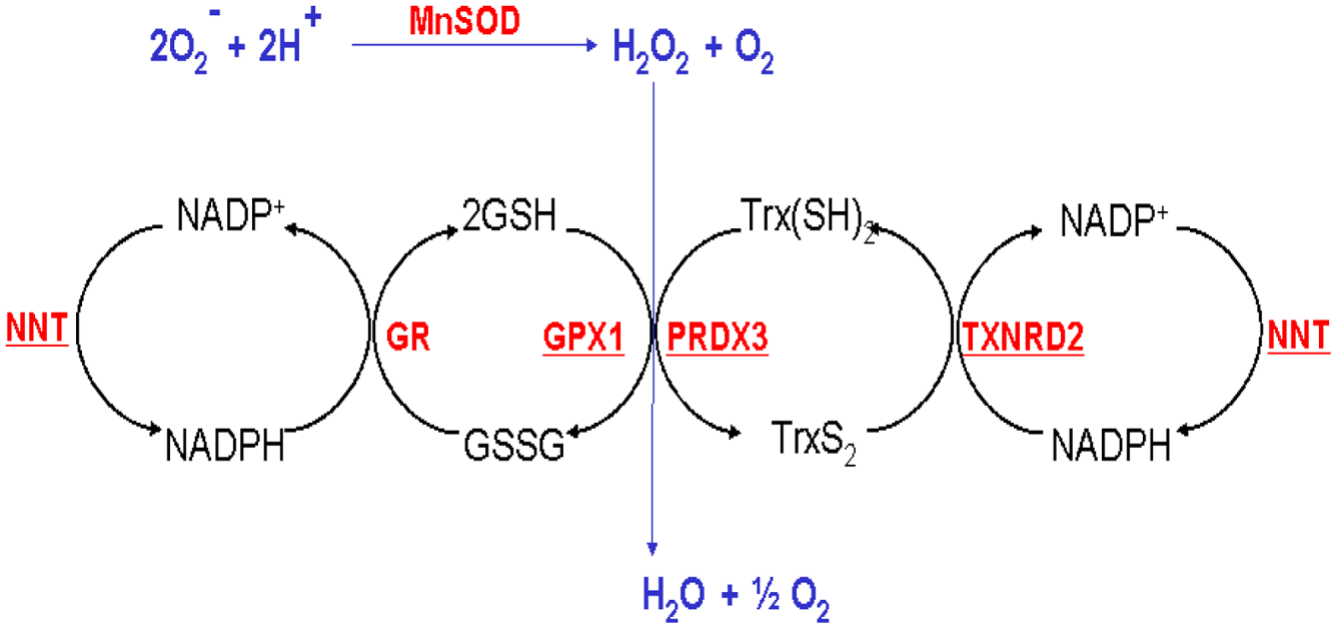
Detoxification of free radicals in the mitochondria. *NNT* encodes a protein, integral to the inner mitochondrial membrane, which under normal physiological conditions uses energy from the mitochondrial proton gradient to generate high concentrations of NADPH. This is required for many processes in the cell including the supply of reductive power to a network of antioxidant enzymes, specifically the glutathione (GSH/GSSG) and thioredoxin (Trx(SH)_2_/TrxS_2_) systems, to allow the detoxification of H_2_O_2_. Manganese superoxide dismutase (MnSOD) converts O_2·_^−^ into H_2_O_2_ and protects ROS-sensitive proteins from oxidative damage. H_2_O_2_ is then removed by glutathione peroxidases (e.g. GPX1) or peroxiredoxins (e.g. PRDX3) using GSH and Trx(SH)_2_ as co-factors. GSH and Trx(SH)_2_ can be regenerated by glutathione reductase (GR) and thioredoxin reductase-2 (TXNRD2), respectively, using the reducing power from NADPH. Without NNT, the production of NADPH is compromised, causing the mitochondria to become more sensitive to oxidative stress. Enzymes underlined in red are affected by one or more mutations in FGD patients.

Adrenal insufficiency is a rare, potentially fatal, endocrine disorder resulting from a failure of the adrenal cortex to respond to hormonal stimuli. In familial (or isolated) glucocorticoid deficiency, adrenal hormone output is preserved apart from a specific deficit of glucocorticoids. Normally, under the control of hypothalamic corticotropin-releasing hormone (CRH) and arginine vasopressin (AVP), the pituitary releases adrenocorticotropic hormone (ACTH), which acts on the adrenal via the ACTH receptor (otherwise known as *MC2R*) to produce glucocorticoids, mainly cortisol. This in turn acts on the hypothalamus and pituitary to suppress further production of ACTH in a negative feedback loop (Fig. 1A) (Keller-Wood & Dalman 1984). The human adult adrenal is characterised by three distinctive cortical zones surrounding the medulla; the zona glomerulosa (ZG) where mineralocorticoids are produced, the zona fasciculata (ZF), which synthesises glucocorticoids (mostly cortisol; in mice, the major glucocorticoid is corticosterone) and the zona reticularis (ZR) where androgen synthesis occurs (Vinson 2003). The first step of steroidogenesis occurs once cholesterol is transported from the outer to the inner mitochondrial membrane by the steroidogenic acute regulatory protein (STAR) and is converted to pregnenolone by the cholesterol side chain cleavage enzyme (CYP11A1). To make cortisol, pregnenolone then undergoes a series of intermediate reactions catalysed by microsomal enzymes (CYP17A1, HSD3B2 and CYP21A2) before the final step of cortisol production, catalysed by 11beta-hydroxylase (CYP11B1) or aldosterone production, catalysed by CYP11B2, both occurring in mitochondria (Fig. 1B) (Miller & Auchus 2011). The activities of steroidogenic cytochrome P450 (CYP) enzymes are reliant upon electron-donating redox partners; for mitochondrial (or type 1) enzymes, electrons are transferred from the reduced form of nicotinamide adenine dinucleotide phosphate (NADPH) by ferredoxin reductase (FDXR) and ferredoxin (FDX1), whereas the microsomal enzymes (Type 2) use P450 oxidoreductase (POR) as their redox partner. Hence, the first and last steps of cortisol production occur in the mitochondria and require a constant supply of reductant NADPH. This NADPH is regenerated from NADP by a few pathways including the thioredoxin and glutathione pathways, which are ultimately enabled by NNT (Fig. 2).

Perturbations in this pathway cause a number of steroidogenic defects affecting adrenal and gonadal steroidogenesis. *STAR* mutations give rise to lipoid congenital adrenal hyperplasia (OMIM 201710), a severe syndrome of adrenal and gonadal insufficiency resulting in XY sex reversal; *CYP11A1* defects give a similar clinical picture but without the lipid build up in steroidogenic tissues seen with *STAR* mutations. *HSD3B2*, *CYP17A1*, *CYP21A2*, *CYP11B1* and *POR* mutations give rise to four variants of congenital adrenal hyperplasia (OMIM 201810, 202110, 201910, 202010, and 201750 respectively) and *CYP11B2* mutations give rise to hypoaldosteronism (OMIM 203400) (Fig. 1B) (Miller & Auchus 2011). No mutations have yet been described in humans in *FDXR*/*FDX1* – perhaps due to embryonic lethality. Partial loss-of-function changes in *STAR* and *CYP11A1* can present with a less severe phenotype akin to our disease of interest, familial or isolated glucocorticoid deficiency (FGD) (Baker *et al*. 2006, Metherell *et al*. 2009, Rubtsov *et al*. 2009, Sahakitrungruang *et al*. 2010, 2011, Parajes *et al*. 2011). In FGD, the two most common gene defects are mutations in the melanocortin 2 receptor and its accessory protein (*MC2R* and *MRAP*), but recently, we have described defects in NADPH supply to and/or antioxidant defence in mitochondria, with defects in two genes, *NNT* and thioredoxin reductase 2 (*TXNRD2*), giving disorders of adrenal insufficiency primarily compromising glucocorticoid production (Meimaridou *et al*. 2012, 2013, Prasad *et al*. 2014). *MC2R* and *MRAP* are adrenal zone and ACTH pathway specific, so it is unsurprising that they give rise to isolated glucocorticoid deficiency, whereas *NNT* and *TXNRD2* are ubiquitously expressed.

NNT is the major mitochondrial enzymatic source of NADPH contributing 45% of the total NADPH supply (Nickel *et al*. 2015). It exists as a dimer and spans the inner mitochondrial membrane modulating H^+^ movement and supplying the high concentrations of NADPH required for the detoxification of ROS by glutathione and thioredoxin pathways (Fig. 2). Even though the gene is ubiquitously expressed, the organ specific physiological roles of NNT are only gradually being revealed by the study of a C57BL/6J mouse substrain that has a spontaneous mutation in *Nnt* (an in-frame 5-exon deletion), resulting in the truncation of the message and absence of the protein (Nickel *et al*. 2015). The first consequence of this murine *Nnt* deletion, described by Toye and coworkers in 2005, was glucose intolerance and reduced insulin secretion (Toye *et al*. 2005). Subsequent to the finding of human mutations causing FGD, we showed that 3-month-old mice had 50% lower basal and stimulated levels of corticosterone than their wild-type counterparts. Histological examination of their adrenals revealed a slightly disorganized ZF with higher levels of apoptosis than the wild-type C57BL/6N strain (Meimaridou *et al*. 2012). More recently, it was reported that liver mitochondria from C57BL/6J mice have major redox impairments resulting in an inability to maintain NADP and glutathione in their reduced states (Ronchi *et al*. 2013).

Previously, we have shown that H295R cells where *Nnt* has been stably knocked down undergo oxidative stress as demonstrated by low glutathione levels, and increased mitochondrial superoxide production (Meimaridou *et al*. 2012). Similar defects in energy metabolism due to *Nnt* ablation have also been demonstrated in other mouse tissues (heart, liver, pancreas) emphasising the importance of NNT for cellular bioenergetics (Sauer *et al*. 2004, Ronchi *et al*. 2013, Nickel *et al*. 2015). However, the mechanism by which loss of Nnt causes the adrenal-specific pathology we observe is unclear.

Here, we aim to investigate the effect of NNT loss and overexpression in the adrenal cortex by performing RNA-seq on adrenals from mice, which are wild-type (C57BL/6N, *Nnt*^+/+^), null (C57BL/6J, *Nnt*^−/−^) or 2-fold overexpressors (BAC transgenic, *Nnt*^BAC^) of *Nnt* (Freeman *et al*. 2006).

## Materials and methods

### Mouse strains

All mice were bred, housed and culled at MRC Harwell and therefore the husbandry was identical for all 3 substrains. The mouse strains used were C57BL/6NHsd originally from Harlan (Harlan Laboratories UK), which is wild type for *Nnt* (*Nnt^+/+^*), C57BL/6J originally from Charles River (Charles River UK), which has an in-frame deletion of 5 *Nnt* exons (*Nnt^−^^/^^−^*) and C57BL/6J mice carrying a BAC transgene to restore murine *Nnt* (*Nnt^BAC^*; Freeman *et al*. 2006), which we show are 2-fold overexpressors. For RNA-seq, 18-month-old male mice of the three different substrains were utilised, 5 mice per group. Mice were culled with an overdose of Euthatal (to allow for the collection of blood) and tissues were then removed quickly and either fixed or flash frozen in liquid nitrogen. All mice were culled between 10:00 and 11:30 h and adrenals were removed. The animal protocols used in this study were approved by United Kingdom Home Office.

### Genotyping

Genomic DNA was extracted from the mouse tail tissue using a Qiagen DNeasy tissue kit. Mice were genotyped for *Nnt* status using previously published primers (Huang *et al*. 2006).

### Mouse histology

Mouse adrenals from *Nnt^+/+^*, *Nnt^−^^/^^−^* and *Nnt^BAC^* were fixed in 4% paraformaldehyde (Sigma) and embedded in paraffin. Sections were obtained using a microtome (Microm HM 325, Thermo Fisher) at 6-μm thickness, and hematoxylin & eosin (H&E) staining was performed using standard procedures (Guasti *et al*. 2011).

To assess changes in lipid content between the three mice strains, we performed Oil Red O staining as described previously (www.ihcworld.com/_protocols/special_stains/oil_red_o.htm). Briefly sections of fresh frozen adrenal tissues were obtained at 5 µm thickness and fixed in ice cold 10% formalin for 5 min. Sections were air dried and placed in absolute propylene glycol for 5 min to avoid carrying water into Oil Red O. Sections were then stained with pre-warmed Oil Red O solution for 8–10 min at 60°C and then washed twice with distilled water. Images were acquired using a Leica DMR microscope (Leica), and digital images were captured using a Leica DC200 camera (Leica) and DCViewer software (Leica).

### Steroid profile

Serum steroids were quantified using liquid chromatography–tandem mass spectrometry (LC–MS/MS) as previously described (O’Reilly *et al*. 2014). Steroids were extracted from 200 µL of serum (after addition of internal standard) using 1 mL tert-butyl methyl ether (MTBE). After freezing at −20°C for 1 h, the MTBE layer was transferred into a 96-well plate and evaporated under nitrogen at 55°C. Samples were reconstituted in 125 µL of a 50:50 solution of methanol (Sigma) and H_2_O (Sigma). Steroids were analysed on a Waters Xevo with Acquity uPLC, steroids were eluted from a HSS T3 1.8 µm, 1.2 × 50 mm column with a methanol/water 0.1% formic acid gradient system. Two mass transitions were used to identify and quantify each steroid (corticosterone: 347.2 > 121.2 and 347.2 > 97; deoxycorticosterone: 331 > 97 and 331 > 109.

### Generation of stable NNT knockdown (KD) and scrambled (SCR) H295R cell lines

Lentiviral plasmids (RHS4430-98851990; RHS4430-98913600; RHS4430-98524425; RHS4430-101033169 RHS4430-101025114) were obtained from OpenBiosystems in a p.GIPZ backbone and contained shRNA specific for human NNT (NM 012343) under the control of the CMV promoter, plus the puromycin resistance and green fluorescence protein (GFP) genes. HEK293T cells (packaging cells) were transiently transfected with the shRNA plasmids, two days after transfection virus containing media was collected, filtered using a 0.22 µm filter and used to transduce H295R cells. Four days after infection GFP-positive cells were selected in 4 µg/mL puromycin. Transduction efficiency was determined by fluorescence microscopy. A scrambled (control) cell line was generated in a similar fashion using a non-specific shRNA.

### NADP/NADPH assay

To measure total and reduced nicotinamide adenine dinucleotide phosphates (NADP+ and NADPH respectively) stably transfected H295R cells were plated onto white-walled and white bottomed 96-well culture dishes (Corning Costar). After 24 h, NADP+ and NADPH were measured using NADP/NADPH-Glo assay (Promega), a luminescence-based system and according to the manufacturer’s protocol. Luminescence was recorded after 15 min using Omega Luminometer (BMGLabTech) and with integration time of 0.5 s.

### Oxygen consumption rate-XF extracellular flux analyser

Scrambled (SCR) and stable knockdown H295R cells (NNT-KD) were cultured on Seahorse XF-96 microplates and allowed to grow overnight. On the day of metabolic flux analysis, cells were changed to unbuffered DMEM (DMEM base medium supplemented with 10 mM glucose, 1 mM sodium pyruvate, 2 mM l-glutamine, pH 7.4) and incubated at 37°C in a non-CO_2_ incubator for 1 h. All medium and injection reagents were adjusted to pH 7.4 on the day of assay. Baseline measurements of oxygen consumption rate (OCAR, measured by oxygen concentration change) and extracellular acidification rate (ECAR, measured by pH change) were taken before sequential injection of treatments/inhibitors: oligomycin (ATP synthase inhibitor, 4 µM), FCCP (mitochondrial respiration uncoupler, 1 µM) and rotenone (Complex I inhibitor, 1 µM).

### RNA-seq

RNA from mouse adrenal tissues (*Nnt^+/+^*, *Nnt^−^^/^^−^*, *Nnt^BAC^*) was extracted using the RNeasy Mini kit (Qiagen). Once RNA quality and concentration were tested, samples were processed by Oxford Gene Technology (www.ogt.co.uk). Enrichment and library preparation were performed using Illumina TruSeq RNA Sample Prep Kit, v2; total RNA was captured with oligo-dT-coated magnetic beads. The mRNA was fragmented and then first-strand cDNA synthesis was initiated from random primers, followed by second-strand synthesis. After end-repair, phosphorylation and A-tailing, adapter ligation and PCR amplification were performed to prepare the library for sequencing. Paired-end sequencing was performed over 100 cycles on the Illumina HiSeq2000 platform using TruSeq v3 chemistry. Fastq files were generated from the sequencing platform via the manufacturer’s proprietary software. For mapping and alignment, reads were processed through Tuxedo suite (Trapnell *et al*. 2013). Reads were mapped to their location on the appropriate Illumina iGenomes build using Bowtie, and splice junctions were identified using Tophat. Cufflinks was used to perform transcript assembly, abundance estimation and differential expression and regulation for the samples. RNA-Seq alignment metrics were generated using Picard.

### Lipid peroxidation assay

Lipid peroxidation in mouse adrenal lysates was assessed by using a lipid peroxidation assay kit (Abcam) based on the detection of malondialdehyde (MDA) in the samples. Adrenal tissues from *Nnt*^+/+^, *Nnt*^−/−^ and *Nnt*^BAC^ mice were excised and homogenised in MDA lysis buffer provided in the kit. Lysates were then centrifuged at 13,000 ***g*** for 10 min and the supernatant was collected for lipid peroxidation measurements. Samples were incubated with thiobarbituric acid (TBA), which interacts with MDA present in the samples to generate MDA-TBA adducts. These adducts were quantified colorimetrically at 532 nm.

### Immunoblotting analysis

Immunoblotting was used to assess protein expression. Cells were lysed in RIPA buffer containing protease and phosphatase inhibitors (SIGMA) and then left on ice for 30 min. Samples were centrifuged for 15 min at 17,000 ***g***. Supernatant was collected and an equal volume of Laemmli buffer was added. Samples were heated at 95–100°C for 5 min and then loaded on 4–12% SDS gels. Protein separation was performed by using the Invitrogen electrophoresis system. Proteins were then transferred to nitrocellulose membrane (Sigma Aldrich) using semi-dry transfer blot (Biorad) at 15 V for 1 h. Membranes were probed with one of mouse anti-Nnt (1:1000; SIGMA, HPA004829), mouse anti-actin (1:5000; SIGMA, A5441), rabbit anti-TXNRD2 (1:1000;SIGMA, SAB2702064), rabbit anti-PRDX3 (1:500; ProteinTech, 55087-1-AP), rabbit anti-GPX1 (1:500; Abcam ab108429), mouse anti-STAR (1:1000; Abcam, ab58013), rabbit anti-CYP11A1 (1:1000; Cell Signalling, 14217), rabbit anti-HSD3B2 (1:500; Aviva Biosystems, OAGA02009) or rabbit anti-CYP21A2 (1:500; SIGMA HPA053371). Visualisation of the proteins was performed by using Alexa-Fluor 680 and 800 secondary antibodies (1:5000; Invitrogen) and the Li-CoR Odyssey system.

### Statistics

Statistical analyses were performed using a combination of one-way ANOVA using *Tukey HSD* (honest significant difference) test and a two-tailed Student’s *t*-tests assuming unequal variance in 5 mice per group. All values were expressed as a mean ± s.e.m., and *P* values <0.05 were considered significant.

## Results

### Mouse phenotyping

We have previously reported that 3-month-old mice carrying a spontaneous *Nnt* 5-exon deletion (C57BL/6J, *Nnt*^−/−^) have significantly lower levels of corticosterone (50%) than their wild-type counterparts (C57BL/6N, *Nnt*^+/+^) (Meimaridou *et al*. 2012). To identify the mechanism by which loss of NNT affects steroidogenesis we employed the same mouse models but also included an additional mouse line where the *Nnt* loss had been rescued in C57BL/6J mice by transgenic expression of the entire murine *Nnt* gene contained within a bacterial artificial chromosome (BAC transgenic, *Nnt*^BAC^) (Freeman *et al*. 2006).

### Glucocorticoid levels in the three mouse strains

**Figure 3:**
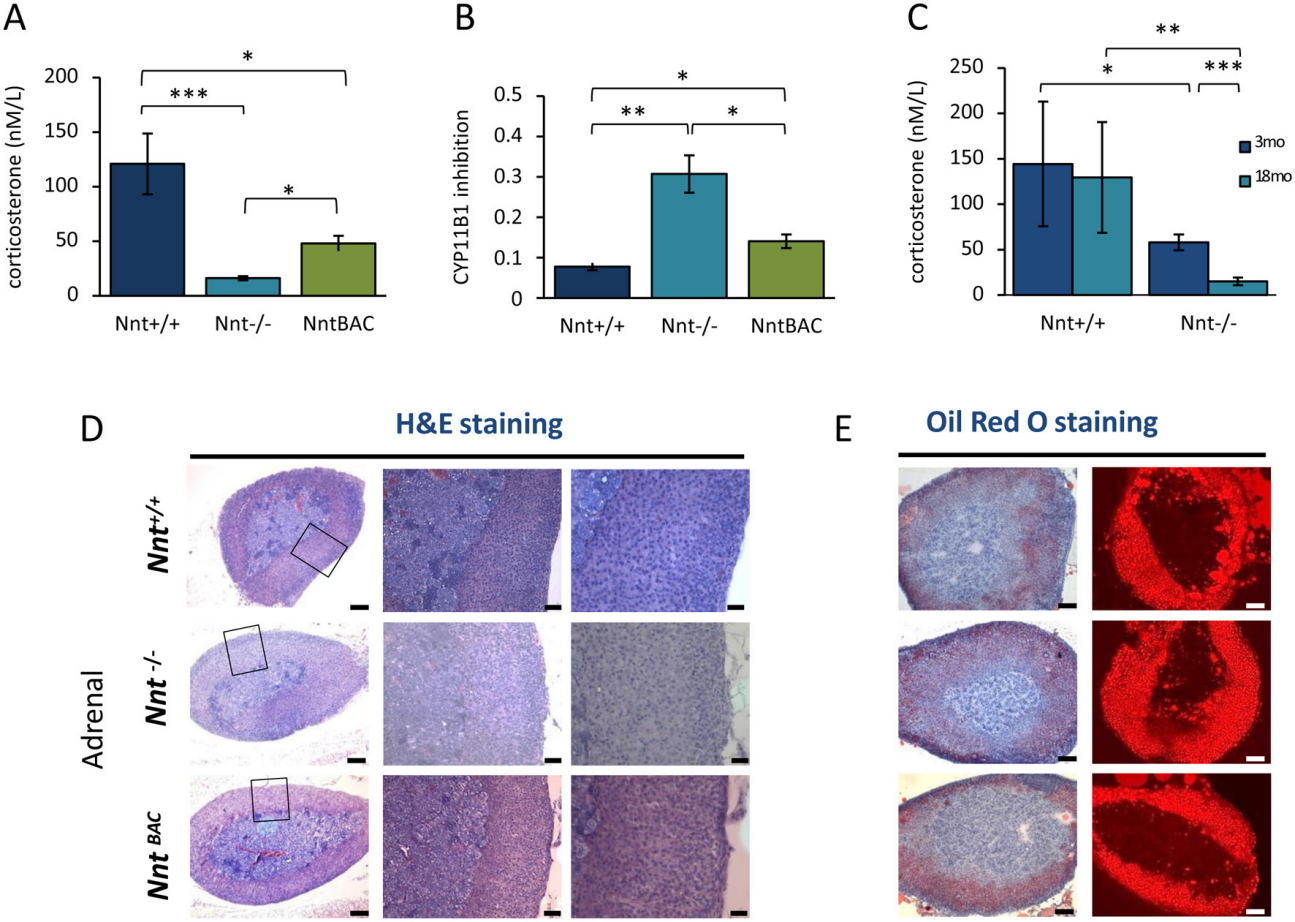
Biochemical and phenotypic characterisation of *Nnt^+/+^*, *Nnt^−^^/^^−^* and *Nnt^BAC^* mice. (A) Serum corticosterone in *Nnt^+/+^*, *Nnt^−^^/^^−^* and *Nnt^BAC^* mice was measured by LC–MS/MS and showed 80% and 50% reduction in *Nnt^−^^/^^−^* and *Nnt^BAC^* mice, respectively. (B) The 11-deoxycorticosterone (DOC)/corticosterone ratio (CYP11B1 inhibition) was significantly higher in *Nnt^−^^/^^−^* and *Nnt^BAC^* than *Nnt^+/+^* mice. (C) Corticosterone synthesis deteriorates in 18 months *Nnt^−^^/^^−^* mice whereas there is no significant difference in the levels between 3 and 18 month *Nnt*^+/+^ mice. (D) H&E staining of mouse adrenals showed no major histological differences in architecture or zonation (left panel) and (E) Oil Red O staining revealed no difference in lipid content of the adrenals among the three mouse strains (right panels). Results are means ± standard deviation (s.d.); *n* = 5 mice per group, *P < 0.05, **P < 0.01, ***P < 0.001.

The adrenal steroid output of the three mouse strains was measured at 18 months in murine serum samples employing liquid chromatography–tandem mass spectrometry (LC–MS/MS). Changes in steroid production were observed predominantly for corticosterone, with reduction to 14% of wild-type levels in *Nnt*^−/−^ mice (Fig. 3A). Corticosterone levels in the *Nnt*^BAC^ mice restored serum corticosterone in part, but only to 40% of wild-type (*Nnt^+/+^*) levels, suggesting that overexpression of *Nnt* also perturbs steroidogenesis. 11-Deoxycorticosterone levels were not significantly different among the mice and therefore the ratio of 11-deoxycorticosterone/corticosterone was significantly higher in *Nnt*^−/−^ and *Nnt*^BAC^ mice indicating lower enzyme activity of *Cyp11b1* (Fig. 3B). We also noted that, over time, the deficit in glucocorticoid output worsened for the *Nnt*^−/−^ mice, between 3 and 18 months corticosterone levels in *Nnt*^+/+^ were unaltered, whereas there is a 60% decrease for *Nnt*^−/−^ mice, suggesting progressive loss of function (Fig. 3C).

### No changes to adrenal histology

In humans with FGD, there is a specific loss of glucocorticoid output from the ZF and relative preservation of steroid output from the other zones suggesting loss of this specific zone. Consistent with these findings, *Mc2r*^−/−^ mice have smaller adrenals with preservation of ZG and medulla but atrophied ZF (Chida *et al*. 2007). Interestingly all *Mc2r*^−/−^ mice on a pure C57BL/6J background die within two days of birth whereas those on a mixed B6/Balbc have much higher survival rates, with half making it to adulthood perhaps due to the restoration of *Nnt* levels (Chida *et al*. 2009), making *Nnt* a genetic modifier of their adrenal phenotype akin to the situation in another mouse model where NNT has a protective role in superoxide dismutase deficient mice (Huang *et al*. 2006). To determine whether loss of ZF occurs in Nnt^−/−^ mice we performed H&E staining in adrenal sections from the three mouse substrains. This showed no morphological differences among the strains in adrenal zonation (Fig. 3D). This was supported by the finding that genes differentially expressed between zones were not altered (see below). This suggested no major remodelling of the adrenals due to NNT loss and gave us confidence that the RNA-seq variations were not due to changes in zonation. Furthermore, on Oil Red O staining, we observed no differences in lipid levels between the mouse strains suggesting there is neither a dearth of cholesterol supply for steroidogenesis nor a surplus due to a cholesterol transport defect as seen with *Star* mutations (Sasaki *et al*. 2008) (Fig. 3E).

### *Nnt* deletion causes oxidative stress in mouse adrenals

**Figure 4:**
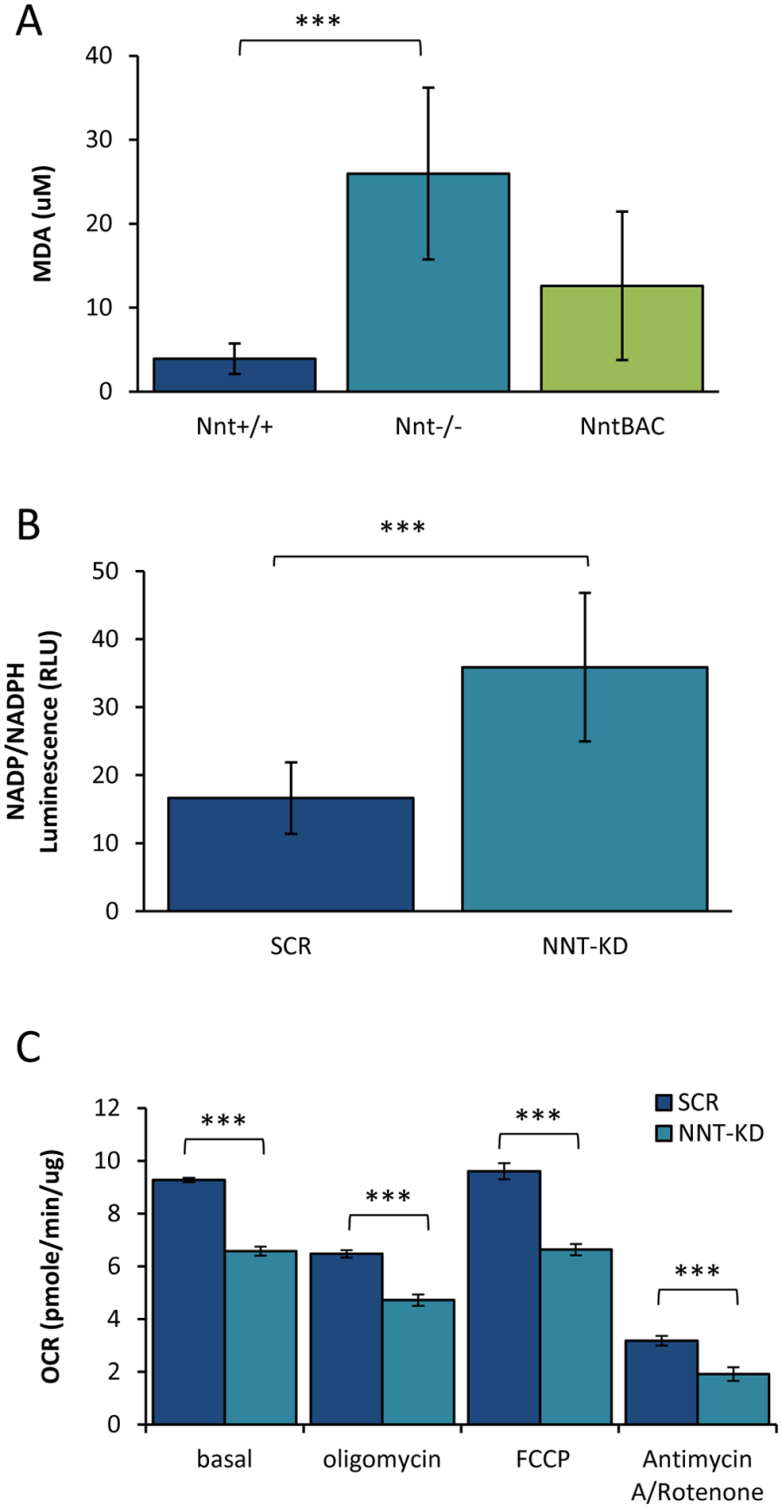
Oxidative stress on NNT ablation. (A) Lipid peroxidation represented by MDA levels were measured in adrenals of *Nnt^+/+^*, *Nnt^−^^/^^−^* and *Nnt^BAC^* mice. MDA levels were significantly increased in the adrenals of Nnt^−/−^ mice with a partial rescue in Nnt^BAC^ mice indicating lipid damage by free radicals in adrenals upon *Nnt* deletion (B) The cellular levels of NADP/NADPH in H295R cells with a stable knockdown of NNT (NNT-KD) were measured by using a luciferin based assay. Total cellular NADP/NADPH ratio was significantly higher in NNT-KD compared to scramble (SCR), suggesting that NNT is required to maintain the redox state of the intracellular NADPH and NADP+ pools. (C) Mitochondria respiration was assessed by measuring oxygen consumption rates using Seahorse XF-96 metabolic Flux Analyser. NNT-KD cells had a significantly lower basal OCAR compared to scrambled cells; the addition of oligomycin (complex V inhibitor) resulted in an OCAR decline which was significantly lower in NNT-KD cells compared to controls, indicating that the ATP turnover was compromised in NNT-KD cells. Furthermore, maximal respiration capacity as measured by the addition of an uncoupling agent, FCCP, was also significantly lower in NNT-KD cells. The addition of rotenone and antimycin (complex I and II inhibitors respectively) reflecting the spare respiratory capacity of the cells resulted in a reduction of OCAR which was significantly lowered in NNT-KD cells when compared to scrambled cells. OCAR values were normalised to total protein concentration. Results are means ± standard deviation (s.d.); *n* = 5 per group, *P < 0.05, **P < 0.01, ***P < 0.001.

We measured lipid peroxidation (LPO) by a malondialdehyde (MDA) assay as a measure of oxidative stress in these mice (5 mice per group). There was a significant increase in LPO in the adrenals of Nnt^−/−^ mice which returned towards wild-type levels in Nnt^BAC^ mice indicating lipid damage by free radicals in adrenals upon *Nnt* deletion (Fig. 4A). We saw the same trend in H_2_O_2_ levels (increased in Nnt^−/−^ and reduced again in Nnt^BAC^) (data not shown).

Impaired redox homeostasis caused by lack of *NNT* was further supported by *in vitro* studies in human adrenocortical H295R cells. The total cellular NADP/NADPH ratio was significantly higher in H295R with lentiviral knockdown of *NNT* (NNT-KD) compared to scrambled control cells (SCR), suggesting that NNT is required to maintain the redox state of the intracellular NADPH and NADP+ pools (Fig. 4B).

The perturbation in NADP/NADPH balance affected mitochondrial respiration resulting in significantly lower oxygen consumption rates (OCAR) in cells where NNT is knocked down (Fig. 4C).

### Transcriptome profiling by RNA-seq in adrenals from the C57BL/6 mouse substrains

**Figure 5:**
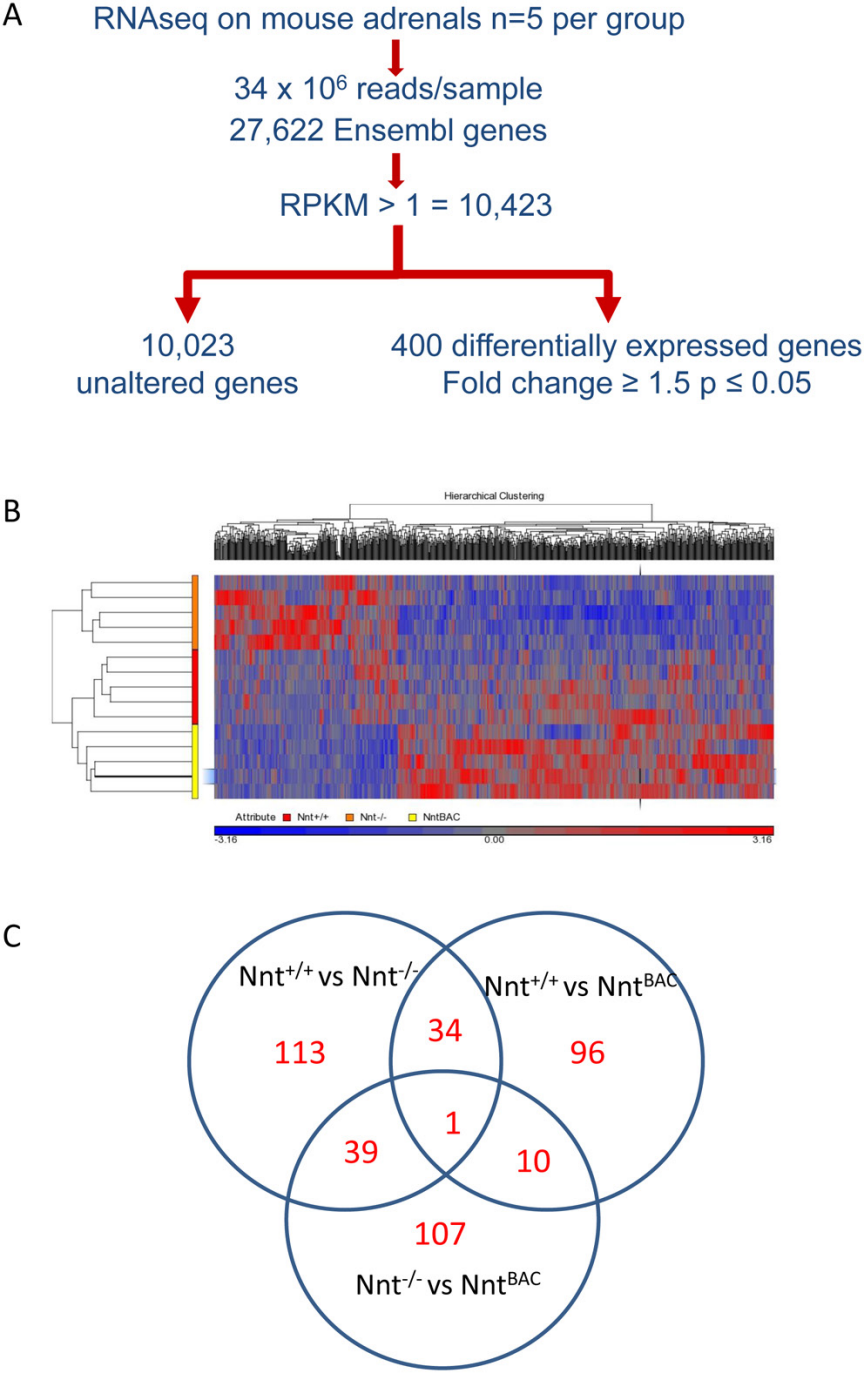
RNA-seq analysis flowchart and differential gene expression. (A) Flowchart of initial RNA-seq analysis of mouse adrenals. (B) Representative heat map of RNA-seq analysis for substrain-specific differentially expressed genes (between *Nnt*^−/−^ and *Nnt^BAC^*) within mouse adrenals. Genes were clustered by Partek hierarchical clustering based on gene expression values. Normalisation was performed by genes shifted to mean of zero and scaled to s.d. of 1. Arbitrary signal intensity from RNA-seq data is represented by colours (red, higher expression, blue lower expression). (C) Venn diagram showing the number of differential genes in pairwise analyses between; *Nnt^+/+^* vs *Nnt*^−/−^ (187), *Nnt*^−/−^ vs *Nnt^BAC^* (157) and *Nnt^+/+^* vs *Nnt^BAC^* (141). Genes at the intersection of the pairwise analyses *Nnt^+/+^* vs *Nnt*^−/−^ and *Nnt*^−/−^ vs *Nnt^BAC^* represent genes that are modulated by *Nnt* levels (39 + *Nnt*) (Table 1 and Supplementary Tables 3, 4, 5, 6, 7 and 8).

To investigate the cause of the reduced steroid production upon *Nnt* deletion, RNA was extracted from *Nnt^+/+^*, *Nnt*^−/−^, and *Nnt*^BAC^ mouse adrenals and the transcriptome profiled by RNA-seq. Using an Illumina HiSeq 2000 sequencer we obtained an average of 30 million reads per sample, with ~98% of these reads mapped to the mouse reference genome. In total, 27,622 genes were analysed in *Nnt^+/+^*, *Nnt*^−/−^ and *Nnt*^BAC^ adrenals (Fig. 5A and Supplementary Table 1, see section on supplementary data given at the end of this article). The most highly expressed categories of genes were mitochondrially encoded electron transport chain genes (*mt-Co1, mt-Co2, mt-Atp6, mt-Co3, mt-Cytb, mt-Nd4, mt-Nd2, mt-Nd1, mt-Atp8, mt-Nd6, mt-Nd5, mt-Nd4l, mt-Nd3, mt-Rnr1*), followed by steroid pathway genes *Star, Cyp21a1* and *Hsd3b1*, which are high up in the steroidogenic cascade (Supplementary Table 1) perhaps reflecting the high mitochondrial content of and high demand for steroidogenesis in the adrenal.

Initial data analysis was performed between paired samples comparing *Nnt^+/+^* and *Nnt*^−/−^, *Nnt^+/+^* and *Nnt*^BAC^ and *Nnt*^−/−^ and *Nnt*^BAC^ adrenals. To identify differentially expressed genes from each group we used the following criteria; (1) gene expression level greater than or equal to 1 read per kilobase of exon per million fragments mapped (RPKM) in all samples; (2) change in expression level greater than or equal to 1.5-fold; and (3) significance *P* value <0.05. This revealed differential expression (fold change ≥1.5; *P* < 0.05) of 400 genes in total in the pairwise comparisons (Fig. 5B, C and Supplementary Table 2). Only 1 gene varied between all three pairwise analyses and that was *Nnt* itself (see below and Fig. 5C). We hypothesized that genes that were up- or downregulated in *Nnt^−^^/^^−^* and their levels restored in *Nnt^BAC^* would be genes that were modulated by NNT.

### NNT levels in the three mouse strains

**Figure 6:**
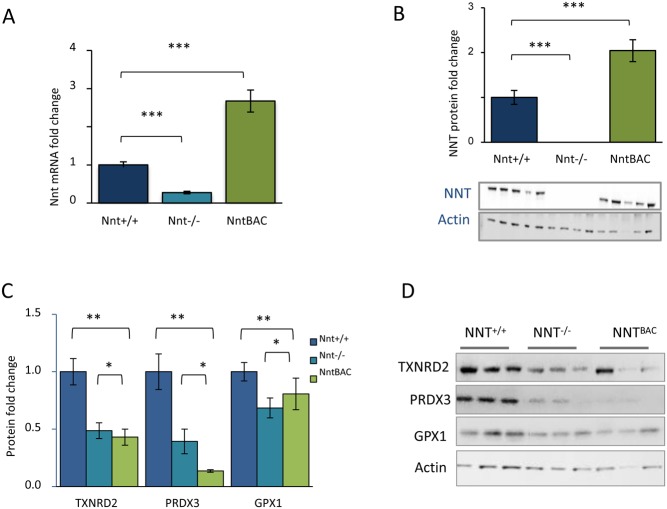
Effect of NNT loss on redox homeostasis. (A) mRNA *Nnt* levels in the three mouse strains. (B) No NNT protein expression was observed in *Nnt^−^^/^^−^* (Western blot) however there was a two-fold upregulation in *Nnt^BAC^* when compared to *Nnt^+/+^*. (C) Protein levels of TXNRD2, PRDX3 and GPX1 in the *Nnt^+/+^*, *Nnt^−^^/^^−^* and *Nnt^BAC^* mouse adrenals were normalised to actin with representative Western blots shown to the right.

*Nnt* expression levels in the three mouse strains were determined and while we observed very low mRNA levels in *Nnt^−^^/^^−^* mice (3.7-fold downregulation *P* = 0.012), there was a 2.7-fold (*P* = 1.8 × 10^−5^) increase in *Nnt* expression in *Nnt*^BAC^ mice over that for *Nnt^+/+^* (Fig. 6A). To investigate whether mRNA levels corresponded to protein expression we performed a Western blot on adrenal lysates from the same mice. NNT was undetectable in the *Nnt^−^^/^^−^* mice whereas a two-fold increase in NNT was observed in the adrenals of *Nnt*^BAC^ mice, in keeping with the RNA-seq data (Fig. 6B). These results suggest that the *Nnt*^BAC^ represents a modest *Nnt* overexpressor.

### No differential expression of other genes with polymorphisms identified between substrains

Recently, comparative genomics between C57BL/6J and C57BL/6N strains has identified many SNPs and structural variants that may contribute to the phenotypic differences between the two strains (Simon *et al*. 2013). To check whether these SNPs altered expression of these genes in the adrenal we specifically looked at their mRNA expression levels. Except for *Cilp*, which was up in Nnt^BAC^ (1.6 fold over Nnt^+/+^ (*P* 0.013)) but unaltered between *Nnt^+/+^* and *Nnt^−^^/^^−^*, we found no alterations in expression levels of the genes with interstrain variations, suggesting that the effects we describe are largely due to differential *Nnt* levels.

### No differential expression of zone specific genes between substrains

Many genes show differential expression between the zones of the adrenal, classically for example tyrosine hydroxylase (*Th*), aldosterone synthase (*Cyp11b2*), 3-beta-hydroxysteroid dehydrogenase (*Hsd3b2*), cytochrome b5 (*Cyb5*) and aldo-keto reductase family 1 member C3 (*Akr1c3*). More recently transcriptomic analyses have identified hundreds of other genes with differential expression between ZF and ZG (Nishimoto *et al*. 2012, Rege *et al*. 2014). None of these genes showed differential expression in our study suggesting no major remodelling of adrenals glands in Nnt^−/−^ or Nnt^BAC^ mice.

### *Nnt* deletion does not alter the levels of ACTH receptor pathway genes or other genes associated with adrenal insufficiency

*Mc2r* expression was unaltered between *Nnt^+/+^* vs *Nnt^−^^/^^−^* but up in *Nnt^BAC^* vs *Nnt^−^^/^^−^* (1.6-fold *P* = 0.023) whereas *Mrap* levels were the same across the 3 groups (data not shown). No significant expression level changes were observed at RNA level for other genes causing adrenal insufficiencies in humans (*Aaas*, *Abcd1*, *Aire*, *Cdkn1c*, *Cyp11a1*, *Cyp11b1*, *Cyp17a1*, *Cyp21a1*, *Mcm4*, *Nr5a1*, *Por* and *Txnrd2*).

### *Nnt* deletion alters antioxidant gene levels

Nnt provides a constant supply of NADPH required for ROS detoxification by the thioredoxin and glutathione systems. Malic enzyme 3 (*Me3*) and isocitrate dehydrogenase 2 (*Idh2*) are alternate NADPH suppliers although NNT is the major contributor (14). To investigate whether *Nnt* deletion leads to perturbation of other antioxidant enzymes perhaps to compensate for its loss, we looked at the mRNA and protein levels of antioxidant enzymes in these pathways. There was a modest reduction in gene expression levels of the antioxidants *Prdx3* and *Txnrd2* which did not reach statistical significance, however at the protein level they were significantly reduced in *Nnt*^−/−^ mice vs *Nnt*^+/+^ and the levels remained significantly low in *Nnt*^BAC^ when compared to *Nnt*^+/+^ (Fig. 6C and D). This is likely because *Nnt*^BAC^ overexpress NNT which may also cause redox imbalance, implying fine tuning of NNT is required for redox homeostasis. We observed no compensatory increase in the alternative NADPH suppliers *Me3* or *Idh2*.

### *Nnt* deletion alters mitochondrial cytochrome P450scc levels

**Figure 7:**
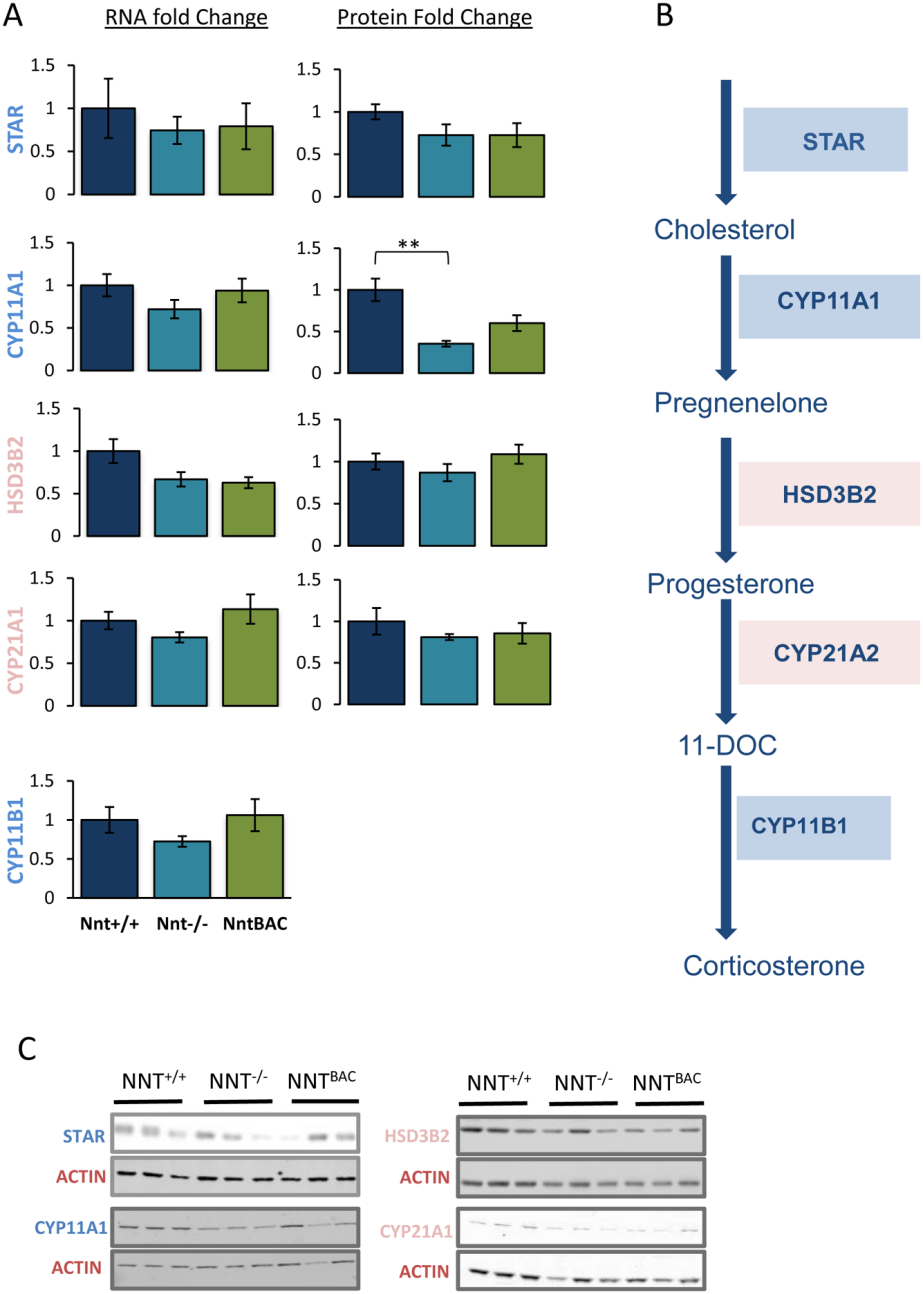
Effect of NNT loss on steroidogenesis. (A) mRNA and protein fold change of enzymes involved in glucocorticoid synthesis, starting from cholesterol transport (STAR), to the first step of steroid synthesis (mitochondrial CYP11A1), and subsequent reactions catalysed by microsomal enzymes (HSD3B2, CYP21A1) to the final synthesis of corticosterone. (B) The conversion of cholesterol to corticosterone in mice is achieved by a series of catalytic reactions catalysed by mitochondrial (in blue) and microsomal (in pink) enzymes (right panel). (C) Panel of Western blots representing changes in expression of protein involved in steroidogenesis. Results are means ± standard deviation (s.d.); *n* = 5 mice per group, *P < 0.05, **P < 0.01, ***P < 0.001.

STAR is a protein involved in the transport of cholesterol from the outer to the inner mitochondrial membrane and specific partial loss-of-function mutations in *STAR* account for 10% of FGD cases (Meimaridou *et al*. 2013). Similarly, “mild” mutations in the cholesterol side chain cleavage enzyme (*CYP11A1*, the first enzyme in the steroid pathway), can give rise to FGD (Rubtsov *et al*. 2009, Parajes *et al*. 2011, Sahakitrungruang *et al*. 2011). Mutations in 11β-hydroxylase (*CYP11B1*, the last enzyme in the glucocorticoid pathway) cause congenital adrenal hyperplasia. Previous work revealed that oxidative stress, resulting from the application of exogenous ROS, lead to inhibition of STAR protein expression and steroidogenesis in MA-10 Leydig cells, with no effect on CYP11A1 (Diemer *et al*. 2003). We hypothesized that a similar phenomenon might occur with endogenous mitochondrial oxidative stress resulting from NNT deficit affecting *Star* and/or the mitochondrial CYP450 enzymes, *Cyp11a1* and *Cyp11b1/b2*. RNA-seq and Western blot analysis showed no significant changes in the expression of *Star* at mRNA or protein level, indicating that a defect in cholesterol transport due to oxidative insult is an unlikely mechanism (Fig. 7A and C). However, a suggestive 25% decrease in *Cyp11a1*, *Cyp11b1* and *Cyp11b2* mRNA levels in *Nnt^−^^/^^−^* mice with incomplete recovery in *Nnt*^BAC^ suggested a possible mechanism for steroid depletion. In agreement with this we observed a 65% reduction in CYP11A1 at protein level in *Nnt^−^^/^^−^* and partial restoration (to approx. 50% of *Nnt^+/+^* levels) in *Nnt*^BAC^ (Fig. 7A and C). We were unable to assess CYP11B1/B2 levels since no specific murine antibody exists. The failure to completely recover Cyp11a1 levels in the *Nnt*^BAC^ mice may be due to redox imbalance in these overexpressing mice and, significantly, protein abundance mirrors the levels of corticosterone in the three mouse substrains (Figs 3A and 7A). This is analogous to partial loss-of-function mutations in *CYP11A1*, which give rise to adrenal insufficiency in humans; the proteins may retain 30–40% of wild-type activity this is insufficient to maintain normal cortisol production (Parajes *et al*. 2011).

In contrast the intermediate steps of steroidogenesis occur in the ER, once pregnenolone is synthesized it undergoes reactions catalysed by 3β-hydroxysteroid dehydrogenase (*Hsd3b2*) and 21-hydroxylase (*Cyp21a1*) to produce progesterone and deoxycorticosterone respectively (Fig. 7B). No significant changes were observed at mRNA or protein level in these enzymes between *Nnt*^−/−^ vs *Nnt^+/+^* mice, suggesting that mitochondrial ROS does not affect them and that a reduction of these enzymes is not the reason for their corticosterone deficiency (Fig. 7A and C).

### Transcriptomics-differentially expressed genes

In total 400 genes were differentially expressed in pairwise analyses between the mouse substrains (Supplementary Table 2). Differentially expressed genes between *Nnt*^+/+^ vs *Nnt*^−/−^ numbered 187 (89 up and 98 downregulated), between *Nnt*^−/−^ vs *Nnt^BAC^* numbered 157 (130 up- and 37 downregulated) and between *Nnt*^+/+^ vs *Nnt^BAC^* numbered 141 (119 up- and 22 downregulated) (Supplementary Tables 3, 4, 5, 6, 7 and 8).

**Figure 8:**
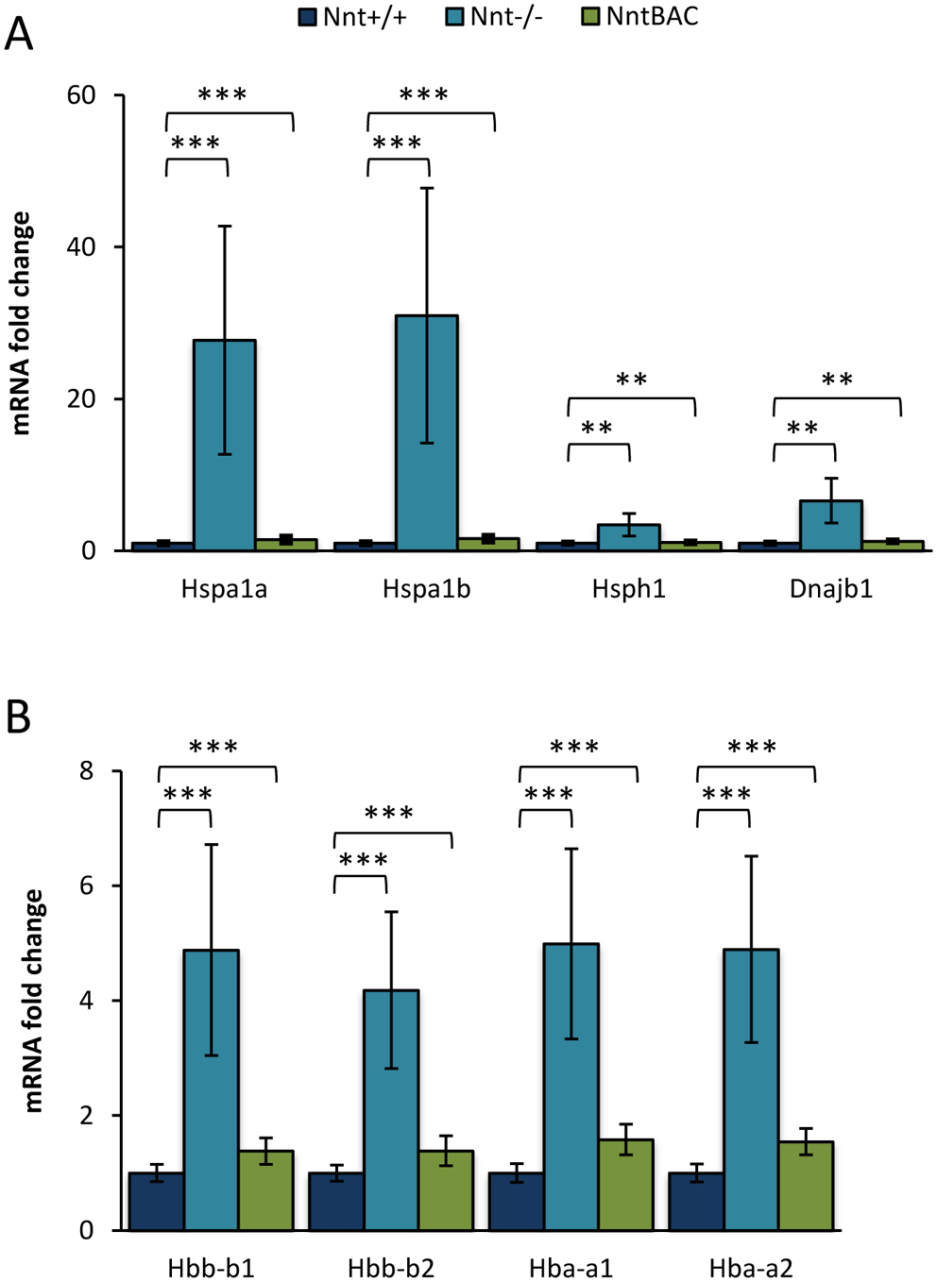
Enrichment of gene pathways in response to oxidative stress. (A) mRNA levels of heat shock proteins revealed significant upregulation (*Hspa1a* 26-fold; *Hspa1a* 25-fold; *Hsph1* 3-fold; *Dnajb1* 6-fold) in *Nnt^−^^/^^−^* mice and restoration of the levels in *Nnt^BAC^* mouse adrenals. (B) Similarly haemoglobin gene mRNA expression was significantly upregulated in *Nnt^−^^/^^−^* mice when compared to *Nnt^+/+^* and reversed in *Nnt^BAC^* (*Hbb-b1* 4-fold; *Hbb-b2* 5-fold; *Hba-a1* 5-fold; *Hba-a2* 5-fold).

We hypothesized that genes with altered expression in *Nnt*^−/−^ that reverted to wild-type levels in the *Nnt*^BAC^ would be genes that were modulated by *Nnt* levels. 40 genes including *Nnt* were altered in *Nnt*^−/−^ with their expression levels rescued in *Nnt*^BAC^. 23 of these were downregulated in *Nnt*^−/−^ and back up in *Nnt*^BAC^, while 17 were upregulated in *Nnt*^−/−^ and back down in *Nnt*^BAC^ (Table 1). Two groups of genes were enriched in this list; chaperones and haemoglobins. Specifically, there was a 25-fold increase in *Hspa1a* and *Hspa1b* in *Nnt*^−/−^ mice, and these levels were restored to *Nnt^+/+^* levels in the *Nnt*^BAC^ mice. A smaller, but still significant, increase was observed in another heat shock (*Hspb1*) and a co-chaperone (*Dnajb1*) in *Nnt*^−/−^ mice (Fig. 8A).

**Table 1:** Forty genes modulated by NNT levels.

Genes downregulated in Nnt^−/−^ and restored in Nnt^BAC^	
Gene ID	Gene description
1500015O10Rik	RIKEN cDNA 1500015O10 gene
9330151L19Rik	ENSMUSG00000097061 – uncharacterized protein
A530020G20Rik	RIKEN cDNA A530020G20 gene
C1qtnf6	C1q and tumour necrosis factor related protein 6
Ccdc160	Coiled-coil domain containing 160
Ctxn3	Cortexin 3
Cyp21a2-ps	Cyp21a2 pseudogene
E330017L17Rik	RIKEN cDNA E330017L17 gene
Epb4.1l4aos	Erythrocyte membrane protein band 4.1 like 4a, opposite strand
Gnat2	Guanine nucleotide binding protein, alpha transducing 2
Ism1	Isthmin 1 homolog (zebrafish)
Kcnn2	Potassium intermediate/small conductance calcium-activated channel, subfamily N, member 2
Lilr4b	Leukocyte immunoglobulin-like receptor, subfamily B, member 4B
Ly96	Lymphocyte antigen 96
Nnt	Nicotinamide nucleotide transhydrogenase
Pacsin3	Protein kinase C and casein kinase substrate in neurons 3
Sox12	SRY-box containing gene 12
Steap1	Six transmembrane epithelial antigen of the prostate 1
Trim12c	Tripartite motif-containing 12C
Trim21	Tripartite motif-containing 21
Trim30d	Tripartite motif-containing 30D
Tuft1	Tuftelin 1
Vsnl1	Visinin-like 1
**Genes upregulated in Nnt^−/−^ and restored in Nnt^BAC^**	
Arl4d	ADP-ribosylation factor-like 4D
Cwc25	CWC25 spliceosome-associated protein homolog (S. cerevisiae)
Cyr61	Cysteine rich protein 61
Dnajb1	DnaJ (Hsp40) homolog, subfamily B, member 1
Egr1	Early growth response 1
Fam46a	Family with sequence similarity 46, member A
Gadd45g	Growth arrest and DNA-damage-inducible 45 gamma
Hba-a1	Hemoglobin alpha, adult chain 1
Hba-a2	Hemoglobin alpha, adult chain 2
Hbb-bs	Hemoglobin, beta adult s chain
Hbb-bt	Hemoglobin, beta adult t chain
Ier2	Immediate early response 2
Ier3	Immediate early response 3
Irs2	Insulin receptor substrate 2
Klf4	Kruppel-like factor 4 (gut)
Nr4a2	Nuclear receptor subfamily 4, group A, member 2
Zfp36l2	Zinc finger protein 36, C3H type-like 2

The upregulation of heat shock proteins suggests that proteins are undergoing damage due to increased ROS and the molecular chaperone machinery is activated to correct or degrade such damaged or misfolded proteins. Interestingly alpha- and beta-haemoglobins (*Hba-a1*, *Hba-a2*, *Hbb-b1* and *Hbb-b2* (*aka Hbb-bs* and *Hbb-bt*)) were 4 to 5-fold upregulated in *Nnt*^−/−^ mice (*P* < 0.025) and their levels returned to normal in *Nnt*^BAC^ (Fig. 8B). Erythroid contamination of the tissues from Nnt^−/−^ mice was considered but ruled out as other genes highly expressed in the development of erythroid lineages were not significantly upregulated (51 genes from An *et al*. 2014). Recently the expression of haemoglobins in tissues other than erythrocytes has been reported suggesting their role in other basic cellular functions apart from O_2_ transport (Fordel *et al*. 2006, Vinogradov & Moens 2008).

## Discussion

We have previously shown that mutations in NNT cause adrenal dysfunction in humans primarily affecting the ZF cells of the adrenal cortex responsible for cortisol production, and observed a 50% reduction in corticosterone levels in 3-month-old *Nnt* null mice (Meimaridou *et al*. 2012). Further, we showed mitochondrial perturbations and limited antioxidant capacity in human adrenocortical carcinoma cells where NNT expression was stably knocked down (Meimaridou *et al*. 2012). In this study, we investigated the mechanism by which NNT affects steroidogenesis in older mice by utilising three models with differing expression levels of *Nnt*; wild-type *Nnt^+/+^*, null *Nnt*^−/−^ and two-fold overexpressing *Nnt^BAC^* mice. Gene expression and Western blotting analysis revealed restricted levels of key mitochondrial antioxidant and steroidogenic proteins in *Nnt*^−/−^ mice leading to glucocorticoid deficiency, which was partially rescued in the overexpressing mice. Interestingly, we demonstrate for the first time that overexpression of *Nnt* also negatively impacts steroidogenesis; this may be due to a persistent redox imbalance initiated by the oversupply of NADPH by NNT.

The mouse inbred C57BL/6 strain is widely used for genetic and functional studies. There are two substrains of these mice depending on their site of origin; C57BL/6J established in Jackson laboratory and C57BL/6N line from the National Institutes of Health (NIH). In 2005, a spontaneous loss-of-function *Nnt* mutation in C57BL/6J was characterised, which was associated with impaired glucose tolerance (Ronchi *et al*. 2016). Since then, these mice have been used to clarify the roles of NNT in mammalian biology.

More recently, comparative genomics between C57BL/6J and C57BL/6N strains has identified many SNPs and structural variants that may contribute to the phenotypic differences between the two strains (24). We compared expression levels of the genes noted in this publication but revealed no differences in mRNA levels exception for Nnt. This suggests that the transcriptome changes and the endocrine phenotype observed in Nnt^−/−^ mice is largely due to differential NNT levels.

In this study, we have employed, in addition, a mouse strain with transgenic rescue of *Nnt* expression (*Nnt^BAC^*) (Freeman *et al*. 2006). The *Nnt* replacement has previously been shown to induce improvements in glucose tolerance and insulin secretion rescuing the phenotype seen in *Nnt*^−/−^ mice. Corticosterone levels recovered somewhat in the *Nnt^BAC^*; however, they remained significantly lower than wild-type levels.

In this *in vivo* model, we also showed that the antioxidant capacity of the *Nnt*^−/−^ adrenals is significantly compromised when compared to *Nnt^+/+^* counterparts. The protein levels of key mitochondrial antioxidant enzymes PRDX3 and TXNRD2 are significantly reduced in *Nnt*^−/−^ mice and fail to recover in *Nnt*^BAC^. This strongly suggests that a set level of Nnt expression is required to maintain mitochondrial redox homeostasis. Furthermore, since mitochondrial NADPH can be regenerated not only by Nnt, but also by isocitrate dehydrogenase 2 (Idh2) and malic enzyme 3 (Me3), we excluded a possible compensatory mechanism, as expression levels of these enzymes remain unchanged between *Nnt^+/+^* and *Nnt*^−/−^ mice. Studies by other groups have similarly demonstrated that liver, heart, brain and skeletal muscle mitochondria from *Nnt*^−/−^ mice have unaltered Idh2 and Me3 enzymatic activities meaning they cannot compensate for the loss of NNT to restore NADPH levels (Nickel *et al*. 2015, Ronchi *et al*. 2016). When *NNT* is ablated in human adrenocortical cells, we also see a disturbance of redox balance, but here this does not affect cortisol output, perhaps due to adaptive alterations in sulfiredoxin and peroxiredoxin III levels, which are known to occur in H295R cells, and even primary adrenocortical cells, when grown in culture (Kil *et al*. 2013).

NNT dysregulation not only affects the antioxidant capacity of the adrenal but also its steroidogenic capacity. We demonstrated that, in *Nnt*^−/−^ mice, adrenal steroidogenesis is severely affected (86% reduction in corticosterone in 18 months old mice), due to the low protein levels of a crucial steroidogenic enzyme, CYP11A1. Interestingly, *Nnt*^BAC^ mice also exhibit glucocorticoid deficiency as indicated by 60% reduction in levels of corticosterone suggesting that *Nnt* overexpression also impacts on the steroidogenic output of these mice. It is increasingly recognised that redox balance is key to physiological health. Where one might assume that underexpression of antioxidants would lead to oxidative stress and overexpression would give reductive stress; this is not necessarily what occurs *in vivo* (reviewed in Lei *et al*. 2016), with clear examples of antioxidant gene knockdown inducing reductive stress (Yan *et al*. 2017) and antioxidant gene overexpression also causing reductive stress (Zhang *et al*. 2010). In addition, paradoxically, both reductive and oxidative insult can lead to overproduction of ROS (Barrett *et al*. 2004, Arrigo *et al*. 2005, Filomeni *et al*. 2005, Ali *et al*. 2014, Yu *et al*. 2014, Korge *et al*. 2015), which can cause protein damage. We suggest that this may be the explanation for the failure to rescue glucocorticoid secretion in the overexpressing mice with the demonstration of higher levels of lipid peroxidation in the Nnt^BAC^ mice compared to Nnt^+/+^ mice lending support to this.

Differential gene expression studies between *Nnt^+/+^* and *Nnt*^−/−^ revealed a significant upregulation of heat shock proteins in *Nnt*^−/−^ mice. The failure of ROS detoxification may lead to oxidative damage of proteins and the canonical chaperone machinery will be upregulated to cope with the resultant protein misfolding and degradation. The increased ROS will also render cells more susceptible to apoptosis but heat shock proteins 27 and 70 (Hsp27 and Hsp70 respectively) are activated by mitochondrial ROS and are protective of cells preventing apoptosis by replenishing reduced glutathione and reducing intracellular iron levels (Barrett *et al*. 2004, Arrigo *et al*. 2005, Filomeni *et al*. 2005).

In addition to heat shock protein machinery, we show haemoglobins are regulated by NNT levels. There is a significant increase in the mRNA levels of haemoglobins in *Nnt*^−/−^ mice possibly as a compensatory mechanism to combat oxidative stress. Haemoglobins are composed of alpha and beta HbA chains and their accepted main function is to transport O_2_ to cells thorough the vascular network. However, their involvement in other fundamental cellular functions and in non-erythroid cells is increasingly being recognised. Detection of haemoglobin chains in macrophages, alveolar cells, kidney, brain and vaginal epithelial cells has been reported and their function has been linked with antioxidant defence and the regulation of mitochondrial activity (Liu *et al*. 1999, Arrigo *et al*. 2005, Newton *et al*. 2006, Nishi *et al*. 2008, Saha *et al*. 2017). In adrenal, overexpression of *α*-Hb in rat phaeochromocytoma (PC12) cells resulted in downregulation of *Gpx1* and *Sod1* mRNAs suggesting that it may have a role in the scavenging of ROS (Biagioli *et al*. 2009, Maria *et al*. 2012). Whether a similar mechanism explains the upregulation we see in intact adrenals requires further investigation.

In this study, we showed that the reduced steroidogenic capacity of the adrenals in *Nnt^−^^/^^−^* and *Nnt*^BAC^ mice is due to the inability of other antioxidant enzymes to compensate for redox imbalance resulting from altered Nnt levels. This leads to limited availability of mitochondrial CYP11A1 and a reduction in corticosterone output. Recently a similar mechanism was demonstrated to underlie the adrenal dysfunction seen in Triple A, a disorder of adrenal insufficiency, alacrima and achalasia due to mutations in *AAAS* encoding the protein ALADIN. A deficiency of ALADIN results in cytosolic, as opposed to mitochondrial, oxidative stress and a deficit of microsomal, rather than mitochondrial, CYP450 enzymes thereby retarding adrenal steroidogenesis (Juhlen *et al*. 2015).

## Conclusions

Using transcriptomic profiling in adrenals from three mouse substrains, we showed that disturbances in adrenal redox homeostasis are mediated not only by under expression of NNT but also by its overexpression. Further, we demonstrated that both underexpression or overexpression of NNT reduces corticosterone output implying a central role for it in the control of steroidogenesis. Reduced expression of CYP11A1, a key mitochondrial steroidogenic enzyme, mirrored the reduction in corticosterone output. Our data also suggest that oxidative stress and/or ROS damage to proteins is activating mito- and cytoprotective proteins (haemoglobins and heat shock proteins respectively) that may help maintain cell viability but do not rescue the steroidogenic phenotype.

## Supplementary Material

Supporting Table 1

Supporting Table 2

Supporting Table 3

Supporting Table 4

Supporting Table 5

Supporting Table 6

Supporting Table 7

Supporting Table 8

## Declaration of interest

The authors declare that there is no conflict of interest that could be perceived as prejudicing the impartiality of the research reported.

## Funding

This work has been supported by the Medical Research Council UK (Project Grant MR/K020455/1, to L A M), (Project Grant MC_U142661184, to R C) and the Wellcome Trust (Clinical Research Training Fellowship WT101671, to V C).

## Author contribution statement

E M, L A M designed the study and analysed the RNA-seq data. E M and F E performed the immunoblotting and mouse histology. M G and R C generated and validated mouse substrains. V C, P A F and W A performed the steroidogenic profiling of mice and analysed the mass spectrometric data. E M and L A M prepared the draft manuscript. All authors contributed to the discussion of results and edited and approved the final manuscript.
