# Supplementary material for: NNT is a key regulator of adrenal redox homeostasis and steroidogenesis in male mice
Source: J Endocrinol. 2017 Oct 18;236(1):13–28. doi: 10.1530/JOE-16-0638 (PMC5744559; doi:10.1530/JOE-16-0638)
Supplement: Supporting Table 2 [file erc-236-13-t002.pdf]

Supplementary Table 2. 400 genes differentially expressed between *Nnt*$^{+/+}$, *Nnt*$^{-/-}$ and *Nnt*^{BAC}

Gene ID	Gene description
1500015O10Rik	Ecrg4 - RIKEN cDNA 1500015O10 gene; Probable hormone that may induce senescence of oligodendrocyte and neural precursor cells, characterized by G1 arrest, RB1 dephosphorylation and accelerated CCND1 and CCND3 proteasomal degradation
1600002H07Rik	ENSMUSG00000024118 - RIKEN cDNA 1600002H07 gene
1700019G17Rik	ENSMUSG00000068299 - RIKEN cDNA 1700019G17 gene
2010109I03Rik	ENSMUSG00000063522 - RIKEN cDNA 2010109I03 gene
2300009A05Rik	ENSMUSG00000032403 - RIKEN cDNA 2300009A05 gene
2610524H06Rik	ENSMUSG00000092486 - RIKEN cDNA 2610524H06 gene
4921524J17Rik	ENSMUSG00000036934 - RIKEN cDNA 4921524J17 gene
4930426D05Rik	RIKEN cDNA 4930426D05 gene
9330151L19Rik	ENSMUSG00000097061 - Uncharacterized protein
A330023F24Rik	RIKEN cDNA A330023F24 gene
A530020G20Rik	RIKEN cDNA A530020G20 gene
AA474331	AA474331 - Uncharacterized protein
AA986860	AA986860 - expressed sequence AA986860; Putative androgen-specific receptor (By similarity)
Abi3bp	Abi3bp - ABI gene family, member 3 (NESH) binding protein
AC153941.1	Gene AC153941.1
Adamts1	Adamts1 - a disintegrin-like and metallopeptidase (reprolysin type) with thrombospondin type 1 motif, 1; Cleaves aggrecan, a cartilage proteoglycan, and may be involved in its turnover. Has angiogenic inhibitor activity (By similarity). Active metalloprotease, which may be associated with various inflammatory processes as well as development of cancer cachexia. May play a critical role in follicular rupture (By similarity)
Adprhl2	Adprhl2 - ADP-ribosylhydrolase like 2; Poly(ADP-ribose) synthesized after DNA damage is only present transiently and is rapidly degraded by poly(ADP-ribose) glycohydrolase. Poly(ADP-ribose) metabolism may be required for maintenance of the normal function of neuronal cells. Generates ADP-ribose from poly-(ADP-ribose), but does not hydrolyze ADP- ribose-arginine, -cysteine, -diphthamide, or -asparagine bonds. Due to catalytic inactivity of PARG mitochondrial isoforms, ARH3 is the only PAR hydrolyzing enzyme in mitochondria (By similarity)
Ajuba	Jub - ajuba; Adapter or scaffold protein which participates in the assembly of numerous protein complexes and is involved in several cellular processes such as cell fate determination, cytoskeletal organization, repression of gene transcription, mitosis, cell-cell adhesion, cell differentiation, proliferation and migration. Contributes to the linking and/or strengthening of epithelia cell- cell junctions in part by linking adhesive receptors to the actin cytoskeleton. May be involved in signal transduction from cell adhesion sites to the nucleus.
Akip1	Akip1 - A kinase (PRKA) interacting protein 1; Enhances NF-kappa-B transcriptional activity by regulating the nuclear localization of the NF-kappa-B subunit RELA and promoting the phosphorylation of RELA by PRKACA. Regulates the effect of the cAMP-dependent protein kinase signaling pathway on the NF-kappa-B activation cascade (By similarity)

Akr1cl	Akr1cl - aldo-keto reductase family 1, member C-like
Aldh1a1	Aldh1a1 - aldehyde dehydrogenase family 1, subfamily A1; In addition to the activity on acetaldehyde and related substrates, is also involved in the oxidation of aldehydes derived from biogenic amines such as epinephrine and norepinephrine, as well as the aldehydes generated via lipid peroxidation. Binds free retinal and cellular retinol-binding protein-bound retinal. Can convert/oxidize retinaldehyde to retinoic acid (By similarity)
Aldh3b1	Aldh3b1 - aldehyde dehydrogenase 3 family, member B1; Oxidizes medium and long chain saturated and unsaturated aldehydes (By similarity). Metabolizes also benzaldehyde (By similarity). Low activity towards acetaldehyde and 3,4- dihydroxyphenylacetaldehyde (By similarity). May not metabolize short chain aldehydes (By similarity). May use both NADP(+) and NAD(+) as cofactors (By similarity). May have a protective role against the cytotoxicity induced by lipid peroxidation (By similarity)
Alox5ap	Alox5ap - arachidonate 5-lipoxygenase activating protein; Required for leukotriene biosynthesis by ALOX5 (5- lipoxygenase). Anchors ALOX5 to the membrane. Binds arachidonic acid, and could play an essential role in the transfer of arachidonic acid to ALOX5. Binds to MK-886, a compound that blocks the biosynthesis of leukotrienes (By similarity). unstructured (By similarity)
Angptl4	Angptl4 - angiopoietin-like 4; Protein with hypoxia-induced expression in endothelial cells. May act as a regulator of angiogenesis and modulate tumorigenesis. Inhibits proliferation, migration, and tubule formation of endothelial cells and reduces vascular leakage. May exert a protective function on endothelial cells through an endocrine action. It is directly involved in regulating glucose homeostasis, lipid metabolism, and insulin sensitivity (By similarity). In response to hypoxia, the unprocessed form of the protein accumulates in the subendothelial extracellular matrix (ECM).
Arhgdib	Arhgdib - Rho, GDP dissociation inhibitor (GDI) beta; Regulates the GDP/GTP exchange reaction of the Rho proteins by inhibiting the dissociation of GDP from them, and the subsequent binding of GTP to them
Arl4d	Arl4d - ADP-ribosylation factor-like 4D; Small GTP-binding protein which cycles between an inactive GDP-bound and an active GTP-bound form, and the rate of cycling is regulated by guanine nucleotide exchange factors (GEF) and GTPase-activating proteins (GAP). GTP-binding protein that does not act as an allosteric activator of the cholera toxin catalytic subunit. Recruits CYTH1, CYTH2, CYTH3 and CYTH4 to the plasma membrane in GDP-bound form (By similarity)
Arrdc2	Arrdc2 - arrestin domain containing 2
Aspn	Aspn - asporin; Binds calcium and plays a role in osteoblast-driven collagen biomineralization activity (By similarity). Critical regulator of TGF-beta in articular cartilage and plays an essential role in cartilage homeostasis and osteoarthritis (OA) pathogenesis. Negatively regulates chondrogenesis in the articular cartilage by blocking the TGF-beta/receptor interaction on the cell surface and inhibiting the canonical TGF-beta/Smad signal. Negatively regulates periodontal ligament (PDL) differentiation and mineralization to ensure that the PDL is not ossified

Atp5d	Atp5d - ATP synthase, H+ transporting, mitochondrial F1 complex, delta subunit; Mitochondrial membrane ATP synthase (F(1)F(0) ATP synthase or Complex V) produces ATP from ADP in the presence of a proton gradient across the membrane which is generated by electron transport complexes of the respiratory chain. F-type ATPases consist of two structural domains, F(1) - containing the extramembraneous catalytic core, and F(0) - containing the membrane proton channel, linked together by a central stalk and a peripheral stalk.
B930041F14Rik	MNCb-3966 - RIKEN cDNA B930041F14 gene
BC002059	BC002059 - cDNA sequence BC002059
Bcl2a1b	Bcl2a1b - B cell leukemia/lymphoma 2 related protein A1b
Bcl2l12	Bcl2l12 - BCL2-like 12 (proline rich)
Bloc1s3	Bloc1s3 - biogenesis of lysosome-related organelles complex-1, subunit 3; Component of the BLOC-1 complex, a complex that is required for normal biogenesis of lysosome-related organelles (LRO), such as platelet dense granules and melanosomes. In concert with the AP-3 complex, the BLOC-1 complex is required to target membrane protein cargos into vesicles assembled at cell bodies for delivery into neurites and nerve terminals. The BLOC-1 complex, in association with SNARE proteins, is also proposed to be involved in neurite extension. Plays a role in intracellular vesicle trafficking
Bmp7	Bmp7 - bone morphogenetic protein 7; Induces cartilage and bone formation. May be the osteoinductive factor responsible for the phenomenon of epithelial osteogenesis. Plays a role in calcium regulation and bone homeostasis
Btg2	Btg2 - B cell translocation gene 2, anti-proliferative; Anti-proliferative protein; the function is mediated by association with deadenylase subunits of the CCR4-NOT complex. Activates mRNA deadenyltion in a CNOT6 and CNOT7-dependent manner. In vitro can inhibit deadenylase activity of CNOT7 and CNOT8. Involved in cell cycle regulation. Could be involved in the growth arrest and differentiation of the neuronal precursors. Modulates transcription regulation mediated by ESR1. Involved in mitochondrial depolarization and neurite outgrowth (By similarity)
C1qtnf6	C1qtnf6 - C1q and tumor necrosis factor related protein 6
C6	C6 - complement component 6
C920006O11Rik	RIKEN cDNA C920006O11 gene
Cacna1c	Cacna1c - calcium channel, voltage-dependent, L type, alpha 1C subunit; Voltage-sensitive calcium channels (VSCC) mediate the entry of calcium ions into excitable cells and are also involved in a variety of calcium-dependent processes, including muscle contraction, hormone or neurotransmitter release, gene expression, cell motility, cell division and cell death. The isoform alpha-1C gives rise to L-type calcium currents. Long-lasting (L-type) calcium channels belong to the 'high-voltage activated' (HVA) group.
Car2	Car2 - carbonic anhydrase 2; Essential for bone resorption and osteoclast differentiation (By similarity). Reversible hydration of carbon dioxide
Cartpt	Cartpt - CART prepropeptide; Satiety factor closely associated with the actions of leptin and neuropeptide y; this anorectic peptide inhibits both normal and starvation-induced feeding and completely blocks the feeding response induced by neuropeptide Y and regulated by leptin in the hypothalamus (By similarity)

Cbarp	Calcium channel, voltage-dependent, beta subunit associated regulatory protein
Ccdc114	Ccdc114 - coiled-coil domain containing 114; Probable component of the outer dynein arm complex required along the entire axoneme for tethering of outer dynein arms (By similarity)
Ccdc122	Ccdc122 - coiled-coil domain containing 122
Ccdc160	Ccdc160 - coiled-coil domain containing 160
Ccdc51	Ccdc51 - coiled-coil domain containing 51
Ccdc8	Ccdc8 - coiled-coil domain containing 8
Cd248	Cd248 - CD248 antigen, endosialin; May play a role in angiogenesis or vascular function
Cd300lh	Cd300lh - CD300 antigen like family member H; Acts as an activating receptor inducing cytokine production in mast cells. Can act as a positive regulator of TLR9 signaling in macrophages, leading to enhanced production of proinflammatory cytokines
Cd44	Cd44 - CD44 antigen
Cd48	Cd48 - CD48 antigen; Ligand for CD2. Might facilitate interaction between activated lymphocytes. Probably involved in regulating T-cell activation
Cd52	Cd52 - CD52 antigen; May play a role in carrying and orienting carbohydrate, as well as having a more specific role
Cd53	Cd53 - CD53 antigen; May be involved in growth regulation in hematopoietic cells
Cd59a	Cd59a - CD59a antigen; Potent inhibitor of the complement membrane attack complex (MAC) action. Acts by binding to the C8 and/or C9 complements of the assembling MAC, thereby preventing incorporation of the multiple copies of C9 required for complete formation of the osmolytic pore (By similarity)
Cdc42ep3	Cdc42ep3 - CDC42 effector protein (Rho GTPase binding) 3; Probably involved in the organization of the actin cytoskeleton. May act downstream of CDC42 to induce actin filament assembly leading to cell shape changes. Induces pseudopodia formation in fibroblasts (By similarity)
Cdh11	Cdh11 - cadherin 11; Cadherins are calcium-dependent cell adhesion proteins. They preferentially interact with themselves in a homophilic manner in connecting cells; cadherins may thus contribute to the sorting of heterogeneous cell types
Cdkn2b	Cdkn2b - cyclin-dependent kinase inhibitor 2B (p15, inhibits CDK4); Interacts strongly with CDK4 and CDK6. Potent inhibitor. Potential effector of TGF-beta induced cell cycle arrest (By similarity)
Ceacam1	Ceacam1 - carcinoembryonic antigen-related cell adhesion molecule 1; Unknown. In case of murine coronavirus (MHV) infection, serves as receptor for MHV S1 spike glycoprotein
Cenpt	Cenpt - centromere protein T; Component of the CENPA-NAC (nucleosome-associated) complex, a complex that plays a central role in assembly of kinetochore proteins, mitotic progression and chromosome segregation. The CENPA-NAC complex recruits the CENPA-CAD (nucleosome distal) complex and may be involved in incorporation of newly synthesized CENPA into centromeres. Part of a nucleosome-associated complex that binds specifically to histone H3- containing nucleosomes at the centromere, as opposed to nucleosomes containing CENPA
Cep85	Centrosomal protein 85; Ccdc21 - coiled-coil domain containing 21

Cfl2	Cfl2 - cofilin 2, muscle; Controls reversibly actin polymerization and depolymerization in a pH-sensitive manner. It has the ability to bind G- and F-actin in a 1:1 ratio of cofilin to actin. It is the major component of intranuclear and cytoplasmic actin rods
Cilp	Cilp - cartilage intermediate layer protein, nucleotide pyrophosphohydrolase; Probably plays a role in cartilage scaffolding. May act by antagonizing TGF-beta1 (TGFB1) and IGF1 functions. Has the ability to suppress IGF1-induced proliferation and sulfated proteoglycan synthesis, and inhibits ligand-induced IGF1R autophosphorylation. May inhibit TGFB1-mediated induction of cartilage matrix genes via its interaction with TGFB1. Overexpression may lead to impair chondrocyte growth and matrix repair and indirectly promote inorganic pyrophosphate (PPi) supersaturation in aging and osteoarthritis
Cldn1	Cldn1 - claudin 1; Plays a major role in tight junction-specific obliteration of the intercellular space, through calcium- independent cell-adhesion activity
Cldn5	Cldn5 - claudin 5; Plays a major role in tight junction-specific obliteration of the intercellular space, through calcium- independent cell-adhesion activity (By similarity)
Clec3b	Clec3b - C-type lectin domain family 3, member b; Tetranectin binds to plasminogen and to isolated kringle 4. May be involved in the packaging of molecules destined for exocytosis (By similarity)
Cml5	Cml5 - camello-like 5; May play a role in regulation of gastrulation
Cmtm7	Cmtm7 - CKLF-like MARVEL transmembrane domain containing 7
Cnr1	Cnr1 - cannabinoid receptor 1 (brain); Involved in cannabinoid-induced CNS effects. Acts by inhibiting adenylate cyclase. Could be a receptor for anandamide. Inhibits L-type Ca(2+) channel current
Col1a1	Col1a1 - collagen, type I, alpha 1; Type I collagen is a member of group I collagen (fibrillar forming collagen)
Col1a2	Col1a2 - collagen, type I, alpha 2; Type I collagen is a member of group I collagen (fibrillar forming collagen)
Col3a1	Col3a1 - collagen, type III, alpha 1; Collagen type III occurs in most soft connective tissues along with type I collagen
Col6a2	Col6a2 - collagen, type VI, alpha 2; Collagen VI acts as a cell-binding protein
Commd5	Commd5 - COMM domain containing 5; Down-regulates activation of NF-kappa-B (By similarity)
Cpne8	Cpne8 - copine VIII; May function in membrane trafficking. Exhibits calcium- dependent phospholipid binding properties (By similarity)
Cryab	Cryab - crystallin, alpha B; May contribute to the transparency and refractive index of the lens. Has chaperone-like activity, preventing aggregation of various proteins under a wide range of stress conditions
Cryba4	Cryba4 - crystallin, beta A4; Crystallins are the dominant structural components of the vertebrate eye lens
Csrnp1	Csrnp1 - cysteine-serine-rich nuclear protein 1; Binds to the consensus sequence 5'-AGAGTG-3' and has transcriptional activator activity. May have a tumor-suppressor function. May play a role in apoptosis
Ctxn3	Ctxn3 - cortexin 3
Cwc25	Cwc25 - CWC25 spliceosome-associated protein homolog (S. cerevisiae)

Cybb	Cybb - cytochrome b-245, beta polypeptide; Critical component of the membrane-bound oxidase of phagocytes that generates superoxide. It is the terminal component of a respiratory chain that transfers single electrons from cytoplasmic NADPH across the plasma membrane to molecular oxygen on the exterior. Also functions as a voltage-gated proton channel that mediates the H(+) currents of resting phagocytes
Cyp1b1	Cyp1b1 - cytochrome P450, family 1, subfamily b, polypeptide 1; Cytochromes P450 are a group of heme-thiolate monooxygenases. In liver microsomes, this enzyme is involved in an NADPH-dependent electron transport pathway. It oxidizes a variety of structurally unrelated compounds, including steroids, fatty acids, and xenobiotics
Cyp21a2-ps	Cyp21a1 - cytochrome P450, family 21, subfamily a, polypeptide 1; Specifically catalyzes the 21-hydroxylation of steroids. Required for the adrenal synthesis of mineralocorticoids and glucocorticoids
Cyp7b1	Cyp7b1 - cytochrome P450, family 7, subfamily b, polypeptide 1
Cyr61	Cyr61 - cysteine rich protein 61; Promotes cell proliferation, chemotaxis, angiogenesis and cell adhesion. Appears to play a role in wound healing by up- regulating, in skin fibroblasts, the expression of a number of genes involved in angiogenesis, inflammation and matrix remodeling including VEGA-A, VEGA-C, MMP1, MMP3, TIMP1, uPA, PAI-1 and integrins alpha-3 and alpha-5 (By similarity). CYR61-mediated gene regulation is dependent on heparin-binding (By similarity). Down-regulates the expression of alpha-1 and alpha-2 subunits of collagen type-1 (By similarity).
Dbp	Dbp - D site albumin promoter binding protein; This transcriptional activator recognizes and binds to the sequence 5'-RTTAYGTAAY-3' found in the promoter of genes such as albumin, CYP2A4 and CYP2A5. It is not essential for circadian rhythm generation, but modulates important clock output genes. May be a direct target for regulation by the circadian pacemaker component clock. May affect circadian period and sleep regulation (By similarity)
Dcn	Dcn - decorin; May affect the rate of fibrils formation
Ddx58	Ddx58 - DEAD (Asp-Glu-Ala-Asp) box polypeptide 58; Innate immune receptor which acts as a cytoplasmic sensor of viral nucleic acids and plays a major role in sensing viral infection and in the activation of a cascade of antiviral responses including the induction of type I interferons and proinflammatory cytokines. Its ligands include: 5'-triphosphorylated ssRNA and dsRNA and short dsRNA (<1 kb in length). In addition to the 5'-triphosphate moiety, blunt-end base pairing at the 5'-end of the RNA is very essential.
Des	Des - desmin; Desmin are class-III intermediate filaments found in muscle cells. In adult striated muscle they form a fibrous network connecting myofibrils to each other and to the plasma membrane from the periphery of the Z-line structures
Dgat1	Dgat1 - diacylglycerol O-acyltransferase 1; Catalyzes the terminal and only committed step in triacylglycerol synthesis by using diacylglycerol and fatty acyl CoA as substrates. In contrast to DGAT2 it is not essential for survival. May be involved in VLDL (very low density lipoprotein) assembly. In liver, plays a role in esterifying exogenous fatty acids to glycerol. Functions as the major acyl-CoA retinol acyltransferase (ARAT) in the skin, where it acts to maintain retinoid homeostasis and prevent retinoid toxicity leading to skin and hair disorders
Dhrs3	Dhrs3 - dehydrogenase/reductase (SDR family) member 3; Catalyzes the reduction of all-trans-retinal to all- trans-retinol in the presence of

	NADPH (By similarity)
Dhx58	Dhx58 - DEXH (Asp-Glu-X-His) box polypeptide 58; Acts as a regulator of DDX58/RIG-I and IFIH1/MDA5 mediated antiviral signaling. Cannot initiate antiviral signaling as it lacks the CARD domain required for activating MAVS/IPS1- dependent signaling events. Can have both negative and positive regulatory functions related to DDX58/RIG-I and IFIH1/MDA5 signaling and this role in regulating signaling may be complex and could probably depend on characteristics of the infecting virus or target cells, or both.
Dnajb1	Dnajb1 - DnaJ (Hsp40) homolog, subfamily B, member 1; Interacts with HSP70 and can stimulate its ATPase activity. Stimulates the association between HSC70 and HIP (By similarity)
Dpt	Dpt - dermatopontin; Seems to mediate adhesion by cell surface integrin binding. May serve as a communication link between the dermal fibroblast cell surface and its extracellular matrix environment. Enhances TGFB1 activity (By similarity). Inhibits cell proliferation. Accelerates collagen fibril formation, and stabilizes collagen fibrils against low-temperature dissociation
Dus4l	Dus4l - dihydrouridine synthase 4-like (S. cerevisiae); Catalyzes the synthesis of dihydrouridine, a modified base found in the D-loop of most tRNAs (By similarity)
Dusp1	Dusp1 - dual specificity phosphatase 1; Dual specificity phosphatase that dephosphorylates MAP kinase MAPK1/ERK2 on both 'Thr-183' and 'Tyr-185', regulating its activity during the meiotic cell cycle
Dusp10	Dusp10 - dual specificity phosphatase 10; Protein phosphatase involved in the inactivation of MAP kinases. Has a specificity for the MAPK11/MAPK12/MAPK13/MAPK14 subfamily (By similarity)
Dusp23	Dusp23 - dual specificity phosphatase 23; Protein phosphatase that mediates dephosphorylation of proteins phosphorylated on Tyr and Ser/Thr residues. In vitro, it can dephosphorylate p44-ERK1 (MAPK3) but not p54 SAPK-beta (MAPK10) in vitro. Able to enhance activation of JNK and p38 (MAPK14)
E330017L17Rik	RIKEN cDNA E330017L17 gene
Egr1	Egr1 - early growth response 1; Transcriptional regulator. Recognizes and binds to the DNA sequence 5'-CGCCCCCGC-3'(EGR-site). Activates the transcription of target genes whose products are required for mitogenesis and differentiation
Emp2	Emp2 - epithelial membrane protein 2
Epb4.1l4aos	erythrocyte membrane protein band 4.1 like 4a, opposite strand
Epn3	Epn3 - epsin 3
Etohi1	Etohi1 - ethanol induced 1
Etv1	Etv1 - ets variant gene 1; Transcriptional activator that binds to DNA sequences containing the consensus pentanucleotide 5'-CGGA[AT]-3'
Exo5	Exo5 - exonuclease 5; Single-stranded DNA (ssDNA) bidirectional exonuclease involved in DNA repair. Probably involved in DNA repair following ultraviolet (UV) irradiation and interstrand cross-links (ICLs) damage. Has both 5'-3' and 3'-5' exonuclease activities with a strong preference for 5'-ends. Acts as a sliding exonuclease that loads at ssDNA ends and then slides along the ssDNA prior to cutting; however the sliding and the 3'-5' exonuclease activities are abolished upon binding to the replication protein A (RPA) complex that enforces 5'-directionality activity (By similarity)
Fads6	Fads6 - fatty acid desaturase domain family, member 6

Fam105a	Fam105a - family with sequence similarity 105, member A
Fam46a	Fam46a - family with sequence similarity 46, member A
Fam58b	Fam58b - family with sequence similarity 58, member B; May have a role in cell proliferation (By similarity)
Fbln7	Fbln7 - fibulin 7; An adhesion molecule that interacts with extracellular matrix molecules in developing teeth and may play important roles in differentiation and maintenance of odontoblasts as well as in dentin formation
Fbn1	Fbn1 - fibrillin 1; Fibrillins are structural components of 10-12 nm extracellular calcium-binding microfibrils, which occur either in association with elastin or in elastin-free bundles. Fibrillin-1- containing microfibrils provide long-term force bearing structural support. Regulates osteoblast maturation by controlling TGF-beta bioavailability and calibrating TGF-beta and BMP levels, respectively
Fcgr2b	Fcgr2b - Fc receptor, IgG, low affinity IIb
Fdps	Fdps - farnesyl diphosphate synthetase; Key enzyme in isoprenoid biosynthesis which catalyzes the formation of farnesyl diphosphate (FPP), a precursor for several classes of essential metabolites including sterols, dolichols, carotenoids, and ubiquinones. FPP also serves as substrate for protein farnesylation and geranylgeranylation. Catalyzes the sequential condensation of isopentenyl pyrophosphate with the allylic pyrophosphates, dimethylallyl pyrophosphate, and then with the resultant geranylpyrophosphate to the ultimate product farnesyl pyrophosphate (By similarity)
Fdxr	Fdxr - ferredoxin reductase; Serves as the first electron transfer protein in all the mitochondrial P450 systems. Including cholesterol side chain cleavage in all steroidogenic tissues, steroid 11-beta hydroxylation in the adrenal cortex, 25-OH-vitamin D3-24 hydroxylation in the kidney, and sterol C-27 hydroxylation in the liver
Fmod	Fmod - fibromodulin; Affects the rate of fibrils formation. May have a primary role in collagen fibrillogenesis
Frk	Frk - fyn-related kinase; Non-receptor tyrosine-protein kinase that negatively regulates cell proliferation. Positively regulates PTEN protein stability through phosphorylation of PTEN on 'Tyr-336', which in turn prevents its ubiquitination and degradation, possibly by reducing its binding to NEDD4. May function as a tumor suppressor (By similarity)
Fscn1	Fscn1 - fascin homolog 1, actin bundling protein (Strongylocentrotus purpuratus); Organizes filamentous actin into bundles with a minimum of 4.1:1 actin/fascin ratio. Plays a role in the organization of actin filament bundles and the formation of microspikes, membrane ruffles, and stress fibers. Important for the formation of a diverse set of cell protrusions, such as filopodia, and for cell motility and migration (By similarity)
Gadd45g	Gadd45g - growth arrest and DNA-damage-inducible 45 gamma; Involved in the regulation of growth and apoptosis. Mediates activation of stress-responsive MTK1/MEKK4 MAPKKK
Galnt2	Galnt2 - UDP-N-acetyl-alpha-D-galactosamine:polypeptide N-acetylgalactosaminyltransferase 2; Catalyzes the initial reaction in O-linked oligosaccharide biosynthesis, the transfer of an N-acetyl-D-galactosamine residue to a serine or threonine residue on the protein receptor. Has a broad spectrum of substrates for peptides such as EA2, Muc5AC, Muc1a, Muc1b. Probably involved in O-linked glycosylation of the immunoglobulin A1 (IgA1) hinge region (By similarity)

Gas2	Gas2 - growth arrest specific 2; May play a role in apoptosis by acting as a cell death substrate for caspases. Is cleaved during apoptosis and the cleaved form induces dramatic rearrangements of the actin cytoskeleton and potent changes in the shape of the affected cells. May play a role in chondrocyte proliferation and differentiation, and in limb myogenesis. May be involved in the regulation of the apoptosis in the interdigital tissues of the developing hindlimb. May be involved in the membrane ruffling process
Gem	Gem - GTP binding protein (gene overexpressed in skeletal muscle); Could be a regulatory protein, possibly participating in receptor-mediated signal transduction at the plasma membrane. Has guanine nucleotide-binding activity but undetectable intrinsic GTPase activity
Gemin6	Gemin6 - gem (nuclear organelle) associated protein 6; The SMN complex plays an essential role in spliceosomal snRNP assembly in the cytoplasm and is required for pre-mRNA splicing in the nucleus
Gfra2	Gfra2 - glial cell line derived neurotrophic factor family receptor alpha 2; Receptor for neurturin. Mediates the NRTN-induced autophosphorylation and activation of the RET receptor. Also able to mediate GDNF signaling through the RET tyrosine kinase receptor
Gins2	Gins2 - GINS complex subunit 2 (Psf2 homolog); The GINS complex plays an essential role in the initiation of DNA replication, and progression of DNA replication forks. GINS complex seems to bind preferentially to single- stranded DNA (By similarity)
Gm12840	Predicted gene 12840
Gm13152	Gm13152 - predicted gene 13152
Gm13889	Predicted gene 13889
Gm14306	Gm14306 - predicted gene 14306
Gm14403	Gm14403 - predicted gene 14403
Gm17275	Predicted gene, 17275
Gm21685	Lysal1 - Ectonucleoside triphosphate diphosphohydrolase 4 ; Hydrolyzes preferentially nucleoside 5'-diphosphates, nucleoside 5'-triphosphates are hydrolyzed only to a minor extent (By similarity)
Gm23262	Predicted gene, 23262
Gm2564	Gm2442 - predicted gene 2442
Gm26547	Predicted gene, 26547
Gm26809	Predicted gene, 26809
Gm26880	predicted gene, 26880 known lincRNA
Gm28048	predicted gene, 28048
Gm28382	predicted gene 28382 known lincRNA
Gm6344	Predicted gene 6344
Gm8226	Predicted gene 8226
Gm8430	Rps27a - ribosomal protein S27A
Gm9780	Gm10393 - predicted gene 10393
Gm9938	Gm9938 - predicted gene 9938
Gna14	Gna14 - guanine nucleotide binding protein, alpha 14; Guanine nucleotide-binding proteins (G proteins) are involved as modulators or transducers in various transmembrane signaling systems
Gnat2	Gnat2 - guanine nucleotide binding protein, alpha transducing 2; Guanine nucleotide-binding proteins (G proteins) are involved as modulators or transducers in various transmembrane signaling systems.

	Transducin is an amplifier and one of the transducers of a visual impulse that performs the coupling between rhodopsin and cGMP-phosphodiesterase
Gpm6a	Gpm6a - glycoprotein m6a; Involved in neuronal differentiation, including differentiation and migration of neuronal stem cells. Plays a role in neuronal plasticity and is involved in neurite and filopodia outgrowth, filopodia motility and probably synapse formation. Gpm6a-induced filopodia formation involves mitogen-activated protein kinase (MAPK) and Src signaling pathways.
Gpr153	Gpr153 - G protein-coupled receptor 153; Orphan receptor
Gpx3	Gpx3 - glutathione peroxidase 3; Protects cells and enzymes from oxidative damage, by catalyzing the reduction of hydrogen peroxide, lipid peroxides and organic hydroperoxide, by glutathione
Gsg1l	Gsg1l - GSG1-like; As a component of the inner core of AMPAR complexes, modifies AMPA receptor (AMPAR) gating (By similarity)
Gsn	Gsn - gelsolin; Calcium-regulated, actin-modulating protein that binds to the plus (or barbed) ends of actin monomers or filaments, preventing monomer exchange (end-blocking or capping). It can promote the assembly of monomers into filaments (nucleation) as well as sever filaments already formed. Plays a role in ciliogenesis
Gulp1	Gulp1 - GULP, engulfment adaptor PTB domain containing 1; Modulates cellular glycosphingolipid and cholesterol transport. May play a role in the internalization of various LRP1 ligands, such as PSAP (By similarity). May function as an adapter protein. Required for efficient phagocytosis of apoptotic cells. Increases cellular levels of GTP-bound ARF6
Gypc	Gypc - glycophorin C
H2-Aa	H2-Aa - histocompatibility 2, class II antigen A, alpha
Hacd1	Ptpla - protein tyrosine phosphatase-like (proline instead of catalytic arginine), member a; Responsible for the dehydration step in very long-chain fatty acid (VLCFA) synthesis (By similarity)
Haghl	Haghl - hydroxyacylglutathione hydrolase-like; Hydrolase acting on ester bonds (Potential)
Hao2	Hao2 - hydroxyacid oxidase 2; Has 2-hydroxyacid oxidase activity. Most active on medium-chain substrates
Hba-a1	Hba-a1 - hemoglobin alpha, adult chain 1; Involved in oxygen transport from the lung to the various peripheral tissues (By similarity)
Hba-a2	Hba-a2 - hemoglobin alpha, adult chain 2; Involved in oxygen transport from the lung to the various peripheral tissues (By similarity)
Hbb-bs	Hemoglobin, beta adult s chain
Hbb-bt	Hemoglobin, beta adult t chain
Hhex	Hhex - hematopoietically expressed homeobox; Recognizes the DNA sequence 5'-ATTAA-3' (By similarity). Transcriptional repressor. May play a role in hematopoietic differentiation. Establishes anterior identity at two levels; acts early to enhance canonical WNT-signaling by repressing expression of TLE4, and acts later to inhibit NODAL-signaling by directly targeting NODAL
Hmgcs2	Hmgcs2 - 3-hydroxy-3-methylglutaryl-Coenzyme A synthase 2; This enzyme condenses acetyl-CoA with acetoacetyl-CoA to form HMG-CoA, which is the substrate for HMG-CoA reductase

Hmgn3	Hmgn3 - high mobility group nucleosomal binding domain 3; Binds to nucleosomes, regulating chromatin structure and consequently, chromatin-dependent processes such as transcription, DNA replication and DNA repair. Affects both insulin and glucagon levels and modulates the expression of pancreatic genes involved in insulin secretion. Regulates the expression of the glucose transporter SLC2A2 by binding specifically to its promoter region and recruiting PDX1 and additional transcription factors.
Hoxb2	Hoxb2 - homeobox B2; Sequence-specific transcription factor which is part of a developmental regulatory system that provides cells with specific positional identities on the anterior-posterior axis (By similarity)
Hoxc8	Hoxc8 - homeobox C8; Sequence-specific transcription factor which is part of a developmental regulatory system that provides cells with specific positional identities on the anterior-posterior axis
Hsd11b1	Hsd11b1 - hydroxysteroid 11-beta dehydrogenase 1; Catalyzes reversibly the conversion of cortisol to the inactive metabolite cortisone
Hsd3b1	Hsd3b1 - hydroxy-delta-5-steroid dehydrogenase, 3 beta- and steroid delta-isomerase 1; 3-beta-HSD is a bifunctional enzyme, that catalyzes the oxidative conversion of Delta(5)-ene-3-beta-hydroxy steroid, and the oxidative conversion of ketosteroids. The 3-beta-HSD enzymatic system plays a crucial role in the biosynthesis of all classes of hormonal steroids
Hspa1b	Hspa1b - heat shock protein 1B; In cooperation with other chaperones, Hsp70s stabilize preexistent proteins against aggregation and mediate the folding of newly translated polypeptides in the cytosol as well as within organelles. These chaperones participate in all these processes through their ability to recognize nonnative conformations of other proteins. They bind extended peptide segments with a net hydrophobic character exposed by polypeptides during translation and membrane translocation, or following stress-induced damage (By similarity)
Hspb1	Hspb1 - heat shock protein 1; Involved in stress resistance and actin organization
Hspb8	Hspb8 - heat shock protein 8; Displays temperature-dependent chaperone activity
Htr3a	Htr3a - 5-hydroxytryptamine (serotonin) receptor 3A; This is one of the several different receptors for 5- hydroxytryptamine (serotonin), a biogenic hormone that functions as a neurotransmitter, a hormone, and a mitogen. This receptor is a ligand-gated ion channel, which when activated causes fast, depolarizing responses in neurons. It is a cation-specific, but otherwise relatively nonselective, ion channel
Htra3	Htra3 - HtrA serine peptidase 3; Serine protease that cleaves beta-casein/CSN2 as well as several extracellular matrix (ECM) proteoglycans such as decorin/DCN, biglycan/BGN and fibronectin/FN1. Inhibits signaling mediated by TGF-beta family proteins possibly indirectly by degradation of these ECM proteoglycans. May act as a tumor suppressor. Negatively regulates, in vitro, trophoblast invasion during placental development and may be involved in the development of the placenta in vivo. May also have a role in ovarian development, granulosa cell differentiation and luteinization
Icam2	Icam2 - intercellular adhesion molecule 2; ICAM proteins are ligands for the leukocyte adhesion protein LFA-1 (integrin alpha-L/beta-2). ICAM2 may play a role in lymphocyte recirculation by blocking LFA-1-dependent cell adhesion. It mediates adhesive interactions important for antigen- specific immune response, NK-cell mediated clearance,

	lymphocyte recirculation, and other cellular interactions important for immune response and surveillance
Id2	Id2 - inhibitor of DNA binding 2; ID (inhibitor of DNA binding) HLH proteins lack a basic DNA-binding domain but are able to form heterodimers with other HLH proteins, thereby inhibiting DNA binding. Inhibits skeletal muscle and cardiac myocyte differentiation
Ier2	Ier2 - immediate early response 2
Ier3	Ier3 - immediate early response 3; May play a role in the ERK signaling pathway by inhibiting the dephosphorylation of ERK by phosphatase PP2A- PPP2R5C holoenzyme. Acts also as an ERK downstream effector mediating survival (By similarity). As a member of the NUPR1/RELB/IER3 survival pathway, may allow the development of pancreatic intraepithelial neoplasias
Ifi44	Ifi44 - interferon-induced protein 44; This protein aggregates to form microtubular structures (By similarity)
Igf1	Igf1 - insulin-like growth factor 1; The insulin-like growth factors, isolated from plasma, are structurally and functionally related to insulin but have a much higher growth-promoting activity. May be a physiological regulator of [1-14C]-2-deoxy-D-glucose (2DG) transport and glycogen synthesis in osteoblasts. Stimulates glucose transport in rat bone-derived osteoblastic (PyMS) cells and is effective at much lower concentrations than insulin, not only regarding glycogen and DNA synthesis but also with regard to enhancing glucose uptake (By similarity)
Igfbp4	Igfbp4 - insulin-like growth factor binding protein 4; IGF-binding proteins prolong the half-life of the IGFs and have been shown to either inhibit or stimulate the growth promoting effects of the IGFs on cell culture. They alter the interaction of IGFs with their cell surface receptors
Igha	Immunoglobulin heavy constant alpha
Igkc	Immunoglobulin kappa constant
Il23r	Il23r - interleukin 23 receptor; Associates with IL12RB1 to form the interleukin-23 receptor. Binds IL23 and mediates T-cells, NK cells and possibly certain macrophage/myeloid cells stimulation probably through activation of the Jak-Stat signaling cascade. IL23 functions in innate and adaptive immunity and may participate in acute response to infection in peripheral tissues. IL23 may be responsible for autoimmune inflammatory diseases and be important for tumorigenesis (By similarity)
Il2rg	Il2rg - interleukin 2 receptor, gamma chain; Common subunit for the receptors for a variety of interleukins
Impdh2	Impdh2 - inosine 5'-phosphate dehydrogenase 2; Catalyzes the conversion of inosine 5'-phosphate (IMP) to xanthosine 5'-phosphate (XMP), the first committed and rate- limiting step in the de novo synthesis of guanine nucleotides, and therefore plays an important role in the regulation of cell growth. Could also have a single-stranded nucleic acid-binding activity and could play a role in RNA and/or DNA metabolism (By similarity). It may also have a role in the development of malignancy and the growth progression of some tumors
Insig1	Insig1 - insulin induced gene 1; Mediates feedback control of cholesterol synthesis by controlling SCAP and HMGCR. Functions by blocking the processing of sterol regulatory element-binding proteins (SREBPs). Capable of retaining the SCAP-SREBF2 complex in the ER thus preventing it from escorting SREBPs to the Golgi. Initiates the sterol-

	mediated ubiquitin-mediated endoplasmic reticulum-associated degradation (ERAD) of HMGCR via recruitment of the reductase to the ubiquitin ligase, AMFR/gp78. May play a role in growth and differentiation of tissues involved in metabolic control.
Ipp	Ipp - IAP promoted placental gene; May play a role in organizing the actin cytoskeleton
Irs2	Irs2 - insulin receptor substrate 2; May mediate the control of various cellular processes by insulin
Isg20	Isg20 - interferon-stimulated protein; Exonuclease with specificity for single-stranded RNA and, to a lesser extent for DNA. Degrades RNA at a rate that is approximately 35-fold higher than its rate for single-stranded DNA. May be involved in the antiviral function of IFN against RNA viruses (By similarity)
Ism1	Ism1 - isthmin 1 homolog (zebrafish)
Itga1	Itga1 - integrin alpha 1; Integrin alpha-1/beta-1 is a receptor for laminin and collagen. It recognizes the proline-hydroxylated sequence G-F-P-G-E-R in collagen (By similarity)
Izumo4	Izumo4 - IZUMO family member 4
Junb	Junb - Jun-B oncogene; Transcription factor involved in regulating gene activity following the primary growth factor response. Binds to the DNA sequence 5'-TGA[CG]TCA-3'
Kcne4	Kcne4 - potassium voltage-gated channel, Isk-related subfamily, gene 4; Ancillary protein that assembles as a beta subunit with a voltage-gated potassium channel complex of pore-forming alpha subunits. Modulates the gating kinetics and enhances stability of the channel complex. Associated with KCNQ1/KVLTQ1 inhibits potassium current (By similarity)
Kcnn2	Kcnn2 - potassium intermediate/small conductance calcium-activated channel, subfamily N, member 2; Forms a voltage-independent potassium channel activated by intracellular calcium. Activation is followed by membrane hyperpolarization. Thought to regulate neuronal excitability by contributing to the slow component of synaptic afterhyperpolarization. The channel is blocked by apamin (By similarity)
Kdm6b	Kdm6b - KDM1 lysine (K)-specific demethylase 6B; Histone demethylase that specifically demethylates 'Lys- 27' of histone H3, thereby playing a central role in histone code. Demethylates trimethylated and dimethylated H3 'Lys-27'. Plays a central role in regulation of posterior development, by regulating HOX gene expression. Involved in inflammatory response by participating in macrophage differentiation in case of inflammation by regulating gene expression and macrophage differentiation
Klf4	Klf4 - Kruppel-like factor 4 (gut); Transcription factor; can act both as activator and as repressor. Binds the 5'-CACCC-3' core sequence. Binds to the promoter region of its own gene and can activate its own transcription. Regulates the expression of key transcription factors during embryonic development. Plays an important role in maintaining embryonic stem cells, and in preventing their differentiation. Required for establishing the barrier function of the skin and for postnatal maturation and maintenance of the ocular surface
Klf5	Klf5 - Kruppel-like factor 5; Transcription factor that binds to GC box promoter elements. Activates the transcription of these genes
Klhl14	Klhl14 - kelch-like 14 (Drosophila)

Lama4	Lama4 - laminin, alpha 4; Binding to cells via a high affinity receptor, laminin is thought to mediate the attachment, migration and organization of cells into tissues during embryonic development by interacting with other extracellular matrix components
Lefty1	Lefty1 - left right determination factor 1; Required for left-right axis determination as a regulator of LEFTY2 and NODAL
Lilr4b	Leukocyte immunoglobulin-like receptor, subfamily B, member 4B
Lipa	Lipa - lysosomal acid lipase A; Crucial for the intracellular hydrolysis of cholesteryl esters and triglycerides that have been internalized via receptor- mediated endocytosis of lipoprotein particles. Important in mediating the effect of LDL (low density lipoprotein) uptake on suppression of hydroxymethylglutaryl-CoA reductase and activation of endogenous cellular cholesteryl ester formation (By similarity)
Lmcd1	Lmcd1 - LIM and cysteine-rich domains 1; Transcriptional cofactor that restricts GATA6 function by inhibiting DNA-binding, resulting in repression of GATA6 transcriptional activation of downstream target genes. Represses GATA6-mediated trans activation of lung- and cardiac tissue- specific promoters. Inhibits DNA-binding by GATA4 and GATA1 to the cTNC promoter. Plays a critical role in the development of cardiac hypertrophy via activation of calcineurin/nuclear factor of activated T-cells signaling pathway
Lmln	Lmln - leishmanolysin-like (metallopeptidase M8 family); Metalloprotease essential for the coordination of mitotic progression, and also plays a role in cell migration (By similarity)
Lnpep	Lnpep - leucyl/cystinyl aminopeptidase; Release of an N-terminal amino acid, cleave before cysteine, leucine as well as other amino acids. Degrades peptide hormones such as oxytocin, vasopressin and angiotensin III, and plays a role in maintaining homeostasis during pregnancy. May be involved in the inactivation of neuronal peptides in the brain. Cleaves Met-enkephalin and dynorphin. Binds angiotensin IV and may be the angiotensin IV receptor in the brain (By similarity)
Loxl1	Loxl1 - lysyl oxidase-like 1; Active on elastin and collagen substrates (By similarity)
Lpin3	Lpin3 - lipin 3; Regulates fatty acid metabolism. Magnesium-dependent phosphatidate phosphatase enzyme which catalyzes the conversion of phosphatidic acid to diacylglycerol during triglyceride, phosphatidylcholine and phosphatidylethanolamine biosynthesis
Lrrc32	Lrrc32 - leucine rich repeat containing 32
Lsp1	Lsp1 - lymphocyte specific 1
Lum	Lum - lumican
Ly96	Ly96 - lymphocyte antigen 96; Cooperates with TLR4 in the innate immune response to bacterial lipopolysaccharide (LPS), and with TLR2 in the response to cell wall components from Gram-positive and Gram-negative bacteria. Enhances TLR4-dependent activation of NF-kappa-B. Cells expressing both MD2 and TLR4, but not TLR4 alone, respond to LPS (By similarity)
Malat1	Metastasis associated lung adenocarcinoma transcript 1
Man1a	Man1a - mannosidase 1, alpha; Involved in the maturation of Asn-linked oligosaccharides. Progressively trim alpha-1,2-linked mannose residues from Man(9)GlcNAc(2) to produce Man(5)GlcNAc(2)
Mast4	Mast4 - microtubule associated serine/threonine kinase family member 4

Mbip	Mbip - MAP3K12 binding inhibitory protein 1; Inhibits the MAP3K12 activity to induce the activation of the JNK/SAPK pathway (By similarity). Component of the ATAC complex, a complex with histone acetyltransferase activity on histones H3 and H4 (By similarity)
Mc2r	Mc2r - melanocortin 2 receptor; Receptor for ACTH. This receptor is mediated by G proteins (G(s)) which activate adenylate cyclase
Mcam	Mcam - melanoma cell adhesion molecule; Plays a role in cell adhesion, and in cohesion of the endothelial monolayer at intercellular junctions in vascular tissue. Its expression may allow melanoma cells to interact with cellular elements of the vascular system, thereby enhancing hematogeneous tumor spread. Could be an adhesion molecule active in neural crest cells during embryonic development. Acts as surface receptor that triggers tyrosine phosphorylation of FYN and PTK2/FAK1, and a transient increase in the intracellular calcium concentration (By similarity)
Metrnl	Metrnl - meteorin, glial cell differentiation regulator-like
Mfap4	Mfap4 - microfibrillar-associated protein 4; Could be involved in calcium-dependent cell adhesion or intercellular interactions (By similarity)
Mgmt	Mgmt - O-6-methylguanine-DNA methyltransferase; Involved in the cellular defense against the biological effects of O6-methylguanine (O6-MeG) in DNA. Repairs alkylated guanine in DNA by stoichiometrically transferring the alkyl group at the O-6 position to a cysteine residue in the enzyme. This is a suicide reaction: the enzyme is irreversibly inactivated
Mgp	Mgp - matrix Gla protein; Associates with the organic matrix of bone and cartilage. Thought to act as an inhibitor of bone formation
Mid1	Mid1 - midline 1; Has E3 ubiquitin ligase activity towards IGBP1, promoting its monoubiquitination, which results in deprotection of the catalytic subunit of protein phosphatase PP2A, and its subsequent degradation by polyubiquitination (By similarity)
Midn	Midn - midnolin; May be involved in regulation of genes related to neurogenesis in the nucleolus
Mknk2	Mknk2 - MAP kinase-interacting serine/threonine kinase 2; Serine/threonine-protein kinase that phosphorylates SFPQ/PSF, HNRNPA1 and EIF4E. May play a role in the response to environmental stress and cytokines. Appears to regulate translation by phosphorylating EIF4E, thus increasing the affinity of this protein for the 7-methylguanosine-containing mRNA cap. Required for mediating PP2A-inhibition-induced EIF4E phosphorylation. Triggers EIF4E shuttling from cytoplasm to nucleus. Enhances the formation of EIF4F complex in pachytene spermatocytes, thus promoting mRNA translation during spermatogenesis
Mmp2	Mmp2 - matrix metallopeptidase 2; Ubiquitinous metalloproteinase that is involved in diverse functions such as remodeling of the vasculature, angiogenesis, tissue repair, tumor invasion, inflammation, and atherosclerotic plaque rupture. As well as degrading extracellular matrix proteins, can also act on several nonmatrix proteins such as big endothelial 1 and beta-type CGRP promoting vasoconstriction. Also cleaves KISS at a Gly-|-Leu bond. Appears to have a role in myocardial cell death pathways. Contributes to myocardial oxidative stress by regulating the activity of GSK3beta.
Mphosph6	Mphosph6 - M phase phosphoprotein 6; RNA-binding protein that associates with the RNA exosome complex. Involved in the 3'processing of the 7S pre-RNA to the mature 5.8S rRNA and may play a role in

	recruiting the RNA exosome complex to pre-rRNA; this function may include C1D (By similarity)
Mpp7	Mpp7 - membrane protein, palmitoylated 7 (MAGUK p55 subfamily member 7); Acts as an important adapter that promotes epithelial cell polarity and tight junction formation via its interaction with DLG1. Involved in the assembly of protein complexes at sites of cell-cell contact (By similarity)
Mpzl2	Mpzl2 - myelin protein zero-like 2; Mediates homophilic cell-cell adhesion
Mrvi1	Mrvi1 - MRV integration site 1; Plays a role as NO/PRKG1-dependent regulator of IP3- induced calcium release; its phosphorylation by PRKG1 inhibits bradykinin and IP3-induced calcium release from intracellular stores. Recruits PRKG1 to the endoplasmic reticulum and may mediate the assembly of PRKG1 and ITPR1 in a macrocomplex. Involved in PRKG1 signaling cascade leading to inhibition of platelet activation and aggregation. Mediates also NO-dependent inhibition of calcium signaling in gastrointestinal smooth muscle contributing to NO-dependent relaxation
Ms4a7	Ms4a7 - membrane-spanning 4-domains, subfamily A, member 7
Myl9	Myl9 - myosin, light polypeptide 9, regulatory; Myosin regulatory subunit that plays an important role in regulation of both smooth muscle and nonmuscle cell contractile activity via its phosphorylation. Implicated in cytokinesis, receptor capping, and cell locomotion (By similarity)
Nans	Nans - N-acetylneuraminic acid synthase (sialic acid synthase)
Napepld	Napepld - N-acyl phosphatidylethanolamine phospholipase D; Hydrolyzes N-acyl-phosphatidylethanolamines (NAPEs) to produce N-acylethanolamines (NAEs) and phosphatidic acid. Responsible for the generation of anandamide (N- arachidonoylethanolamine), the ligand of cannabinoid and vanilloid receptors
Nat8	Nat8 - N-acetyltransferase 8 (GCN5-related, putative); Plays a role in regulation of gastrulation
Nek5	Nek5 - NIMA (never in mitosis gene a)-related expressed kinase 5
Nnt	Nnt - nicotinamide nucleotide transhydrogenase; The transhydrogenation between NADH and NADP is coupled to respiration and ATP hydrolysis and functions as a proton pump across the membrane. May play a role in reactive oxygen species (ROS) detoxification in the adrenal gland (By similarity)
Nr1h4	Nr1h4 - nuclear receptor subfamily 1, group H, member 4; Ligand-activated transcription factor. Receptor for bile acids such as chenodeoxycholic acid, lithocholic acid and deoxycholic acid. Represses the transcription of the cholesterol 7-alpha-hydroxylase gene (CYP7A1) and activates the intestinal bile acid-binding protein (IBABP). Activates the transcription of bile salt export pump ABCB11 by directly recruiting histone methyltransferase CARM1 within its gene locus (By similarity)
Nr4a2	Nr4a2 - nuclear receptor subfamily 4, group A, member 2; Transcriptional regulator which is important for the differentiation and maintenance of meso-diencephalic dopaminergic (mdDA) neurons during development. It is crucial for expression of a set of genes such as SLC6A3, SLC18A2, TH and DRD2 which are essential for development of mdDA neurons

Ntrk2	Ntrk2 - neurotrophic tyrosine kinase, receptor, type 2; Receptor tyrosine kinase involved in the development and the maturation of the central and the peripheral nervous systems through regulation of neuron survival, proliferation, migration, differentiation, and synapse formation and plasticity. Receptor for BDNF/brain-derived neurotrophic factor and NTF4/neurotrophin- 4. Alternatively can also bind NTF3/neurotrophin-3 which is less efficient in activating the receptor but regulates neuron survival through NTRK2
Nudt16	Nudt16 - nudix (nucleoside diphosphate linked moiety X)-type motif 16; RNA-binding and decapping enzyme that catalyzes the cleavage of the cap structure of snoRNAs and mRNAs in a metal- dependent manner. Part of the U8 snoRNP complex that is required for the accumulation of mature 5.8S and 28S rRNA. Has diphosphatase activity and removes m7G and/or m227G caps from U8 snoRNA and leaves a 5'monophosphate on the RNA. Catalyzes also the cleavage of the cap structure on mRNAs. Does not hydrolyze cap analog structures like 7-methylguanosine nucleoside triphosphate (m7GpppG).
Nufip1	Nufip1 - nuclear fragile X mental retardation protein interacting protein 1; Binds RNA
Nup35	Nup35 - nucleoporin 35; Functions as a component of the nuclear pore complex (NPC). NPC components, collectively referred to as nucleoporins (NUPs), can play the role of both NPC structural components and of docking or interaction partners for transiently associated nuclear transport factors. May play a role in the association of MAD1 with the NPC (By similarity)
Ogn	Ogn - osteoglycin; Induces bone formation in conjunction with TGF-beta-1 or TGF-beta-2 (By similarity)
Olfml1	Olfml1 - olfactomedin-like 1
Omd	Omd - osteomodulin; May be implicated in biomineralization processes. Has a function in binding of osteoblasts via the alpha(V)beta(3)- integrin (By similarity)
Orm3	Orm3 - orosomucoid 3; Functions as transport protein in the blood stream. Binds various ligands in the interior of its beta-barrel domain (By similarity). Appears to function in modulating the activity of the immune system during the acute-phase reaction
Ormdl1	Ormdl1 - ORM1-like 1 (S. cerevisiae); Negative regulator of sphingolipid synthesis (By similarity)
Osgin1	Osgin1 - oxidative stress induced growth inhibitor 1
Oxr1	Oxr1 - oxidation resistance 1; May be involved in protection from oxidative damage
Pacsin3	Pacsin3 - protein kinase C and casein kinase substrate in neurons 3; May play a role in endocytosis
Paip1	Paip1 - polyadenylate binding protein-interacting protein 1; Acts as a coactivator in the regulation of translation initiation of poly(A)-containing mRNAs. Its stimulatory activity on translation is mediated via its action on PABPC1. Competes with PAIP2 for binding to PABPC1. Its association with EIF4A and PABPC1 may potentiate contacts between mRNA termini. May also be involved in translationally coupled mRNA turnover. Implicated with other RNA-binding proteins in the cytoplasmic deadenylation/translational and decay interplay of the FOS mRNA mediated by the major coding-region determinant
Paqr7	Paqr7 - progestin and adipoQ receptor family member VII; Steroid membrane receptor. May be involved in oocyte maturation (By similarity)

Parp16	Parp16 - poly (ADP-ribose) polymerase family, member 16; Mono-ADP-ribosyltransferase targeting the karyopherin KPNB1. Plays a role in unfolded protein response (UPR), by ADP- ribosylating and activating EIF2AK3 and ERN1, two important UPR effectors (By similarity)
Pcsk6	Pcsk6 - proprotein convertase subtilisin/kexin type 6
Pdgfc	Pdgfc - platelet-derived growth factor, C polypeptide; Growth factor that plays an essential role in the regulation of embryonic development, cell proliferation, cell migration, survival and chemotaxis. Potent mitogen and chemoattractant for cells of mesenchymal origin. Required for normal skeleton formation during embryonic development, especially for normal development of the craniofacial skeleton and for normal development of the palate. Required for normal skin morphogenesis during embryonic development. Plays an important role in wound healing
Pdgfd	Pdgfd - platelet-derived growth factor, D polypeptide; Growth factor that plays an essential role in the regulation of embryonic development, cell proliferation, cell migration, survival and chemotaxis. Potent mitogen for cells of mesenchymal origin. Plays an important role in wound healing (By similarity). Has oncogenic potential and can induce tumor formation. Induces macrophage recruitment, increased interstitial pressure, and blood vessel maturation during angiogenesis. Can initiate events that lead to a mesangial proliferative glomerulonephritis, including influx of monocytes and macrophages
Pdgfra	Pdgfra - platelet derived growth factor receptor, alpha polypeptide; Tyrosine-protein kinase that acts as a cell-surface receptor for PDGFA, PDGFB and PDGFC and plays an essential role in the regulation of embryonic development, cell proliferation, survival and chemotaxis. Depending on the context, promotes or inhibits cell proliferation and cell migration. Plays an important role in the differentiation of bone marrow-derived mesenchymal stem cells. Required for normal skeleton development and cephalic closure during embryonic development.
Pdlim2	Pdlim2 - PDZ and LIM domain 2; Probable adapter protein located at the actin cytoskeleton that promotes cell attachment. Necessary for the migratory capacity of epithelial cells. Overexpression enhances cell adhesion to collagen and fibronectin and suppresses anchorage independent growth. May contribute to tumor cell migratory capacity (By similarity)
Pdlim4	Pdlim4 - PDZ and LIM domain 4
Penk	Penk - preproenkephalin; Met- and Leu-enkephalins compete with and mimic the effects of opiate drugs. They play a role in a number of physiologic functions, including pain perception and responses to stress. PENK(114-133) and PENK(238-259) increase glutamate release in the striatum. PENK(114-133) decreases GABA concentration in the striatum
Pf4	Pf4 - platelet factor 4; Released during platelet aggregation. Neutralizes the anticoagulant effect of heparin because it binds more strongly to heparin than to the chondroitin-4-sulfate chains of the carrier molecule. Chemotactic for neutrophils and monocytes. Inhibits endothelial cell proliferation (By similarity)
Pfkfb2	Pfkfb2 - 6-phosphofructo-2-kinase/fructose-2,6-biphosphatase 2; Synthesis and degradation of fructose 2,6-bisphosphate
Pgm5	Pgm5 - phosphoglucomutase 5; Component of adherens-type cell-cell and cell-matrix junctions. Lacks phosphoglucomutase activity (By similarity)
Pianp	Pianp - PILR alpha associated neural protein; Acts as a ligand for PILRA

	in neuronal tissues, where it may be involved in immune regulation
Pigh	Pigh - phosphatidylinositol glycan anchor biosynthesis, class H; Part of the complex catalyzing the transfer of N- acetylglucosamine from UDP-N-acetylglucosamine to phosphatidylinositol, the first step of GPI biosynthesis (By similarity)
Pik3ap1	Pik3ap1 - phosphoinositide-3-kinase adaptor protein 1; Signaling adapter that contributes to B-cell development by linking B-cell receptor (BCR) signaling to the phosphoinositide 3-kinase (PI3K)-Akt signaling pathway. Has a complementary role to the BCR coreceptor CD19, coupling BCR and PI3K activation by providing a docking site for the PI3K subunit PIK3R1. Alternatively, links Toll-like receptor (TLR) signaling to PI3K activation, a process preventing excessive inflammatory cytokine production. Also involved in the activation of PI3K in natural killer cells.
Pim3	Pim3 - proviral integration site 3; Proto-oncogene with serine/threonine kinase activity that can prevent apoptosis and promote cell survival and protein translation. May contribute to tumorigenesis through: the delivery of survival signaling through phosphorylation of BAD which induces release of the anti-apoptotic protein Bcl-X(L), the regulation of cell cycle progression and protein synthesis and by regulation of MYC transcriptional activity. Additionally to this role on tumorigenesis, can also negatively regulate insulin secretion by inhibiting the activation of MAPK1/3 (ERK1/2)
Pkia	Pkia - protein kinase inhibitor, alpha; Extremely potent competitive inhibitor of cAMP-dependent protein kinase activity, this protein interacts with the catalytic subunit of the enzyme after the cAMP-induced dissociation of its regulatory chains
Plac9a	Plac9a - Placenta specific 9a
Plau	Plau - plasminogen activator, urokinase; Specifically cleaves the zymogen plasminogen to form the active enzyme plasmin
Plxnb1	Plxnb1 - plexin B1; Receptor for SEMA4D. Plays a role in RHOA activation and subsequent changes of the actin cytoskeleton. Plays a role in axon guidance, invasive growth and cell migration
Pmvk	Pmvk - phosphomevalonate kinase
Pnrc2	Pnrc2 - proline-rich nuclear receptor coactivator 2; Involved in nonsense-mediated mRNA decay (NMD) by acting as a bridge between the mRNA decapping complex and the NMD machinery. May act by targeting the NMD machinery to the P-body and recruiting the decapping machinery to aberrant mRNAs. Required for UPF1/RENT1 localization to the P-body. Also acts as a nuclear receptor coactivator. May play a role in controlling the energy balance between energy storage and energy expenditure (By similarity)
Pop7	Pop7 - processing of precursor 7, ribonuclease P family, (S. cerevisiae); Component of ribonuclease P, a protein complex that generates mature tRNA molecules by cleaving their 5'-ends (By similarity)
Ppcs	Ppcs - phosphopantothenoylcysteine synthetase; Catalyzes the first step in the biosynthesis of coenzyme A from vitamin B5, where cysteine is conjugated to 4'- phosphopantothenate to form 4-phosphopantothenoylcysteine (By similarity)
Ppl	Ppl - periplakin; Component of the cornified envelope of keratinocytes. May link the cornified envelope to desmosomes and intermediate filaments. May act as a localization signal in PKB/AKT-mediated signaling

Ppp1r15a	Ppp1r15a - protein phosphatase 1, regulatory (inhibitor) subunit 15A; Recruits the serine/threonine-protein phosphatase PP1 to dephosphorylate the translation initiation factor eIF-2A/EIF2S1, thereby reversing the shut-off of protein synthesis initiated by stress-inducible kinases and facilitating recovery of cells from stress. Down-regulates the TGF-beta signaling pathway by promoting dephosphorylation of TGFB1 by PP1. May promote apoptosis by inducing TP53 phosphorylation on 'Ser-15'. In case of infection with vesicular stomatitis virus (VSV), impairs viral replication
Prr5	Prr5 - proline rich 5 (renal); Subunit of mTORC2, which regulates cell growth and survival in response to hormonal signals. mTORC2 is activated by growth factors, but, in contrast to mTORC1, seems to be nutrient- insensitive. mTORC2 seems to function upstream of Rho GTPases to regulate the actin cytoskeleton, probably by activating one or more Rho-type guanine nucleotide exchange factors. mTORC2 promotes the serum-induced formation of stress-fibers or F-actin.
Prrg3	Prrg3 - proline rich Gla (G-carboxyglutamic acid) 3 (transmembrane)
Prrg4	Prrg4 - proline rich Gla (G-carboxyglutamic acid) 4 (transmembrane)
Prss23	Prss23 - protease, serine, 23
Prss35	Prss35 - protease, serine, 35
Ptger3	Ptger3 - prostaglandin E receptor 3 (subtype EP3); Receptor for prostaglandin E2 (PGE2); the EP3 receptor may be involved in inhibition of gastric acid secretion, modulation of neurotransmitter release in central and peripheral neurons, inhibition of sodium and water reabsorption in kidney tubulus and contraction in uterine smooth muscle. The activity of this receptor can couple to both the inhibition of adenylate cyclase mediated by G(i) proteins, and to an elevation of intracellular calcium. The various forms can interact with different second messenger systems
Ptges	Ptges - prostaglandin E synthase; Catalyzes the oxidoreduction of prostaglandin endoperoxide H2 (PGH2) to prostaglandin E2 (PGE2) (By similarity)
Pth1r	Pth1r - parathyroid hormone 1 receptor; This is a receptor for parathyroid hormone and for parathyroid hormone-related peptide. The activity of this receptor is mediated by G proteins which activate adenylyl cyclase and also a phosphatidylinositol-calcium second messenger system
Ptpn4	Ptpn4 - protein tyrosine phosphatase, non-receptor type 4; May act at junctions between the membrane and the cytoskeleton (By similarity)
Pyroxd2	Pyroxd2 - pyridine nucleotide-disulphide oxidoreductase domain 2; Probable oxidoreductase (By similarity)
Rabep1	Rabep1 - rabaptin, RAB GTPase binding effector protein 1; Rab effector protein acting as linker between gamma- adaptin, RAB4A and RAB5A. Involved in endocytic membrane fusion and membrane trafficking of recycling endosomes. Stimulates RABGEF1 mediated nucleotide exchange on RAB5A (By similarity)
Rabep2	Rabep2 - rabaptin, RAB GTPase binding effector protein 2; Plays a role in membrane trafficking and in homotypic early endosome fusion (By similarity)
Rangrf	Rangrf - RAN guanine nucleotide release factor; May regulate the intracellular trafficking of RAN. In cardiac cells seems to regulate the cell surface localization of SCN5A
Raph1	Raph1 - Ras association (RalGDS/AF-6) and pleckstrin homology

	domains 1
Rarres2	Rarres2 - retinoic acid receptor responder (tazarotene induced) 2
Rbp1	Rbp1 - retinol binding protein 1, cellular; Intracellular transport of retinol
Rgma	Rgma - RGM domain family, member A; Member of the repulsive guidance molecule (RGM) family that performs several functions in the developing and adult nervous system. Regulates cephalic neural tube closure, inhibits neurite outgrowth and cortical neuron branching, and the formation of mature synapses. Binding to its receptor NEO1/neogenin induces activation of RHOA-ROCK1/Rho-kinase signaling pathway through UNC5B-ARHGEF12/LARG-PTK2/FAK1 cascade, leading to collapse of the neuronal growth cone and neurite outgrowth inhibition.
Rmdn2	Fam82a1 - family with sequence similarity 82, member A1
Rmrp	RNA component of mitochondrial RNA processing endoribonuclease
Rnase4	Rnase4 - ribonuclease, RNase A family 4; This RNase has marked specificity towards the 3' side of uridine nucleotides (By similarity)
RP23-338P12.2	RIKEN cDNA 7330423F06
RP23-371A16.3	RIKEN cDNA 4932422M17 gene
RP24-230J14.5	Predicted gene 42427
RP24-378G4.3	predicted gene 43305, known TEC
Rpl28-ps1	Ribosomal protein L28, pseudogene 1
Rpp38	Rpp38 - ribonuclease P/MRP 38 subunit (human); Component of ribonuclease P, a protein complex that generates mature tRNA molecules by cleaving their 5'-ends. RPP38 may associate transiently with RNase P RNA as a factor involved in the transport of H1 RNA to the putative site of its assembly in the cell, the nucleolus (By similarity)
Rps4l	Ribosomal protein S4-like
Rspo1	Rspo1 - R-spondin homolog (Xenopus laevis); Activator of the beta-catenin signaling cascade, leading to TCF-dependent gene activation. Acts both in the canonical Wnt/beta-catenin-dependent pathway and in non-canonical Wnt signaling pathway, probably by acting as an inhibitor of ZNRF3, an important regulator of the Wnt signaling pathway. Acts as a ligand for frizzled FZD8 and LRP6. May negatively regulate the TGF-beta pathway. Has essential roles in ovary determination (By similarity)
Rtl1	Rtl1 - retrotransposon-like 1; Plays an essential role in capillaries endothelial cells for the maintenance of feto-maternal interface and for development of the placenta
S100a11	S100a11 - S100 calcium binding protein A11 (calgizzarin); Facilitates the differentiation and the cornification of keratinocytes (By similarity)
S100a6	S100a6 - S100 calcium binding protein A6 (calcyclin); May function as calcium sensor and contribute to cellular calcium signaling (Potential). May function by interacting with other proteins and indirectly play a role in the reorganization of the actin cytoskeleton and in cell motility. Binds 2 calcium ions. Calcium binding is cooperative (By similarity). Interacts with FKBP4 (By similarity)
S1pr2	S1pr2 - sphingosine-1-phosphate receptor 2; Receptor for the lysosphingolipid sphingosine 1- phosphate (S1P). S1P is a bioactive lysophospholipid that elicits diverse physiological effect on most types of cells and tissues
Samd9l	Samd9l - sterile alpha motif domain containing 9-like
Sardh	Sardh - sarcosine dehydrogenase

Scara5	Scara5 - scavenger receptor class A, member 5 (putative); Ferritin receptor that mediates non-transferrin- dependent delivery of iron. Mediates cellular uptake of ferritin- bound iron by stimulating ferritin endocytosis from the cell surface with consequent iron delivery within the cell. Delivery of iron to cells by ferritin is required for the development of specific cell types, suggesting the existence of cell type-specific mechanisms of iron traffic in organogenesis, which alternatively utilize transferrin or non-transferrin iron delivery pathways.
Scx	Scx - scleraxis; Plays an early essential role in mesoderm formation, as well as a later role in formation of somite-derived chondrogenic lineages
Sdc1	Sdc1 - syndecan 1; Cell surface proteoglycan that bears both heparan sulfate and chondroitin sulfate and that links the cytoskeleton to the interstitial matrix
Sema5b	Sema5b - sema domain, seven thrombospondin repeats (type 1 and type 1-like), transmembrane domain (TM) and short cytoplasmic domain, (semaphorin) 5B; May act as positive axonal guidance cues
Serpinb6b	Serpinb6b - serine (or cysteine) peptidase inhibitor, clade B, member 6b
Serpinf1	Serpinf1 - serine (or cysteine) peptidase inhibitor, clade F, member 1; Neurotrophic protein; induces extensive neuronal differentiation in retinoblastoma cells. Potent inhibitor of angiogenesis. As it does not undergo the S (stressed) to R (relaxed) conformational transition characteristic of active serpins, it exhibits no serine protease inhibitory activity
Serpinh1	Serpinh1 - serine (or cysteine) peptidase inhibitor, clade H, member 1; Binds specifically to collagen. Could be involved as a chaperone in the biosynthetic pathway of collagen
Sfrp5	Sfrp5 - secreted frizzled-related sequence protein 5; Soluble frizzled-related proteins (sFRPS) function as modulators of Wnt signaling through direct interaction with Wnts. They have a role in regulating cell growth and differentiation in specific cell types. SFRP5 may be involved in determining the polarity of photoreceptor, and perhaps, other cells in the retina
Sh3pxd2a	Sh3pxd2a - SH3 and PX domains 2A; Adapter protein involved in invadopodia and podosome formation, extracellular matrix degradation and invasiveness of some cancer cells. Binds matrix metalloproteinases (ADAMs), NADPH oxidases (NOXs) and phosphoinositides. Acts as an organizer protein that allows NOX1- or NOX3-dependent reactive oxygen species (ROS) generation and ROS localization. In association with ADAM12, mediates the neurotoxic effect of beta-amyloid peptide (By similarity)
Sik2	Sik2 - salt inducible kinase 2; Phosphorylates 'Ser-789' of IRS1 in insulin-stimulated adipocytes, potentially modulating the efficiency of insulin signal transduction. Inhibits CREB activity by phosphorylating and repressing TORCs, the CREB-specific coactivators
Ska2	Ska2 - spindle and kinetochore associated complex subunit 2; Component of the SKA1 complex, a microtubule-binding subcomplex of the outer kinetochore that is essential for proper chromosome segregation. Required for timely anaphase onset during mitosis, when chromosomes undergo bipolar attachment on spindle microtubules leading to silencing of the spindle checkpoint. The SKA1 complex is a direct component of the kinetochore-microtubule interface and directly associates with microtubules as oligomeric assemblies.
Slamf9	Slamf9 - SLAM family member 9; May play a role in the immune response (By similarity)

Slc18b1	Slc18b1 - RIKEN cDNA 1110021L09 gene
Slc2a4rg-ps	Slc2a4 regulator, pseudogene
Slc35b3	Slc35b3 - solute carrier family 35, member B3; Mediates the transport of adenosine 3'-phospho 5'- phosphosulfate (PAPS), from cytosol into Golgi. PAPS is a universal sulfuryl donor for sulfation events that take place in the Golgi. Compensates for the insufficient expression of SLC35B2/PAPST1 during the synthesis of sulfated glycoconjugates in the colon (By similarity)
Slc45a3	Slc45a3 - solute carrier family 45, member 3
Slc6a2	Slc6a2 - solute carrier family 6 (neurotransmitter transporter, noradrenalin), member 2; Amine transporter. Terminates the action of noradrenaline by its high affinity sodium-dependent reuptake into presynaptic terminals
Sncg	Sncg - synuclein, gamma; Plays a role in neurofilament network integrity. May be involved in modulating axonal architecture during development and in the adult. In vitro, increases the susceptibility of neurofilament-H to calcium-dependent proteases. May also function in modulating the keratin network in skin. Activates the MAPK and Elk-1 signal transduction pathway (By similarity)
Snhg4	Small nucleolar RNA host gene 4
Snupn	Snupn - snurportin 1; Functions as an U snRNP-specific nuclear import adapter. Involved in the trimethylguanosine (m3G)-cap-dependent nuclear import of U snRNPs. Binds specifically to the terminal m3G-cap U snRNAs (By similarity)
Snx32	Snx32 - sorting nexin 32; May be involved in several stages of intracellular trafficking (By similarity)
Sorcs2	Sorcs2 - sortilin-related VPS10 domain containing receptor 2
Sox12	Sox12 - SRY-box containing gene 12; Binds to the sequence 5'-AACAAT-3' (By similarity)
Sparcl1	Sparcl1 - SPARC-like 1
Spp1	Spp1 - secreted phosphoprotein 1; Binds tightly to hydroxyapatite. Appears to form an integral part of the mineralized matrix. Probably important to cell-matrix interaction
Spry2	Spry2 - sprouty homolog 2 (Drosophila); May function as an antagonist of fibroblast growth factor (FGF) pathways and may negatively modulate respiratory organogenesis
Srp54a	Srp54a - signal recognition particle 54A
Srpx	Srpx - sushi-repeat-containing protein
Srpx2	Srpx2 - sushi-repeat-containing protein, X-linked 2; Acts as a ligand for the urokinase plasminogen activator surface receptor. Plays a role in angiogenesis by inducing endothelial cell migration and the formation of vascular network (cords). Involved in cellular migration and adhesion in cancer cells. Increases the phosphorylation levels of FAK
Steap1	Steap1 - six transmembrane epithelial antigen of the prostate 1; Metalloreductase that has the ability to reduce both $Fe(3+)$ to $Fe(2+)$ and $Cu(2+)$ to $Cu(1+)$. Uses NAD(+) as acceptor
Steap2	Steap2 - six transmembrane epithelial antigen of prostate 2; Metalloreductase that has the ability to reduce both $Fe(3+)$ to $Fe(2+)$ and $Cu(2+)$ to $Cu(1+)$. Uses NAD(+) as acceptor
Susd3	Susd3 - sushi domain containing 3
Syne4	AI428936 - expressed sequence AI428936; Contributes to the establishment of secretory epithelial morphology, by promoting kinesin-

	dependent apical migration of the centrosome and Golgi apparatus and basal localization of the nucleus
Tagln	Tagln - transgelin; Actin cross-linking/gelling protein (By similarity)
Tbx3os1	T-box 3, opposite strand 1
Tfpi	Tfpi - tissue factor pathway inhibitor; Inhibits factor X (X(a)) directly and, in a Xa-dependent way, inhibits VIIa/tissue factor activity, presumably by forming a quaternary Xa/LACI/VIIa/TF complex. It possesses an antithrombotic action and also the ability to associate with lipoproteins in plasma (By similarity)
Tgm2	Tgm2 - transglutaminase 2, C polypeptide; Catalyzes the cross-linking of proteins and the conjugation of polyamines to proteins
Thap3	Thap3 - THAP domain containing, apoptosis associated protein 3; Component of a THAP1/THAP3-HCFC1-OGT complex that is required for the regulation of the transcriptional activity of RRM1 (By similarity)
Thbs1	Thbs1 - thrombospondin 1; Adhesive glycoprotein that mediates cell-to-cell and cell-to-matrix interactions. Binds heparin. May play a role in dentinogenesis and/or maintenance of dentin and dental pulp. Ligand for CD36 mediating antiangiogenic properties (By similarity)
Tiparp	Tiparp - TCDD-inducible poly(ADP-ribose) polymerase; Poly [ADP-ribose] polymerase using NAD(+) as a substrate to transfer ADP-ribose onto glutamic acid residues of a protein acceptor; repeated rounds of ADP-ribosylation leads to the formation of poly(ADPribose) chains on the protein, thereby altering the function of the target protein. May play a role in the adaptative response to chemical exposure (TCDD) and thereby mediates certain effects of the chemicals
Tmem159	Tmem159 - transmembrane protein 159
Tmem191c	Tmem191c - transmembrane protein 191C
Tmem200a	Tmem200a - transmembrane protein 200A
Tmem254b	Tmem254b - transmembrane protein 254b; Gm9746 - predicted gene 9746
Tmem38b	Tmem38b - transmembrane protein 38B; Monovalent cation channel required for maintenance of rapid intracellular calcium release. May act as a potassium counter-ion channel that functions in synchronization with calcium release from intracellular stores
Tmem52	Tmem52 - transmembrane protein 52
Tmem86a	Tmem86a - transmembrane protein 86A
Tob2	Tob2 - transducer of ERBB2, 2
Trank1	Trank1 - tetratricopeptide repeat and ankyrin repeat containing 1
Trib1	Trib1 - tribbles homolog 1 (Drosophila); Interacts with MAPK kinases and regulates activation of MAP kinases. May not display kinase activity (By similarity)
Trim12c	Trim12c - tripartite motif-containing 12C
Trim21	Trim21 - tripartite motif-containing 21; E3 ubiquitin-protein ligase whose activity is dependent on E2 enzymes, UBE2D1, UBE2D2, UBE2E1 and UBE2E2. Forms a ubiquitin ligase complex in cooperation with the E2 UBE2D2 that is used not only for the ubiquitination of USP4 and IKBKB but also for its self-ubiquitination. Component of cullin-RING-based SCF (SKP1-CUL1-F-box protein) E3 ubiquitin-protein ligase complexes such as SCF(SKP2)-like complexes. A TRIM21-containing SCF(SKP2)- like complex is shown to mediate ubiquitination of CDKN1B ('Thr- 187' phosphorylated-form)

Trim30a	Trim30a - tripartite motif-containing 30A; Trans-acting factor that regulates gene expression of interleukin 2 receptor alpha chain. May affect IL2R-alpha expression through cis-acting negative regulatory elements or through competition with proteins that bind to enhancer or activator sequences. Negatively regulates Toll-like receptor (TLR)-mediated activation of NFKB by promoting degradation of TAB2 and TAB3 and preventing TRAF6 autoubiquitination. Negatively regulates production of reactive oxygen species (ROS) which inhibits activation of the NLRP3 inflammasome complex.
Trim30d	Trim30d - tripartite motif-containing 30D
Trim34a	Trim34a - tripartite motif-containing 34A
Trmt5	Trmt5 - TRM5 tRNA methyltransferase 5 homolog (S. cerevisiae); Specifically methylates the N1 position of guanosine-37 in various cytoplasmic and mitochondrial tRNAs. Methylation is not dependent on the nature of the nucleoside 5' of the target nucleoside. This is the first step in the biosynthesis of wybutosine (yW), a modified base adjacent to the anticodon of tRNAs and required for accurate decoding (By similarity)
Tsku	Tsku - tsukushi
Ttc30b	Ttc30b - tetratricopeptide repeat domain 30B; Required for polyglutamylation of axonemal tubulin (By similarity). Plays a role in anterograde intraflagellar transport (IFT), the process by which cilia precursors are transported from the base of the cilium to the site of their incorporation at the tip (By similarity)
Tuba1b	Tuba1b - tubulin, alpha 1B; Tubulin is the major constituent of microtubules. It binds two moles of GTP, one at an exchangeable site on the beta chain and one at a non-exchangeable site on the alpha chain (By similarity)
Tuba4a	Tuba4a - tubulin, alpha 4A; Tubulin is the major constituent of microtubules. It binds two moles of GTP, one at an exchangeable site on the beta chain and one at a non-exchangeable site on the alpha chain
Tuft1	Tuft1 - tuftelin 1; Involved in the mineralization and structural organization of enamel
Txndc11	Txndc11 - thioredoxin domain containing 11; May act as a redox regulator involved in DUOX proteins folding. The interaction with DUOX1 and DUOX2 suggest that it belongs to a multiprotein complex constituting the thyroid H(2)O(2) generating system. It is however not sufficient to assist DUOX1 and DUOX2 in H(2)O(2) generation (By similarity)
Ubc	Ubc - ubiquitin C
Vcam1	Vcam1 - vascular cell adhesion molecule 1; Important in cell-cell recognition. Appears to function in leukocyte-endothelial cell adhesion. Interacts with the beta-1 integrin VLA4 on leukocytes, and mediates both adhesion and signal transduction. The VCAM1/VLA4 interaction may play a pathophysiologic role both in immune responses and in leukocyte emigration to sites of inflammation
Vit	Vit - vitrin; Promotes matrix assembly and cell adhesiveness
Vps37b	Vps37b - vacuolar protein sorting 37B (yeast); Component of the ESCRT-I complex, a regulator of vesicular trafficking process. Required for the sorting of endocytic ubiquitinated cargos into multivesicular bodies. May be involved in cell growth and differentiation (By similarity)
Vsnl1	Vsnl1 - visinin-like 1; Regulates (in vitro) the inhibition of rhodopsin

	phosphorylation in a calcium-dependent manner (By similarity)
Vwa1	Vwa1 - von Willebrand factor A domain containing 1; Promotes matrix assembly
Wdfy1	Wdfy1 - WD repeat and FYVE domain containing 1
Wfdc1	Wfdc1 - WAP four-disulfide core domain 1; Has growth inhibitory activity (By similarity)
Wfdc17	Wfdc17 - WAP four-disulfide core domain 17
WI1-49P9.2	Predicted gene 43549
Wnt6	Wnt6 - wingless-related MMTV integration site 6; Ligand for members of the frizzled family of seven transmembrane receptors. Probable developmental protein. May be a signaling molecule which affects the development of discrete regions of tissues. Is likely to signal over only few cell diameters
Xaf1	Xaf1 - XIAP associated factor 1; Seems to function as a negative regulator of members of the IAP (inhibitor of apoptosis protein) family. Inhibits anti- caspase activity of BIRC4. Induces cleavage and inactivation of BIRC4 independent of caspase activation. Mediates TNF-alpha- induced apoptosis and is involved in apoptosis in trophoblast cells. May inhibit BIRC4 indirectly by activating the mitochondrial apoptosis pathway. After translocation to mitochondra, promotes translocation of BAX to mitochondria and cytochrome c release from mitochondria.
Zfp280b	Zfp280b - zinc finger protein 280B
Zfp36	Zfp36 - zinc finger protein 36; mRNA-binding protein involved in post-transcriptional regulation of AU-rich element (ARE)-containing mRNAs. Acts by specifically binding ARE-containing mRNAs and promoting their degradation. Recruits deadenylase CNOT7 (and probably the CCR4-NOT complex) via association with CNOT1. Plays a key role in the post-transcriptional regulation of tumor necrosis factor (TNF)
Zfp36l2	Zfp36l2 - zinc finger protein 36, C3H type-like 2; Probable regulatory protein involved in regulating the response to growth factors. RNA-binding protein that binds to 5'UUAUUUAUUU-3' core sequence. Binds to the class II AU-rich element (ARE) in the 3'-UTR of target mRNAs and promotes their deadenylation and degradation (By similarity)
Zfp524	Zfp524 - zinc finger protein 524; May be involved in transcriptional regulation
Zfp58	Zfp58 - zinc finger protein 58; May have a role during differentiation processes
Zfp580	Zfp580 - zinc finger protein 580; May be involved in transcriptional regulation
Zfp825	Zfp825 - zinc finger protein 825
Zfp933	Zfp933 - zinc finger protein 933
Zfp955b	Zfp955b - zinc finger protein 955B
Zfp958	Zfp958 - zinc finger protein 958
Zkscan3	Zkscan3 - zinc finger with KRAB and SCAN domains 3; Transcriptional factor that binds to the consensus sequence 5'-[GT][AG][AGT]GGGG-3' and acts as a repressor of autophagy. Specifically represses expression of genes involved in autophagy and lysosome biogenesis/function such as MAP1LC3B, ULK1 or WIPI2. Associates with chromatin at the ITGB4 and VEGF promoters (By similarity)
Znf41-ps	Gm13139 - predicted gene 13139
Znhit2	Znhit2 - zinc finger, HIT domain containing 2

Zswim7	Zswim7 - zinc finger, SWIM-type containing 7; Involved in early stages of the homologous recombination repair (HRR) pathway of double-stranded DNA breaks arising during DNA replication or induced by DNA-damaging agents (By similarity)
