# Supplementary material for: NNT is a key regulator of adrenal redox homeostasis and steroidogenesis in male mice
Source: J Endocrinol. 2017 Oct 18;236(1):13–28. doi: 10.1530/JOE-16-0638 (PMC5744559; doi:10.1530/JOE-16-0638)
Supplement: Supporting Table 3 [file erc-236-13-t003.pdf]

Supplementary Table 3. Differential gene expression between *Nnt*$^{+/+}$ and *Nnt*$^{-/-}$: Genes downregulated in *Nnt*$^{-/-}$

Gene Symbol	p-value	Fold Change	Gene Description
Nnt	0.01171	3.67	Nnt - nicotinamide nucleotide transhydrogenase; The transhydrogenation between NADH and NADP is coupled to respiration and ATP hydrolysis and functions as a proton pump across the membrane. May play a role in reactive oxygen species (ROS) detoxification in the adrenal gland (By similarity)
Wdfy1	0.00000	2.84	Wdfy1 - WD repeat and FYVE domain containing 1
Vsnl1	0.01405	2.51	Vsnl1 - visinin-like 1; Regulates (in vitro) the inhibition of rhodopsin phosphorylation in a calcium-dependent manner (By similarity)
Cep85	0.00001	2.29	Ccdc21 - coiled-coil domain containing 21
Gm28382	0.00233	2.28	predicted gene 28382 known lincRNA
Gm26880	0.00001	2.10	predicted gene, 26880 known lincRNA
RP24-378G4.3	0.00009	2.09	predicted gene 43305, known TEC
Ccdc160	0.00889	2.09	Ccdc160 - coiled-coil domain containing 160
Nat8	0.00046	2.03	Nat8 - N-acetyltransferase 8 (GCN5-related, putative); Plays a role in regulation of gastrulation
Xaf1	0.01555	1.94	Xaf1 - XIAP associated factor 1; Seems to function as a negative regulator of members of the IAP (inhibitor of apoptosis protein) family. Inhibits anti- caspase activity of BIRC4. Induces cleavage and inactivation of BIRC4 independent of caspase activation. Mediates TNF-alpha- induced apoptosis and is involved in apoptosis in trophoblast cells. May inhibit BIRC4 indirectly by activating the mitochondrial apoptosis pathway. After translocation to mitochondra, promotes translocation of BAX to mitochondria and cytochrome c release from mitochondria.
RP23-338P12.2	0.02081	1.91	RIKEN cDNA 7330423F06
1700019G17Rik	0.00424	1.88	ENSMUSG00000068299 - RIKEN cDNA 1700019G17 gene
A530020G20Rik	0.00648	1.87	RIKEN cDNA A530020G20 gene
Cml5	0.00164	1.86	Cml5 - camello-like 5; May play a role in regulation of gastrulation
Hhex	0.03372	1.86	Hhex - hematopoietically expressed homeobox; Recognizes the DNA sequence 5'-ATTAA-3' (By similarity). Transcriptional repressor. May play a role in hematopoietic differentiation. Establishes anterior identity at two levels; acts early to enhance canonical WNT-signaling by repressing expression of TLE4, and acts later to inhibit NODAL-

			signaling by directly targeting NODAL
Tmem52	0.01632	1.83	Tmem52 - transmembrane protein 52
Lefty1	0.00084	1.82	Lefty1 - left right determination factor 1; Required for left-right axis determination as a regulator of LEFTY2 and NODAL
Trim30a	0.04656	1.81	Trim30a - tripartite motif-containing 30A; Trans-acting factor that regulates gene expression of interleukin 2 receptor alpha chain. May affect IL2R-alpha expression through cis-acting negative regulatory elements or through competition with proteins that bind to enhancer or activator sequences. Negatively regulates Toll-like receptor (TLR)-mediated activation of NFKB by promoting degradation of TAB2 and TAB3 and preventing TRAF6 autoubiquitination. Negatively regulates production of reactive oxygen species (ROS) which inhibits activation of the NLRP3 inflammasome complex. This, in turn, regulat [...]
Rmrp	0.02472	1.79	RNA component of mitochondrial RNA processing endoribonuclease
Epb4.1l4aos	0.00889	1.78	erythrocyte membrane protein band 4.1 like 4a, opposite strand
Steap1	0.01346	1.73	Steap1 - six transmembrane epithelial antigen of the prostate 1; Metalloreductase that has the ability to reduce both $Fe(3+)$ to $Fe(2+)$ and $Cu(2+)$ to $Cu(1+)$. Uses NAD(+) as acceptor
Cyp21a2-ps	0.02491	1.73	Cyp21a2 pseudogene
Aldh3b1	0.04854	1.72	Aldh3b1 - aldehyde dehydrogenase 3 family, member B1; Oxidizes medium and long chain saturated and unsaturated aldehydes (By similarity). Metabolizes also benzaldehyde (By similarity). Low activity towards acetaldehyde and 3,4-dihydroxyphenylacetaldehyde (By similarity). May not metabolize short chain aldehydes (By similarity). May use both NADP(+) and NAD(+) as cofactors (By similarity). May have a protective role against the cytotoxicity induced by lipid peroxidation (By similarity)
Ifi44	0.03833	1.71	Ifi44 - interferon-induced protein 44; This protein aggregates to form microtubular structures (By similarity)

Pik3ap1	0.04566	1.71	Pik3ap1 - phosphoinositide-3-kinase adaptor protein 1; Signaling adapter that contributes to B-cell development by linking B-cell receptor (BCR) signaling to the phosphoinositide 3-kinase (PI3K)-Akt signaling pathway. Has a complementary role to the BCR coreceptor CD19, coupling BCR and PI3K activation by providing a docking site for the PI3K subunit PIK3R1. Alternatively, links Toll-like receptor (TLR) signaling to PI3K activation, a process preventing excessive inflammatory cytokine production. Also involved in the activation of PI3K in natural killer cells. May be involved in the survival of [...]
4930426D05Rik	0.00337	1.70	RIKEN cDNA 4930426D05 gene
Pacsin3	0.00221	1.70	Pacsin3 - protein kinase C and casein kinase substrate in neurons 3; May play a role in endocytosis
Ccdc114	0.00064	1.69	Ccdc114 - coiled-coil domain containing 114; Probable component of the outer dynein arm complex required along the entire axoneme for tethering of outer dynein arms (By similarity)
Kcnn2	0.00852	1.69	Kcnn2 - potassium intermediate/small conductance calcium-activated channel, subfamily N, member 2; Forms a voltage-independent potassium channel activated by intracellular calcium. Activation is followed by membrane hyperpolarization. Thought to regulate neuronal excitability by contributing to the slow component of synaptic afterhyperpolarization. The channel is blocked by apamin (By similarity)
E330017L17Rik	0.02207	1.68	RIKEN cDNA E330017L17 gene
Trim30d	0.00569	1.67	Trim30d - tripartite motif-containing 30D
Susd3	0.00939	1.67	Susd3 - sushi domain containing 3
Vcam1	0.01736	1.67	Vcam1 - vascular cell adhesion molecule 1; Important in cell-cell recognition. Appears to function in leukocyte-endothelial cell adhesion. Interacts with the beta-1 integrin VLA4 on leukocytes, and mediates both adhesion and signal transduction. The VCAM1/VLA4 interaction may play a pathophysiologic role both in immune responses and in leukocyte emigration to sites of inflammation
Gas2	0.01473	1.66	Gas2 - growth arrest specific 2; May play a role in apoptosis by acting as a cell death substrate for caspases. Is cleaved during apoptosis and the cleaved form induces dramatic rearrangements of the actin cytoskeleton and potent changes in the

			shape of the affected cells. May play a role in chondrocyte proliferation and differentiation, and in limb myogenesis. May be involved in the regulation of the apoptosis in the interdigital tissues of the developing hindlimb. May be involved in the membrane ruffling process
Slc45a3	0.00342	1.65	Slc45a3 - solute carrier family 45, member 3
Lmln	0.00426	1.65	Lmln - leishmanolysin-like (metallopeptidase M8 family); Metalloprotease essential for the coordination of mitotic progression, and also plays a role in cell migration (By similarity)
Akr1cl	0.00876	1.65	Akr1cl - aldo-keto reductase family 1, member C-like
Gnat2	0.01737	1.65	Gnat2 - guanine nucleotide binding protein, alpha transducing 2; Guanine nucleotide-binding proteins (G proteins) are involved as modulators or transducers in various transmembrane signaling systems. Transducin is an amplifier and one of the transducers of a visual impulse that performs the coupling between rhodopsin and cGMP-phosphodiesterase
Ctxn3	0.02400	1.64	Ctxn3 - cortexin 3
Bloc1s3	0.00127	1.64	Bloc1s3 - biogenesis of lysosome-related organelles complex-1, subunit 3; Component of the BLOC-1 complex, a complex that is required for normal biogenesis of lysosome-related organelles (LRO), such as platelet dense granules and melanosomes. In concert with the AP-3 complex, the BLOC-1 complex is required to target membrane protein cargos into vesicles assembled at cell bodies for delivery into neurites and nerve terminals. The BLOC-1 complex, in association with SNARE proteins, is also proposed to be involved in neurite extension. Plays a role in intracellular vesicle trafficking
Ism1	0.00665	1.63	Ism1 - isthmin 1 homolog (zebrafish)
1500015O10Rik	0.02421	1.63	Ecrg4 - RIKEN cDNA 1500015O10 gene; Probable hormone that may induce senescence of oligodendrocyte and neural precursor cells, characterized by G1 arrest, RB1 dephosphorylation and accelerated CCND1 and CCND3 proteasomal degradation
Cfl2	0.00865	1.62	Cfl2 - cofilin 2, muscle; Controls reversibly actin polymerization and depolymerization in a pH-sensitive manner. It has the ability to bind G- and F-actin in a 1:1 ratio of cofilin to actin. It is the major component of intranuclear and cytoplasmic actin rods

Nr1h4	0.02873	1.62	Nr1h4 - nuclear receptor subfamily 1, group H, member 4; Ligand-activated transcription factor. Receptor for bile acids such as chenodeoxycholic acid, lithocholic acid and deoxycholic acid. Represses the transcription of the cholesterol 7-alpha-hydroxylase gene (CYP7A1) and activates the intestinal bile acid-binding protein (IBABP). Activates the transcription of bile salt export pump ABCB11 by directly recruiting histone methyltransferase CARM1 within its gene locus (By similarity)
Zfp933	0.00646	1.61	Zfp933 - zinc finger protein 933
Serpinb6b	0.03482	1.61	Serpinb6b - serine (or cysteine) peptidase inhibitor, clade B, member 6b
Lilr4b	0.02638	1.60	leukocyte immunoglobulin-like receptor, subfamily B, member 4B
Tmem200a	0.02581	1.60	Tmem200a - transmembrane protein 200A
Pyroxd2	0.02401	1.60	Pyroxd2 - pyridine nucleotide-disulphide oxidoreductase domain 2; Probable oxidoreductase (By similarity)
Fdxr	0.03391	1.59	Fdxr - ferredoxin reductase; Serves as the first electron transfer protein in all the mitochondrial P450 systems. Including cholesterol side chain cleavage in all steroidogenic tissues, steroid 11-beta hydroxylation in the adrenal cortex, 25-OH-vitamin D3-24 hydroxylation in the kidney, and sterol C-27 hydroxylation in the liver
AA986860	0.00029	1.59	AA986860 - expressed sequence AA986860; Putative androgen-specific receptor (By similarity)
Cd59a	0.00024	1.59	Cd59a - CD59a antigen; Potent inhibitor of the complement membrane attack complex (MAC) action. Acts by binding to the C8 and/or C9 complements of the assembling MAC, thereby preventing incorporation of the multiple copies of C9 required for complete formation of the osmolytic pore (By similarity)
Dbp	0.00106	1.58	Dbp - D site albumin promoter binding protein; This transcriptional activator recognizes and binds to the sequence 5'-RTTAYGTAAY-3' found in the promoter of genes such as albumin, CYP2A4 and CYP2A5. It is not essential for circadian rhythm generation, but modulates important clock output genes. May be a direct target for regulation by the circadian pacemaker component clock. May affect circadian period and sleep regulation (By similarity)
Tmem191c	0.00884	1.58	Tmem191c - transmembrane protein 191C
RP23-371A16.3	0.01090	1.58	RIKEN cDNA 4932422M17 gene

Gna14	0.01154	1.58	Gna14 - guanine nucleotide binding protein, alpha 14; Guanine nucleotide-binding proteins (G proteins) are involved as modulators or transducers in various transmembrane signaling systems
Syne4	0.03556	1.58	AI428936 - expressed sequence AI428936; Contributes to the establishment of secretory epithelial morphology, by promoting kinesin-dependent apical migration of the centrosome and Golgi apparatus and basal localization of the nucleus
Tmem86a	0.01401	1.58	Tmem86a - transmembrane protein 86A
Gm28048	0.01357	1.57	predicted gene, 28048
C1qtnf6	0.01602	1.57	C1qtnf6 - C1q and tumor necrosis factor related protein 6
Dhx58	0.00430	1.57	Dhx58 - DEXH (Asp-Glu-X-His) box polypeptide 58; Acts as a regulator of DDX58/RIG-I and IFIH1/MDA5 mediated antiviral signaling. Cannot initiate antiviral signaling as it lacks the CARD domain required for activating MAVS/IPS1-dependent signaling events. Can have both negative and positive regulatory functions related to DDX58/RIG-I and IFIH1/MDA5 signaling and this role in regulating signaling may be complex and could probably depend on characteristics of the infecting virus or target cells, or both. Its inhibitory action on DDX58/RIG-I signaling may involve the following mechanisms: competi [...]
Plxnb1	0.01115	1.56	Plxnb1 - plexin B1; Receptor for SEMA4D. Plays a role in RHOA activation and subsequent changes of the actin cytoskeleton. Plays a role in axon guidance, invasive growth and cell migration
RP24-230J14.5	0.00049	1.56	predicted gene 42427
Cybb	0.04555	1.56	Cybb - cytochrome b-245, beta polypeptide; Critical component of the membrane-bound oxidase of phagocytes that generates superoxide. It is the terminal component of a respiratory chain that transfers single electrons from cytoplasmic NADPH across the plasma membrane to molecular oxygen on the exterior. Also functions as a voltage-gated proton channel that mediates the H(+) currents of resting phagocytes
Prss35	0.02639	1.56	Prss35 - protease, serine, 35
Ajuba	0.00502	1.56	Jub - ajuba; Adapter or scaffold protein which participates in the assembly of numerous protein complexes and is involved in several cellular processes such as cell fate determination, cytoskeletal organization, repression of gene transcription, mitosis, cell-

			cell adhesion, cell differentiation, proliferation and migration. Contributes to the linking and/or strengthening of epithelia cell- cell junctions in part by linking adhesive receptors to the actin cytoskeleton. May be involved in signal transduction from cell adhesion sites to the nucleus. Plays an important role in regulation of the [...]
9330151L19Rik	0.00141	1.55	ENSMUSG00000097061 - Uncharacterized protein
Tuft1	0.00359	1.55	Tuft1 - tuftelin 1; Involved in the mineralization and structural organization of enamel
Nek5	0.00906	1.54	Nek5 - NIMA (never in mitosis gene a)-related expressed kinase 5
Bmp7	0.01911	1.54	Bmp7 - bone morphogenetic protein 7; Induces cartilage and bone formation. May be the osteoinductive factor responsible for the phenomenon of epithelial osteogenesis. Plays a role in calcium regulation and bone homeostasis
Paip1	0.00064	1.54	Paip1 - polyadenylate binding protein-interacting protein 1; Acts as a coactivator in the regulation of translation initiation of poly(A)-containing mRNAs. Its stimulatory activity on translation is mediated via its action on PABPC1. Competes with PAIP2 for binding to PABPC1. Its association with EIF4A and PABPC1 may potentiate contacts between mRNA termini. May also be involved in translationally coupled mRNA turnover. Implicated with other RNA-binding proteins in the cytoplasmic deadenylation/translational and decay interplay of the FOS mRNA mediated by the major coding-region determinant of [...]
Rabep2	0.02270	1.53	Rabep2 - rabaptin, RAB GTPase binding effector protein 2; Plays a role in membrane trafficking and in homotypic early endosome fusion (By similarity)
Hmgn3	0.02013	1.53	Hmgn3 - high mobility group nucleosomal binding domain 3; Binds to nucleosomes, regulating chromatin structure and consequently, chromatin-dependent processes such as transcription, DNA replication and DNA repair. Affects both insulin and glucagon levels and modulates the expression of pancreatic genes involved in insulin secretion. Regulates the expression of the glucose transporter SLC2A2 by binding specifically to its promoter region and recruiting PDX1 and additional transcription factors.

Pgm5	0.01192	1.53	Pgm5 - phosphoglucomutase 5; Component of adherens-type cell-cell and cell-matrix junctions. Lacks phosphoglucomutase activity (By similarity)
Cacna1c	0.00472	1.53	Cacna1c - calcium channel, voltage-dependent, L type, alpha 1C subunit; Voltage-sensitive calcium channels (VSCC) mediate the entry of calcium ions into excitable cells and are also involved in a variety of calcium-dependent processes, including muscle contraction, hormone or neurotransmitter release, gene expression, cell motility, cell division and cell death. The isoform alpha-1C gives rise to L-type calcium currents. Long-lasting (L-type) calcium channels belong to the 'high-voltage activated' (HVA) group. They are blocked by dihydropyridines (DHP), phenylalkylamines, benzothiazepines, and b [...]
Ccdc51	0.02169	1.53	Ccdc51 - coiled-coil domain containing 51
Sox12	0.01240	1.53	Sox12 - SRY-box containing gene 12; Binds to the sequence 5'-AACAAT-3' (By similarity)
Tbx3os1	0.01001	1.53	T-box 3, opposite strand 1
Izumo4	0.01172	1.53	Izumo4 - IZUMO family member 4
Parp16	0.02295	1.53	Parp16 - poly (ADP-ribose) polymerase family, member 16; Mono-ADP-ribosyltransferase targeting the karyopherin KPNB1. Plays a role in unfolded protein response (UPR), by ADP- ribosylating and activating EIF2AK3 and ERN1, two important UPR effectors (By similarity)
Ly96	0.01027	1.53	Ly96 - lymphocyte antigen 96; Cooperates with TLR4 in the innate immune response to bacterial lipopolysaccharide (LPS), and with TLR2 in the response to cell wall components from Gram-positive and Gram-negative bacteria. Enhances TLR4-dependent activation of NF-kappa-B. Cells expressing both MD2 and TLR4, but not TLR4 alone, respond to LPS (By similarity)
Arrdc2	0.00305	1.52	Arrdc2 - arrestin domain containing 2
Hoxb2	0.03182	1.52	Hoxb2 - homeobox B2; Sequence-specific transcription factor which is part of a developmental regulatory system that provides cells with specific positional identities on the anterior-posterior axis (By similarity)
Rabep1	0.03243	1.52	Rabep1 - rabaptin, RAB GTPase binding effector protein 1; Rab effector protein acting as linker between gamma- adaptin, RAB4A and RAB5A. Involved in endocytic membrane fusion and membrane trafficking of recycling endosomes.

Trim21	0.03171	1.52	Trim21 - tripartite motif-containing 21; E3 ubiquitin-protein ligase whose activity is dependent on E2 enzymes, UBE2D1, UBE2D2, UBE2E1 and UBE2E2. Forms a ubiquitin ligase complex in cooperation with the E2 UBE2D2 that is used not only for the ubiquitination of USP4 and IKBKB but also for its self-ubiquitination. Component of cullin-RING-based SCF (SKP1-CUL1-F-box protein) E3 ubiquitin-protein ligase complexes such as SCF(SKP2)-like complexes. A TRIM21-containing SCF(SKP2)- like complex is shown to mediate ubiquitination of CDKN1B ('Thr- 187' phosphorylated-form), thereby promoting its degradat [...]
C920006O11Rik	0.00291	1.51	RIKEN cDNA C920006O11 gene
Trim12c	0.01944	1.51	Trim12c - tripartite motif-containing 12C
Fads6	0.01991	1.51	Fads6 - fatty acid desaturase domain family, member 6
Mast4	0.00722	1.51	Mast4 - microtubule associated serine/threonine kinase family member 4
BC002059	0.02431	1.51	BC002059 - cDNA sequence BC002059
Steap2	0.00982	1.51	Steap2 - six transmembrane epithelial antigen of prostate 2; Metalloreductase that has the ability to reduce both Fe(3+) to Fe(2+) and Cu(2+) to Cu(1+). Uses NAD(+) as acceptor
AA474331	0.00129	1.51	AA474331 - Uncharacterized protein
Snx32	0.00977	1.51	Snx32 - sorting nexin 32; May be involved in several stages of intracellular trafficking (By similarity)
Aldh1a1	0.01696	1.51	Aldh1a1 - aldehyde dehydrogenase family 1, subfamily A1; In addition to the activity on acetaldehyde and related substrates, is also involved in the oxidation of aldehydes derived from biogenic amines such as epinephrine and norepinephrine, as well as the aldehydes generated via lipid peroxidation. Binds free retinal and cellular retinol-binding protein-bound retinal. Can convert/oxidize retinaldehyde to retinoic acid (By similarity)
Gsg1l	0.04941	1.51	Gsg1l - GSG1-like; As a component of the inner core of AMPAR complexes, modifies AMPA receptor (AMPAR) gating (By similarity)
Snhg4	0.00378	1.50	Small nucleolar RNA host gene 4
Tsku	0.01974	1.50	Tsku - tsukushi
Slc2a4rg-ps	0.02074	1.50	Slc2a4 regulator, pseudogene
