# Supplementary material for: NNT is a key regulator of adrenal redox homeostasis and steroidogenesis in male mice
Source: J Endocrinol. 2017 Oct 18;236(1):13–28. doi: 10.1530/JOE-16-0638 (PMC5744559; doi:10.1530/JOE-16-0638)
Supplement: Supporting Table 4 [file erc-236-13-t004.pdf]

Supplementary Table 4. Differential gene expression beween *Nnt*$^{+/+}$ and *Nnt*$^{-/-}$: Genes upregulated in *Nnt*$^{-/-}$

Gene Symbol	p-value	Fold Change	Gene Description
Hspa1b	0.04946	30.96	Hspa1b - heat shock protein 1B; In cooperation with other chaperones, Hsp70s stabilize preexistent proteins against aggregation and mediate the folding of newly translated polypeptides in the cytosol as well as within organelles. These chaperones participate in all these processes through their ability to recognize nonnative conformations of other proteins. They bind extended peptide segments with a net hydrophobic character exposed by polypeptides during translation and membrane translocation, or following stress-induced damage (By similarity)
Dnajb1	0.03916	6.58	Dnajb1 - DnaJ (Hsp40) homolog, subfamily B, member 1; Interacts with HSP70 and can stimulate its ATPase activity. Stimulates the association between HSC70 and HIP (By similarity)
Nr4a2	0.02724	6.37	Nr4a2 - nuclear receptor subfamily 4, group A, member 2; Transcriptional regulator which is important for the differentiation and maintenance of meso-diencephalic dopaminergic (mdDA) neurons during development. It is crucial for expression of a set of genes such as SLC6A3, SLC18A2, TH and DRD2 which are essential for development of mdDA neurons
Hba-a1	0.01335	4.99	Hba-a1 - hemoglobin alpha, adult chain 1; Involved in oxygen transport from the lung to the various peripheral tissues (By similarity)
Hba-a2	0.01337	4.89	Hba-a2 - hemoglobin alpha, adult chain 2; Involved in oxygen transport from the lung to the various peripheral tissues (By similarity)
Hbb-bt	0.02493	4.88	Hemoglobin, beta adult t chain
Gm12840	0.02495	4.30	Predicted gene 12840
Cyr61	0.04753	4.29	Cyr61 - cysteine rich protein 61; Promotes cell proliferation, chemotaxis, angiogenesis and cell adhesion. Appears to play a role in wound healing by up- regulating, in skin fibroblasts, the expression of a number of genes involved in angiogenesis, inflammation and matrix remodeling including VEGA-A, VEGA-C, MMP1, MMP3, TIMP1, uPA, PAI-1 and integrins alpha-3 and alpha-5 (By similarity). CYR61-mediated gene regulation is dependent on heparin-binding (By similarity). Down-regulates the expression of alpha-1 and alpha-2 subunits of collagen type-1 (By similarity). Promotes cell adhesion and adh [...]

Ier3	0.03180	4.27	Ier3 - immediate early response 3; May play a role in the ERK signaling pathway by inhibiting the dephosphorylation of ERK by phosphatase PP2A- PPP2R5C holoenzyme. Acts also as an ERK downstream effector mediating survival (By similarity). As a member of the NUPR1/RELB/IER3 survival pathway, may allow the development of pancreatic intraepithelial neoplasias
Hbb-bs	0.01648	4.18	Hemoglobin, beta adult s chain
Gm26809	0.02990	3.64	Predicted gene, 26809
Vps37b	0.04093	3.44	Vps37b - vacuolar protein sorting 37B (yeast); Component of the ESCRT-I complex, a regulator of vesicular trafficking process. Required for the sorting of endocytic ubiquitinated cargos into multivesicular bodies. May be involved in cell growth and differentiation (By similarity)
Adamts1	0.04035	3.23	Adamts1 - a disintegrin-like and metallopeptidase (reprolysin type) with thrombospondin type 1 motif, 1; Cleaves aggrecan, a cartilage proteoglycan, and may be involved in its turnover. Has angiogenic inhibitor activity (By similarity). Active metalloprotease, which may be associated with various inflammatory processes as well as development of cancer cachexia. May play a critical role in follicular rupture (By similarity)
Cbarp	0.03781	3.22	Calcium channel, voltage-dependent, beta subunit associated regulatory protein
Gem	0.03568	3.22	Gem - GTP binding protein (gene overexpressed in skeletal muscle); Could be a regulatory protein, possibly participating in receptor-mediated signal transduction at the plasma membrane. Has guanine nucleotide-binding activity but undetectable intrinsic GTPase activity
Thbs1	0.04211	3.15	Thbs1 - thrombospondin 1; Adhesive glycoprotein that mediates cell-to-cell and cell-to-matrix interactions. Binds heparin. May play a role in dentinogenesis and/or maintenance of dentin and dental pulp. Ligand for CD36 mediating antiangiogenic properties (By similarity)
Ier2	0.04946	3.14	Ier2 - immediate early response 2
Gm23262	0.03237	3.06	Predicted gene, 23262
Klf4	0.02903	3.03	Klf4 - Kruppel-like factor 4 (gut); Transcription factor; can act both as activator and as repressor. Binds the 5'-CACCC-3' core sequence. Binds to the promoter region of its own gene and can activate its own transcription. Regulates the expression of key transcription factors during embryonic

			development. Plays an important role in maintaining embryonic stem cells, and in preventing their differentiation. Required for establishing the barrier function of the skin and for postnatal maturation and maintenance of the ocular surface. Involved in the differentiation of epithelial cells and may al [...]
Egr1	0.03518	2.99	Egr1 - early growth response 1; Transcriptional regulator. Recognizes and binds to the DNA sequence 5'-CGCCCCCGC-3'(EGR-site). Activates the transcription of target genes whose products are required for mitogenesis and differentiation
Gm13889	0.04460	2.88	Predicted gene 13889
Tiparp	0.02564	2.70	Tiparp - TCDD-inducible poly(ADP-ribose) polymerase; Poly [ADP-ribose] polymerase using NAD(+) as a substrate to transfer ADP-ribose onto glutamic acid residues of a protein acceptor; repeated rounds of ADP-ribosylation leads to the formation of poly(ADPribose) chains on the protein, thereby altering the function of the target protein. May play a role in the adaptative response to chemical exposure (TCDD) and thereby mediates certain effects of the chemicals
2010109I03Rik	0.04310	2.66	ENSMUSG00000063522 - RIKEN cDNA 2010109I03 gene
Dgat1	0.02534	2.59	Dgat1 - diacylglycerol O-acyltransferase 1; Catalyzes the terminal and only committed step in triacylglycerol synthesis by using diacylglycerol and fatty acyl CoA as substrates. In contrast to DGAT2 it is not essential for survival. May be involved in VLDL (very low density lipoprotein) assembly. In liver, plays a role in esterifying exogenous fatty acids to glycerol. Functions as the major acyl-CoA retinol acyltransferase (ARAT) in the skin, where it acts to maintain retinoid homeostasis and prevent retinoid toxicity leading to skin and hair disorders
Cwc25	0.02343	2.57	Cwc25 - CWC25 spliceosome-associated protein homolog (S. cerevisiae)
Hspb1	0.04540	2.47	Hspb1 - heat shock protein 1; Involved in stress resistance and actin organization
Pmvk	0.03390	2.43	Pmvk - phosphomevalonate kinase
Gm9780	0.00006	2.40	Gm10393 - predicted gene 10393
Sik2	0.04985	2.29	Sik2 - salt inducible kinase 2; Phosphorylates 'Ser-789' of IRS1 in insulin-stimulated adipocytes, potentially modulating the efficiency of insulin signal transduction. Inhibits CREB activity by phosphorylating and repressing TORCs, the CREB-specific coactivators
Plac9a	0.00061	2.24	Gm10393 - predicted gene 10393

Ptger3	0.02793	2.18	Ptger3 - prostaglandin E receptor 3 (subtype EP3); Receptor for prostaglandin E2 (PGE2); the EP3 receptor may be involved in inhibition of gastric acid secretion, modulation of neurotransmitter release in central and peripheral neurons, inhibition of sodium and water reabsorption in kidney tubulus and contraction in uterine smooth muscle. The activity of this receptor can couple to both the inhibition of adenylate cyclase mediated by G(i) proteins, and to an elevation of intracellular calcium. The various forms can interact with different second messenger systems
Insig1	0.03712	2.17	Insig1 - insulin induced gene 1; Mediates feedback control of cholesterol synthesis by controlling SCAP and HMGCR. Functions by blocking the processing of sterol regulatory element-binding proteins (SREBPs). Capable of retaining the SCAP-SREBF2 complex in the ER thus preventing it from escorting SREBPs to the Golgi. Initiates the sterol-mediated ubiquitin-mediated endoplasmic reticulum-associated degradation (ERAD) of HMGCR via recruitment of the reductase to the ubiquitin ligase, AMFR/gp78. May play a role in growth and differentiation of tissues involved in metabolic control. May play a regul [...]
Midn	0.03666	2.16	Midn - midnolin; May be involved in regulation of genes related to neurogenesis in the nucleolus
Gpx3	0.00565	2.16	Gpx3 - glutathione peroxidase 3; Protects cells and enzymes from oxidative damage, by catalyzing the reduction of hydrogen peroxide, lipid peroxides and organic hydroperoxide, by glutathione
Fam46a	0.03494	2.09	Fam46a - family with sequence similarity 46, member A
Irs2	0.03553	2.07	Irs2 - insulin receptor substrate 2; May mediate the control of various cellular processes by insulin
Pth1r	0.00666	2.04	Pth1r - parathyroid hormone 1 receptor; This is a receptor for parathyroid hormone and for parathyroid hormone-related peptide. The activity of this receptor is mediated by G proteins which activate adenylyl cyclase and also a phosphatidylinositol-calcium second messenger system
Arl4d	0.01874	2.01	Arl4d - ADP-ribosylation factor-like 4D; Small GTP-binding protein which cycles between an inactive GDP-bound and an active GTP-bound form, and the rate of cycling is regulated by guanine nucleotide exchange factors (GEF) and GTPase-activating proteins (GAP). GTP-binding protein that does not act as an allosteric

			activator of the cholera toxin catalytic subunit. Recruits CYTH1, CYTH2, CYTH3 and CYTH4 to the plasma membrane in GDP-bound form (By similarity)
Dusp10	0.03712	1.99	Dusp10 - dual specificity phosphatase 10; Protein phosphatase involved in the inactivation of MAP kinases. Has a specificity for the MAPK11/MAPK12/MAPK13/MAPK14 subfamily (By similarity)
Angptl4	0.01906	1.97	Angptl4 - angiopoietin-like 4; Protein with hypoxia-induced expression in endothelial cells. May act as a regulator of angiogenesis and modulate tumorigenesis. Inhibits proliferation, migration, and tubule formation of endothelial cells and reduces vascular leakage. May exert a protective function on endothelial cells through an endocrine action. It is directly involved in regulating glucose homeostasis, lipid metabolism, and insulin sensitivity (By similarity). In response to hypoxia, the unprocessed form of the protein accumulates in the subendothelial extracellular matrix (ECM).
Plau	0.01902	1.96	Plau - plasminogen activator, urokinase; Specifically cleaves the zymogen plasminogen to form the active enzyme plasmin
Txndc11	0.04382	1.95	Txndc11 - thioredoxin domain containing 11; May act as a redox regulator involved in DUOX proteins folding. The interaction with DUOX1 and DUOX2 suggest that it belongs to a multiprotein complex constituting the thyroid H(2)O(2) generating system. It is however not sufficient to assist DUOX1 and DUOX2 in H(2)O(2) generation (By similarity)
Impdh2	0.03297	1.93	Impdh2 - inosine 5'-phosphate dehydrogenase 2; Catalyzes the conversion of inosine 5'-phosphate (IMP) to xanthosine 5'-phosphate (XMP), the first committed and rate- limiting step in the de novo synthesis of guanine nucleotides, and therefore plays an important role in the regulation of cell growth. Could also have a single-stranded nucleic acid-binding activity and could play a role in RNA and/or DNA metabolism (By similarity). It may also have a role in the development of malignancy and the growth progression of some tumors
Cd248	0.03303	1.91	Cd248 - CD248 antigen, endosialin; May play a role in angiogenesis or vascular function
Tmem254b	0.00829	1.90	Gm9746 - predicted gene 9746

Mknk2	0.02374	1.90	Mknk2 - MAP kinase-interacting serine/threonine kinase 2; Serine/threonine-protein kinase that phosphorylates SFPQ/PSF, HNRNPA1 and EIF4E. May play a role in the response to environmental stress and cytokines. Appears to regulate translation by phosphorylating EIF4E, thus increasing the affinity of this protein for the 7-methylguanosine-containing mRNA cap. Required for mediating PP2A-inhibition-induced EIF4E phosphorylation. Triggers EIF4E shuttling from cytoplasm to nucleus.
Tuba4a	0.03758	1.90	Tuba4a - tubulin, alpha 4A; Tubulin is the major constituent of microtubules. It binds two moles of GTP, one at an exchangeable site on the beta chain and one at a non-exchangeable site on the alpha chain
Lama4	0.04189	1.85	Lama4 - laminin, alpha 4; Binding to cells via a high affinity receptor, laminin is thought to mediate the attachment, migration and organization of cells into tissues during embryonic development by interacting with other extracellular matrix components
Orm3	0.02010	1.84	Orm3 - orosomucoid 3; Functions as transport protein in the blood stream. Binds various ligands in the interior of its beta-barrel domain (By similarity). Appears to function in modulating the activity of the immune system during the acute-phase reaction
Pcsk6	0.02599	1.83	Pcsk6 - proprotein convertase subtilisin/kexin type 6
Trib1	0.03565	1.82	Trib1 - tribbles homolog 1 (Drosophila); Interacts with MAPK kinases and regulates activation of MAP kinases. May not display kinase activity (By similarity)
Kcne4	0.03036	1.81	Kcne4 - potassium voltage-gated channel, Isk-related subfamily, gene 4; Ancillary protein that assembles as a beta subunit with a voltage-gated potassium channel complex of pore-forming alpha subunits. Modulates the gating kinetics and enhances stability of the channel complex. Associated with KCNQ1/KVLTQ1 inhibits potassium current (By similarity)
Mpzl2	0.03997	1.79	Mpzl2 - myelin protein zero-like 2; Mediates homophilic cell-cell adhesion
Ptges	0.03043	1.78	Ptges - prostaglandin E synthase; Catalyzes the oxidoreduction of prostaglandin endoperoxide H2 (PGH2) to prostaglandin E2 (PGE2) (By similarity)
Lrrc32	0.01909	1.78	Lrrc32 - leucine rich repeat containing 32
Serpinh1	0.00997	1.78	Serpinh1 - serine (or cysteine) peptidase inhibitor, clade H, member 1; Binds specifically to collagen. Could be involved as a chaperone in the biosynthetic pathway of collagen

Sh3pxd2a	0.00314	1.77	Sh3pxd2a - SH3 and PX domains 2A; Adapter protein involved in invadopodia and podosome formation, extracellular matrix degradation and invasiveness of some cancer cells. Binds matrix metalloproteinases (ADAMs), NADPH oxidases (NOXs) and phosphoinositides. Acts as an organizer protein that allows NOX1- or NOX3-dependent reactive oxygen species (ROS) generation and ROS localization. In association with ADAM12, mediates the neurotoxic effect of beta-amyloid peptide (By similarity)
Gypc	0.03068	1.76	Gypc - glycophorin C
Metrnl	0.03327	1.75	Metrnl - meteorin, glial cell differentiation regulator-like
Gm9938	0.00095	1.72	Gm9938 - predicted gene 9938
Pianp	0.00585	1.72	Pianp - PILR alpha associated neural protein; Acts as a ligand for PILRA in neuronal tissues, where it may be involved in immune regulation
Ubc	0.01569	1.71	Ubc - ubiquitin C
Hsd11b1	0.03662	1.71	Hsd11b1 - hydroxysteroid 11-beta dehydrogenase 1; Catalyzes reversibly the conversion of cortisol to the inactive metabolite cortisone
Mcam	0.01085	1.69	Mcam - melanoma cell adhesion molecule; Plays a role in cell adhesion, and in cohesion of the endothelial monolayer at intercellular junctions in vascular tissue. Its expression may allow melanoma cells to interact with cellular elements of the vascular system, thereby enhancing hematogeneous tumor spread. Could be an adhesion molecule active in neural crest cells during embryonic development. Acts as surface receptor that triggers tyrosine phosphorylation of FYN and PTK2/FAK1, and a transient increase in the intracellular calcium concentration (By similarity)
Ntrk2	0.02251	1.69	Ntrk2 - neurotrophic tyrosine kinase, receptor, type 2; Receptor tyrosine kinase involved in the development and the maturation of the central and the peripheral nervous systems through regulation of neuron survival, proliferation, migration, differentiation, and synapse formation and plasticity. Receptor for BDNF/brain-derived neurotrophic factor and NTF4/neurotrophin- 4. Alternatively can also bind NTF3/neurotrophin-3 which is less efficient in activating the receptor but regulates neuron survival through NTRK2.
Myl9	0.02258	1.67	Myl9 - myosin, light polypeptide 9, regulatory; Myosin regulatory subunit that plays an important role in regulation of both smooth muscle and nonmuscle cell contractile activity via its phosphorylation. Implicated in cytokinesis, receptor capping, and cell

			locomotion (By similarity)
Sardh	0.03418	1.67	Sardh - sarcosine dehydrogenase
Mgmt	0.02327	1.66	Mgmt - O-6-methylguanine-DNA methyltransferase; Involved in the cellular defense against the biological effects of O6-methylguanine (O6-MeG) in DNA. Repairs alkylated guanine in DNA by stoichiometrically transferring the alkyl group at the O-6 position to a cysteine residue in the enzyme. This is a suicide reaction: the enzyme is irreversibly inactivated
Mid1	0.02659	1.66	Mid1 - midline 1; Has E3 ubiquitin ligase activity towards IGBP1, promoting its monoubiquitination, which results in deprotection of the catalytic subunit of protein phosphatase PP2A, and its subsequent degradation by polyubiquitination (By similarity)
Gm8226	0.00905	1.66	Predicted gene 8226
Hspb8	0.01424	1.65	Hspb8 - heat shock protein 8; Displays temperature-dependent chaperone activity
Zfp36l2	0.01782	1.65	Zfp36l2 - zinc finger protein 36, C3H type-like 2; Probable regulatory protein involved in regulating the response to growth factors. RNA-binding protein that binds to 5'UUAUUUAUUU-3' core sequence. Binds to the class II AU-rich element (ARE) in the 3'-UTR of target mRNAs and promotes their deadenylation and degradation (By similarity)
Gadd45g	0.03704	1.65	Gadd45g - growth arrest and DNA-damage-inducible 45 gamma; Involved in the regulation of growth and apoptosis. Mediates activation of stress-responsive MTK1/MEKK4 MAPKKK
Sorcs2	0.01443	1.65	Sorcs2 - sortilin-related VPS10 domain containing receptor 2
Prr5	0.00439	1.64	Prr5 - proline rich 5 (renal); Subunit of mTORC2, which regulates cell growth and survival in response to hormonal signals. mTORC2 is activated by growth factors, but, in contrast to mTORC1, seems to be nutrient-insensitive. mTORC2 seems to function upstream of Rho GTPases to regulate the actin cytoskeleton, probably by activating one or more Rho-type guanine nucleotide exchange factors. mTORC2 promotes the serum-induced formation of stress-fibers or F-actin. mTORC2 plays a critical role in AKT1 'Ser-473' phosphorylation, which may facilitate the phosphorylation of the activation loop of AKT
Spry2	0.01676	1.63	Spry2 - sprouty homolog 2 (Drosophila); May function as an antagonist of fibroblast growth factor (FGF) pathways and may negatively modulate respiratory organogenesis

Cryab	0.01329	1.63	Cryab - crystallin, alpha B; May contribute to the transparency and refractive index of the lens. Has chaperone-like activity, preventing aggregation of various proteins under a wide range of stress conditions
Atp5d	0.03944	1.62	Atp5d - ATP synthase, H+ transporting, mitochondrial F1 complex, delta subunit; Mitochondrial membrane ATP synthase (F(1)F(0) ATP synthase or Complex V) produces ATP from ADP in the presence of a proton gradient across the membrane which is generated by electron transport complexes of the respiratory chain. F-type ATPases consist of two structural domains, F(1) - containing the extramembraneous catalytic core, and F(0) - containing the membrane proton channel, linked together by a central stalk and a peripheral stalk.
Vwa1	0.01459	1.60	Vwa1 - von Willebrand factor A domain containing 1; Promotes matrix assembly
Galnt2	0.00222	1.60	Galnt2 - UDP-N-acetyl-alpha-D-galactosamine:polypeptide N-acetylgalactosaminyltransferase 2; Catalyzes the initial reaction in O-linked oligosaccharide biosynthesis, the transfer of an N-acetyl-D-galactosamine residue to a serine or threonine residue on the protein receptor. Has a broad spectrum of substrates for peptides such as EA2, Muc5AC, Muc1a, Muc1b. Probably involved in O-linked glycosylation of the immunoglobulin A1 (IgA1) hinge region (By similarity)
Des	0.00892	1.60	Des - desmin; Desmin are class-III intermediate filaments found in muscle cells. In adult striated muscle they form a fibrous network connecting myofibrils to each other and to the plasma membrane from the periphery of the Z-line structures
Gm21685	0.00002	1.59	Lysal1 - Ectonucleoside triphosphate diphosphohydrolase 4 ; Hydrolyzes preferentially nucleoside 5'-diphosphates, nucleoside 5'-triphosphates are hydrolyzed only to a minor extent (By similarity)
Tob2	0.04631	1.59	Tob2 - transducer of ERBB2, 2
Isg20	0.01599	1.53	Isg20 - interferon-stimulated protein; Exonuclease with specificity for single-stranded RNA and, to a lesser extent for DNA. Degrades RNA at a rate that is approximately 35-fold higher than its rate for single-stranded DNA. May be involved in the antiviral function of IFN against RNA viruses (By similarity)
Sparcl1	0.00836	1.53	Sparcl1 - SPARC-like 1
Rps4l	0.01425	1.52	Ribosomal protein S4-like

Cldn5	0.01595	1.51	Cldn5 - claudin 5; Plays a major role in tight junction-specific obliteration of the intercellular space, through calcium- independent cell-adhesion activity (By similarity)
Gm14306	0.00421	1.51	Gm14306 - predicted gene 14306
Itga1	0.00992	1.50	Itga1 - integrin alpha 1; Integrin alpha-1/beta-1 is a receptor for laminin and collagen. It recognizes the proline-hydroxylated sequence G-F-P-G- E-R in collagen (By similarity)
