# Supplementary material for: NNT is a key regulator of adrenal redox homeostasis and steroidogenesis in male mice
Source: J Endocrinol. 2017 Oct 18;236(1):13–28. doi: 10.1530/JOE-16-0638 (PMC5744559; doi:10.1530/JOE-16-0638)
Supplement: Supporting Table 5 [file erc-236-13-t005.pdf]

**Supplementary Table 5. Differential gene expression between *Nnt*$^{-/-}$ and *Nnt*$^{BAC}$ : Genes downregulated in *Nnt*$^{BAC}$**

| Gene Symbol | p-value | Fold Change | Gene Description |
|---|---|---|---|
| Nr4a2 | 0.02667 | 6.53 | Nr4a2 - nuclear receptor subfamily 4, group A, member 2; Transcriptional regulator which is important for the differentiation and maintenance of meso-diencephalic dopaminergic (mdDA) neurons during development. It is crucial for expression of a set of genes such as SLC6A3, SLC18A2, TH and DRD2 which are essential for development of mdDA neurons |
| Cyr61 | 0.03506 | 5.73 | Cyr61 - cysteine rich protein 61; Promotes cell proliferation, chemotaxis, angiogenesis and cell adhesion. Appears to play a role in wound healing by up- regulating, in skin fibroblasts, the expression of a number of genes involved in angiogenesis, inflammation and matrix remodeling including VEGA-A, VEGA-C, MMP1, MMP3, TIMP1, uPA, PAI-1 and integrins alpha-3 and alpha-5 (By similarity). CYR61-mediated gene regulation is dependent on heparin-binding (By similarity). Down- regulates the expression of alpha-1 and alpha-2 subunits of collagen type-1 (By similarity). Promotes cell adhesion and adh [...] |
| Ier3 | 0.02278 | 5.66 | Ier3 - immediate early response 3; May play a role in the ERK signaling pathway by inhibiting the dephosphorylation of ERK by phosphatase PP2A-PPP2R5C holoenzyme. Acts also as an ERK downstream effector mediating survival (By similarity). As a member of the NUPR1/RELB/IER3 survival pathway, may allow the development of pancreatic intraepithelial neoplasias |
| Dnajb1 | 0.04696 | 5.30 | Dnajb1 - DnaJ (Hsp40) homolog, subfamily B, member 1; Interacts with HSP70 and can stimulate its ATPase activity. Stimulates the association between HSC70 and HIP (By similarity) |
| Egr1 | 0.01649 | 4.57 | Egr1 - early growth response 1; Transcriptional regulator. Recognizes and binds to the DNA sequence 5'-CGCCCCCGC-3'(EGR-site). Activates the transcription of target genes whose products are required for mitogenesis and differentiation |
| Btg2 | 0.04755 | 4.09 | Btg2 - B cell translocation gene 2, anti-proliferative; Anti-proliferative protein; the function is mediated by association with deadenylase subunits of the CCR4-NOT complex. Activates mRNA deadenyltion in a CNOT6 and CNOT7-dependent manner. In vitro can inhibit deadenylase activity of CNOT7 and CNOT8. Involved in cell cycle regulation. Could be involved in the growth arrest and differentiation of the neuronal precursors. Modulates transcription regulation mediated by ESR1. Involved in mitochondrial depolarization and |

| | | | |
|---|---|---|---|
| | | | neurite outgrowth (By similarity) |
| Junb | 0.0430 9 | 4.00 | Junb - Jun-B oncogene; Transcription factor involved in regulating gene activity following the primary growth factor response. Binds to the DNA sequence 5'-TGA[CG]TCA-3' |
| Ier2 | 0.0348 7 | 3.88 | Ier2 - immediate early response 2 |
| Hbb-bt | 0.0394 7 | 3.53 | Hemoglobin, beta adult t chain |
| Zfp36 | 0.0404 9 | 3.33 | Zfp36 - zinc finger protein 36; mRNA-binding protein involved in post-transcriptional regulation of AU-rich element (ARE)-containing mRNAs. Acts by specifically binding ARE-containing mRNAs and promoting their degradation. Recruits deadenylase CNOT7 (and probably the CCR4-NOT complex) via association with CNOT1. Plays a key role in the post- transcriptional regulation of tumor necrosis factor (TNF) |
| Csrnp1 | 0.0281 3 | 3.24 | Csrnp1 - cysteine-serine-rich nuclear protein 1; Binds to the consensus sequence 5'-AGAGTG-3' and has transcriptional activator activity. May have a tumor-suppressor function. May play a role in apoptosis |
| Hba-a2 | 0.0282 8 | 3.17 | Hba-a2 - hemoglobin alpha, adult chain 2; Involved in oxygen transport from the lung to the various peripheral tissues (By similarity) |
| Hba-a1 | 0.0291 6 | 3.15 | Hba-a1 - hemoglobin alpha, adult chain 1; Involved in oxygen transport from the lung to the various peripheral tissues (By similarity) |
| Hbb-bs | 0.0307 3 | 3.02 | Hemoglobin, beta adult s chain |
| Dusp1 | 0.0370 9 | 2.74 | Dusp1 - dual specificity phosphatase 1; Dual specificity phosphatase that dephosphorylates MAP kinase MAPK1/ERK2 on both 'Thr-183' and 'Tyr-185', regulating its activity during the meiotic cell cycle |
| Klf4 | 0.0440 3 | 2.55 | Klf4 - Kruppel-like factor 4 (gut); Transcription factor; can act both as activator and as repressor. Binds the 5'-CACCC-3' core sequence. Binds to the promoter region of its own gene and can activate its own transcription. Regulates the expression of key transcription factors during embryonic development. Plays an important role in maintaining embryonic stem cells, and in preventing their differentiation. Required for establishing the barrier function of the skin and for postnatal maturation and maintenance of the ocular surface |
| Cwc25 | 0.0323 0 | 2.33 | Cwc25 - CWC25 spliceosome-associated protein homolog (S. cerevisiae) |

| | | | |
|---|---|---|---|
| Irs2 | 0.0254 8 | 2.26 | Irs2 - insulin receptor substrate 2; May mediate the control of various cellular processes by insulin |
| Ppp1r15a | 0.0492 5 | 2.09 | Ppp1r15a - protein phosphatase 1, regulatory (inhibitor) subunit 15A; Recruits the serine/threonine-protein phosphatase PP1 to dephosphorylate the translation initiation factor eIF-2A/EIF2S1, thereby reversing the shut-off of protein synthesis initiated by stress-inducible kinases and facilitating recovery of cells from stress. Down-regulates the TGF-beta signaling pathway by promoting dephosphorylation of TGFB1 by PP1. May promote apoptosis by inducing TP53 phosphorylation on 'Ser-15'. In case of infection with vesicular stomatitis virus (VSV), impairs viral replication |
| Fam46a | 0.0453 7 | 1.96 | Fam46a - family with sequence similarity 46, member A |
| Arl4d | 0.0239 6 | 1.92 | Arl4d - ADP-ribosylation factor-like 4D; Small GTP-binding protein which cycles between an inactive GDP-bound and an active GTP-bound form, and the rate of cycling is regulated by guanine nucleotide exchange factors (GEF) and GTPase-activating proteins (GAP). GTP-binding protein that does not act as an allosteric activator of the cholera toxin catalytic subunit. Recruits CYTH1, CYTH2, CYTH3 and CYTH4 to the plasma membrane in GDP-bound form (By similarity) |
| Pim3 | 0.0255 0 | 1.87 | Pim3 - proviral integration site 3; Proto-oncogene with serine/threonine kinase activity that can prevent apoptosis and promote cell survival and protein translation. May contribute to tumorigenesis through: the delivery of survival signaling through phosphorylation of BAD which induces release of the anti-apoptotic protein Bcl-X(L), the regulation of cell cycle progression and protein synthesis and by regulation of MYC transcriptional activity. Additionally to this role on tumorigenesis, can also negatively regulate insulin secretion by inhibiting the activation of MAPK1/3 (ERK1/2) |
| Rtl1 | 0.0327 6 | 1.78 | Rtl1 - retrotransposon-like 1; Plays an essential role in capillaries endothelial cells for the maintenance of feto-maternal interface and for development of the placenta |
| Gadd45g | 0.0281 6 | 1.72 | Gadd45g - growth arrest and DNA-damage-inducible 45 gamma; Involved in the regulation of growth and apoptosis. Mediates activation of stress-responsive MTK1/MEKK4 MAPKKK |
| Kdm6b | 0.0091 1 | 1.62 | Kdm6b - KDM1 lysine (K)-specific demethylase 6B; Histone demethylase that specifically demethylates 'Lys- 27' of histone H3, thereby playing a central role in histone code. Demethylates trimethylated and dimethylated H3 'Lys-27'. Plays a central role in regulation of posterior development, by regulating HOX gene expression. Involved in |

| | | | |
|---|---|---|---|
| | | | inflammatory response by participating in macrophage differentiation in case of inflammation by regulating gene expression and macrophage differentiation |
| Zfp36l2 | 0.02332 | 1.60 | Zfp36l2 - zinc finger protein 36, C3H type-like 2; Probable regulatory protein involved in regulating the response to growth factors. RNA-binding protein that binds to 5'UUAUUUAUUU-3' core sequence. Binds to the class II AU-rich element (ARE) in the 3'-UTR of target mRNAs and promotes their deadenylation and degradation (By similarity) |
| Gm26547 | 0.01952 | 1.56 | Predicted gene, 26547 |
