# Supplementary material for: NNT is a key regulator of adrenal redox homeostasis and steroidogenesis in male mice
Source: J Endocrinol. 2017 Oct 18;236(1):13–28. doi: 10.1530/JOE-16-0638 (PMC5744559; doi:10.1530/JOE-16-0638)
Supplement: Supporting Table 6 [file erc-236-13-t006.pdf]

Supplementary Table 6. Differential gene expression between *Nnt*$^{-/-}$ vs *Nnt*^{BAC} : Genes upregulated in *Nnt*^{BAC}

Gene Symbol	p-value	Fold Change	Gene Description
Nnt	0.00000	9.82	Nnt - nicotinamide nucleotide transhydrogenase; The transhydrogenation between NADH and NADP is coupled to respiration and ATP hydrolysis and functions as a proton pump across the membrane. May play a role in reactive oxygen species (ROS) detoxification in the adrenal gland (By similarity)
Vsnl1	0.03421	2.26	Vsnl1 - visinin-like 1; Regulates (in vitro) the inhibition of rhodopsin phosphorylation in a calcium-dependent manner (By similarity)
Id2	0.00133	2.18	Id2 - inhibitor of DNA binding 2; ID (inhibitor of DNA binding) HLH proteins lack a basic DNA-binding domain but are able to form heterodimers with other HLH proteins, thereby inhibiting DNA binding. Inhibits skeletal muscle and cardiac myocyte differentiation
Ccdc160	0.00571	2.17	Ccdc160 - coiled-coil domain containing 160
Sncg	0.00300	2.10	Sncg - synuclein, gamma; Plays a role in neurofilament network integrity. May be involved in modulating axonal architecture during development and in the adult. In vitro, increases the susceptibility of neurofilament-H to calcium-dependent proteases. May also function in modulating the keratin network in skin. Activates the MAPK and Elk-1 signal transduction pathway (By similarity)
Pacsin3	0.00015	1.98	Pacsin3 - protein kinase C and casein kinase substrate in neurons 3; May play a role in endocytosis
Cldn1	0.00091	1.94	Cldn1 - claudin 1; Plays a major role in tight junction-specific obliteration of the intercellular space, through calcium- independent cell-adhesion activity
Cryba4	0.02768	1.88	Cryba4 - crystallin, beta A4; Crystallins are the dominant structural components of the vertebrate eye lens
WI1-49P9.2	0.03348	1.87	Predicted gene 43549
E330017L17Rik	0.00670	1.85	RIKEN cDNA E330017L17 gene
Wfdc17	0.02457	1.84	Wfdc17 - WAP four-disulfide core domain 17
Slamf9	0.00175	1.84	Slamf9 - SLAM family member 9; May play a role in the immune response (By similarity)
Icam2	0.00032	1.84	Icam2 - intercellular adhesion molecule 2; ICAM proteins are ligands for the leukocyte adhesion protein LFA-1 (integrin alpha-L/beta-2). ICAM2 may play a role in lymphocyte recirculation by blocking LFA-1-dependent cell adhesion. It mediates adhesive interactions important for antigen- specific immune

			response, NK-cell mediated clearance, lymphocyte recirculation, and other cellular interactions important for immune response and surveillance
1600002H07Rik	0.00191	1.80	ENSMUSG00000024118 - RIKEN cDNA 1600002H07 gene
Il23r	0.01665	1.79	Il23r - interleukin 23 receptor; Associates with IL12RB1 to form the interleukin-23 receptor. Binds IL23 and mediates T-cells, NK cells and possibly certain macrophage/myeloid cells stimulation probably through activation of the Jak-Stat signaling cascade. IL23 functions in innate and adaptive immunity and may participate in acute response to infection in peripheral tissues. IL23 may be responsible for autoimmune inflammatory diseases and be important for tumorigenesis (By similarity)
Zkscan3	0.00003	1.78	Zkscan3 - zinc finger with KRAB and SCAN domains 3; Transcriptional factor that binds to the consensus sequence 5'-[GT][AG][AGT]GGGG-3' and acts as a repressor of autophagy. Specifically represses expression of genes involved in autophagy and lysosome biogenesis/function such as MAP1LC3B, ULK1 or WIPI2. Associates with chromatin at the ITGB4 and VEGF promoters (By similarity)
Gulp1	0.00434	1.77	Gulp1 - GULP, engulfment adaptor PTB domain containing 1; Modulates cellular glycosphingolipid and cholesterol transport. May play a role in the internalization of various LRP1 ligands, such as PSAP (By similarity). May function as an adapter protein. Required for efficient phagocytosis of apoptotic cells. Increases cellular levels of GTP-bound ARF6
Rmdn2	0.02088	1.77	Fam82a1 - family with sequence similarity 82, member A1
Epn3	0.01610	1.77	Epn3 - epsin 3
Ceacam1	0.00023	1.76	Ceacam1 - carcinoembryonic antigen-related cell adhesion molecule 1; Unknown. In case of murine coronavirus (MHV) infection, serves as receptor for MHV S1 spike glycoprotein
Car2	0.02016	1.75	Car2 - carbonic anhydrase 2; Essential for bone resorption and osteoclast differentiation (By similarity). Reversible hydration of carbon dioxide

Exo5	0.00028	1.74	Exo5 - exonuclease 5; Single-stranded DNA (ssDNA) bidirectional exonuclease involved in DNA repair. Probably involved in DNA repair following ultraviolet (UV) irradiation and interstrand cross-links (ICLs) damage. Has both 5'-3' and 3'-5' exonuclease activities with a strong preference for 5'-ends. Acts as a sliding exonuclease that loads at ssDNA ends and then slides along the ssDNA prior to cutting; however the sliding and the 3'-5' exonuclease activities are abolished upon binding to the replication protein A (RPA) complex that enforces 5'-directionality activity (By similarity)
Il2rg	0.01912	1.73	Il2rg - interleukin 2 receptor, gamma chain; Common subunit for the receptors for a variety of interleukins
A530020G20Rik	0.01791	1.73	RIKEN cDNA A530020G20 gene
Gm8430	0.03658	1.72	Rps27a - ribosomal protein S27A
Lilr4b	0.01066	1.72	Leukocyte immunoglobulin-like receptor, subfamily B, member 4B
Ly96	0.00155	1.71	Ly96 - lymphocyte antigen 96; Cooperates with TLR4 in the innate immune response to bacterial lipopolysaccharide (LPS), and with TLR2 in the response to cell wall components from Gram-positive and Gram-negative bacteria. Enhances TLR4-dependent activation of NF-kappa-B. Cells expressing both MD2 and TLR4, but not TLR4 alone, respond to LPS (By similarity)
Gm6344	0.00296	1.70	Predicted gene 6344
Lpin3	0.01463	1.70	Lpin3 - lipin 3; Regulates fatty acid metabolism. Magnesium-dependent phosphatidate phosphatase enzyme which catalyzes the conversion of phosphatidic acid to diacylglycerol during triglyceride, phosphatidylcholine and phosphatidylethanolamine biosynthesis
Klhl14	0.03530	1.70	Klhl14 - kelch-like 14 (Drosophila)
Cd44	0.02558	1.69	Cd44 - CD44 antigen
H2-Aa	0.02808	1.68	H2-Aa - histocompatibility 2, class II antigen A, alpha
Cd48	0.01946	1.68	Cd48 - CD48 antigen; Ligand for CD2. Might facilitate interaction between activated lymphocytes. Probably involved in regulating T-cell activation
Zfp825	0.00019	1.68	Zfp825 - zinc finger protein 825
Srp54a	0.01259	1.66	Srp54a - signal recognition particle 54A
B930041F14Rik	0.00685	1.66	MNCb-3966 - RIKEN cDNA B930041F14 gene
Cd52	0.03681	1.66	Cd52 - CD52 antigen; May play a role in carrying and orienting carbohydrate, as well as having a more specific role

Trim30d	0.00664	1.65	Trim30d - tripartite motif-containing 30D
Ppcs	0.00290	1.65	Ppcs - phosphopantothenoylcysteine synthetase; Catalyzes the first step in the biosynthesis of coenzyme A from vitamin B5, where cysteine is conjugated to 4'-phosphopantothenate to form 4-phosphopantothenoylcysteine (By similarity)
Ttc30b	0.00409	1.65	Ttc30b - tetratricopeptide repeat domain 30B; Required for polyglutamylation of axonemal tubulin (By similarity). Plays a role in anterograde intraflagellar transport (IFT), the process by which cilia precursors are transported from the base of the cilium to the site of their incorporation at the tip (By similarity)
Bcl2l12	0.00162	1.64	Bcl2l12 - BCL2-like 12 (proline rich)
Gins2	0.03317	1.64	Gins2 - GINS complex subunit 2 (Psf2 homolog); The GINS complex plays an essential role in the initiation of DNA replication, and progression of DNA replication forks. GINS complex seems to bind preferentially to single- stranded DNA (By similarity)
Mbip	0.00172	1.64	Mbip - MAP3K12 binding inhibitory protein 1; Inhibits the MAP3K12 activity to induce the activation of the JNK/SAPK pathway (By similarity). Component of the ATAC complex, a complex with histone acetyltransferase activity on histones H3 and H4 (By similarity)
Steap1	0.02697	1.64	Steap1 - six transmembrane epithelial antigen of the prostate 1; Metalloreductase that has the ability to reduce both Fe(3+) to Fe(2+) and Cu(2+) to Cu(1+). Uses NAD(+) as acceptor
Cyp21a2-ps	0.04584	1.63	Cyp21a1 - cytochrome P450, family 21, subfamily a, polypeptide 1; Specifically catalyzes the 21-hydroxylation of steroids. Required for the adrenal synthesis of mineralocorticoids and glucocorticoids
Ctxn3	0.02637	1.63	Ctxn3 - cortexin 3
Lipa	0.01893	1.63	Lipa - lysosomal acid lipase A; Crucial for the intracellular hydrolysis of cholesteryl esters and triglycerides that have been internalized via receptor- mediated endocytosis of lipoprotein particles. Important in mediating the effect of LDL (low density lipoprotein) uptake on suppression of hydroxymethylglutaryl-CoA reductase and activation of endogenous cellular cholesteryl ester formation (By similarity)
Bcl2a1b	0.02788	1.63	Bcl2a1b - B cell leukemia/lymphoma 2 related protein A1b

Akip1	0.01256	1.62	Akip1 - A kinase (PRKA) interacting protein 1; Enhances NF-kappa-B transcriptional activity by regulating the nuclear localization of the NF-kappa-B subunit RELA and promoting the phosphorylation of RELA by PRKACA. Regulates the effect of the cAMP-dependent protein kinase signaling pathway on the NF-kappa-B activation cascade (By similarity)
C1qtnf6	0.01027	1.62	C1qtnf6 - C1q and tumor necrosis factor related protein 6
Ccdc122	0.00305	1.62	Ccdc122 - coiled-coil domain containing 122
Znf41-ps	0.00771	1.62	Gm13139 - predicted gene 13139
Rpl28-ps1	0.02285	1.62	Ribosomal protein L28, pseudogene 1
Pdlim2	0.00608	1.62	Pdlim2 - PDZ and LIM domain 2; Probable adapter protein located at the actin cytoskeleton that promotes cell attachment. Necessary for the migratory capacity of epithelial cells. Overexpression enhances cell adhesion to collagen and fibronectin and suppresses anchorage independent growth. May contribute to tumor cell migratory capacity (By similarity)
Mphosph6	0.01302	1.61	Mphosph6 - M phase phosphoprotein 6; RNA-binding protein that associates with the RNA exosome complex. Involved in the 3'processing of the 7S pre-RNA to the mature 5.8S rRNA and may play a role in recruiting the RNA exosome complex to pre-rRNA; this function may include C1D (By similarity)
Kcnn2	0.01651	1.61	Kcnn2 - potassium intermediate/small conductance calcium-activated channel, subfamily N, member 2; Forms a voltage-independent potassium channel activated by intracellular calcium. Activation is followed by membrane hyperpolarization. Thought to regulate neuronal excitability by contributing to the slow component of synaptic afterhyperpolarization. The channel is blocked by apamin (By similarity)
Trim12c	0.00753	1.61	Trim12c - tripartite motif-containing 12C
Ormdl1	0.00257	1.61	Ormdl1 - ORM1-like 1 (S. cerevisiae); Negative regulator of sphingolipid synthesis (By similarity)
S1pr2	0.00333	1.61	S1pr2 - sphingosine-1-phosphate receptor 2; Receptor for the lysosphingolipid sphingosine 1- phosphate (S1P). S1P is a bioactive lysophospholipid that elicits diverse physiological effect on most types of cells and tissues
Pop7	0.00091	1.60	Pop7 - processing of precursor 7, ribonuclease P family, (S. cerevisiae); Component of ribonuclease P, a protein complex that generates mature tRNA

			molecules by cleaving their 5'-ends (By similarity)
Klf5	0.03162	1.60	Klf5 - Kruppel-like factor 5; Transcription factor that binds to GC box promoter elements. Activates the transcription of these genes
Man1a	0.00171	1.60	Man1a - mannosidase 1, alpha; Involved in the maturation of Asn-linked oligosaccharides. Progressively trim alpha-1,2-linked mannose residues from Man(9)GlcNAc(2) to produce Man(5)GlcNAc(2)
Mc2r	0.02400	1.59	Mc2r - melanocortin 2 receptor; Receptor for ACTH. This receptor is mediated by G proteins (G(s)) which activate adenylate cyclase
Slc35b3	0.00299	1.59	Slc35b3 - solute carrier family 35, member B3; Mediates the transport of adenosine 3'-phospho 5'- phosphosulfate (PAPS), from cytosol into Golgi. PAPS is a universal sulfuryl donor for sulfation events that take place in the Golgi. Compensates for the insufficient expression of SLC35B2/PAPST1 during the synthesis of sulfated glycoconjugates in the colon (By similarity)
Epb4.1l4aos	0.03712	1.59	erythrocyte membrane protein band 4.1 like 4a, opposite strand
Zfp58	0.00018	1.58	Zfp58 - zinc finger protein 58; May have a role during differentiation processes
Fam58b	0.00158	1.58	Fam58b - family with sequence similarity 58, member B; May have a role in cell proliferation (By similarity)
Trmt5	0.00024	1.58	Trmt5 - TRM5 tRNA methyltransferase 5 homolog (S. cerevisiae); Specifically methylates the N1 position of guanosine-37 in various cytoplasmic and mitochondrial tRNAs. Methylation is not dependent on the nature of the nucleoside 5' of the target nucleoside. This is the first step in the biosynthesis of wybutosine (yW), a modified base adjacent to the anticodon of tRNAs and required for accurate decoding (By similarity)
Thap3	0.01167	1.58	Thap3 - THAP domain containing, apoptosis associated protein 3; Component of a THAP1/THAP3-HCFC1-OGT complex that is required for the regulation of the transcriptional activity of RRM1 (By similarity)
Gnat2	0.02969	1.58	Gnat2 - guanine nucleotide binding protein, alpha transducing 2; Guanine nucleotide-binding proteins (G proteins) are involved as modulators or transducers in various transmembrane signaling systems. Transducin is an amplifier and one of the transducers of a visual impulse that performs the coupling between rhodopsin and cGMP-

			phosphodiesterase
Trim21	0.01901	1.58	Trim21 - tripartite motif-containing 21; E3 ubiquitin-protein ligase whose activity is dependent on E2 enzymes, UBE2D1, UBE2D2, UBE2E1 and UBE2E2. Forms a ubiquitin ligase complex in cooperation with the E2 UBE2D2 that is used not only for the ubiquitination of USP4 and IKBKB but also for its self-ubiquitination. Component of cullin-RING-based SCF (SKP1-CUL1-F-box protein) E3 ubiquitin-protein ligase complexes such as SCF(SKP2)-like complexes. A TRIM21-containing SCF(SKP2)- like complex is shown to mediate ubiquitination of CDKN1B ('Thr-187' phosphorylated-form), thereby promoting its degradat [...]
Htr3a	0.03661	1.57	Htr3a - 5-hydroxytryptamine (serotonin) receptor 3A; This is one of the several different receptors for 5- hydroxytryptamine (serotonin), a biogenic hormone that functions as a neurotransmitter, a hormone, and a mitogen. This receptor is a ligand-gated ion channel, which when activated causes fast, depolarizing responses in neurons. It is a cation-specific, but otherwise relatively nonselective, ion channel
Samd9l	0.01190	1.57	Samd9l - sterile alpha motif domain containing 9-like
2610524H06Rik	0.00906	1.57	ENSMUSG00000092486 - RIKEN cDNA 2610524H06 gene
Napepld	0.02307	1.57	Napepld - N-acyl phosphatidylethanolamine phospholipase D; Hydrolyzes N-acyl-phosphatidylethanolamines (NAPEs) to produce N-acylethanolamines (NAEs) and phosphatidic acid. Responsible for the generation of anandamide (N-arachidonoylethanolamine), the ligand of cannabinoid and vanilloid receptors
Tfpi	0.00607	1.57	Tfpi - tissue factor pathway inhibitor; Inhibits factor X (X(a)) directly and, in a Xa-dependent way, inhibits VIIa/tissue factor activity, presumably by forming a quaternary Xa/LACI/VIIa/TF complex. It possesses an antithrombotic action and also the ability to associate with lipoproteins in plasma (By similarity)
Cnr1	0.01002	1.56	Cnr1 - cannabinoid receptor 1 (brain); Involved in cannabinoid-induced CNS effects. Acts by inhibiting adenylate cyclase. Could be a receptor for anandamide. Inhibits L-type Ca(2+) channel current

Rpp38	0.01737	1.56	Rpp38 - ribonuclease P/MRP 38 subunit (human); Component of ribonuclease P, a protein complex that generates mature tRNA molecules by cleaving their 5'-ends. RPP38 may associate transiently with RNase P RNA as a factor involved in the transport of H1 RNA to the putative site of its assembly in the cell, the nucleolus (By similarity)
Ism1	0.01285	1.56	Ism1 - isthmin 1 homolog (zebrafish)
Pnrc2	0.00081	1.56	Pnrc2 - proline-rich nuclear receptor coactivator 2; Involved in nonsense-mediated mRNA decay (NMD) by acting as a bridge between the mRNA decapping complex and the NMD machinery. May act by targeting the NMD machinery to the P-body and recruiting the decapping machinery to aberrant mRNAs. Required for UPF1/RENT1 localization to the P-body. Also acts as a nuclear receptor coactivator. May play a role in controlling the energy balance between energy storage and energy expenditure (By similarity)
Gpm6a	0.03743	1.56	Gpm6a - glycoprotein m6a; Involved in neuronal differentiation, including differentiation and migration of neuronal stem cells. Plays a role in neuronal plasticity and is involved in neurite and filopodia outgrowth, filopodia motility and probably synapse formation. Gpm6a-induced filopodia formation involves mitogen-activated protein kinase (MAPK) and Src signaling pathways. Conflictingly, PubMed:22162747 reports that induced cellular protrusions are simple membrane-wrapped tubules without actin or tubulin-based cytoskeletons and with Gpm6a gliding along membrane edges indicative for a functio [...]
Cmtm7	0.00539	1.56	Cmtm7 - CKLF-like MARVEL transmembrane domain containing 7
Ms4a7	0.02153	1.56	Ms4a7 - membrane-spanning 4-domains, subfamily A, member 7
Commd5	0.00161	1.56	Commd5 - COMM domain containing 5; Down-regulates activation of NF-kappa-B (By similarity)
4921524J17Rik	0.00654	1.56	ENSMUSG00000036934 - RIKEN cDNA 4921524J17 gene
9330151L19Rik	0.00129	1.56	ENSMUSG00000097061 - Uncharacterized protein
Etv1	0.01983	1.56	Etv1 - ets variant gene 1; Transcriptional activator that binds to DNA sequences containing the consensus pentanucleotide 5'-CGGA[AT]-3'

Pdgfd	0.01305	1.56	Pdgfd - platelet-derived growth factor, D polypeptide; Growth factor that plays an essential role in the regulation of embryonic development, cell proliferation, cell migration, survival and chemotaxis. Potent mitogen for cells of mesenchymal origin. Plays an important role in wound healing (By similarity). Has oncogenic potential and can induce tumor formation. Induces macrophage recruitment, increased interstitial pressure, and blood vessel maturation during angiogenesis. Can initiate events that lead to a mesangial proliferative glomerulonephritis, including influx of monocytes and macropha [...]
Hsd3b1	0.02395	1.55	Hsd3b1 - hydroxy-delta-5-steroid dehydrogenase, 3 beta- and steroid delta-isomerase 1; 3-beta-HSD is a bifunctional enzyme, that catalyzes the oxidative conversion of Delta(5)-ene-3-beta-hydroxy steroid, and the oxidative conversion of ketosteroids. The 3-beta-HSD enzymatic system plays a crucial role in the biosynthesis of all classes of hormonal steroids
Paqr7	0.01350	1.55	Paqr7 - progestin and adipoQ receptor family member VII; Steroid membrane receptor. May be involved in oocyte maturation (By similarity)
Nans	0.00244	1.55	Nans - N-acetylneuraminic acid synthase (sialic acid synthase)
Pdlim4	0.01289	1.55	Pdlim4 - PDZ and LIM domain 4
Sox12	0.01085	1.54	Sox12 - SRY-box containing gene 12; Binds to the sequence 5'-AACAAT-3' (By similarity)
Pkia	0.00152	1.54	Pkia - protein kinase inhibitor, alpha; Extremely potent competitive inhibitor of cAMP-dependent protein kinase activity, this protein interacts with the catalytic subunit of the enzyme after the cAMP-induced dissociation of its regulatory chains
Tmem159	0.03255	1.54	Tmem159 - transmembrane protein 159
Zfp280b	0.00194	1.54	Zfp280b - zinc finger protein 280B
Znhit2	0.00114	1.54	Znhit2 - zinc finger, HIT domain containing 2
Omd	0.01732	1.54	Omd - osteomodulin; May be implicated in biomineralization processes. Has a function in binding of osteoblasts via the alpha(V)beta(3)-integrin (By similarity)

Cenpt	0.00596	1.54	Cenpt - centromere protein T; Component of the CENPA-NAC (nucleosome-associated) complex, a complex that plays a central role in assembly of kinetochore proteins, mitotic progression and chromosome segregation. The CENPA-NAC complex recruits the CENPA-CAD (nucleosome distal) complex and may be involved in incorporation of newly synthesized CENPA into centromeres. Part of a nucleosome- associated complex that binds specifically to histone H3- containing nucleosomes at the centromere, as opposed to nucleosomes containing CENPA. Component of the heterotetrameric CENP-T-W-S-X complex that binds an [...]
Adprhl2	0.00013	1.54	Adprhl2 - ADP-ribosylhydrolase like 2; Poly(ADP-ribose) synthesized after DNA damage is only present transiently and is rapidly degraded by poly(ADP-ribose) glycohydrolase. Poly(ADP-ribose) metabolism may be required for maintenance of the normal function of neuronal cells. Generates ADP-ribose from poly-(ADP-ribose), but does not hydrolyze ADP- ribose-arginine, -cysteine, -diphthamide, or -asparagine bonds. Due to catalytic inactivity of PARG mitochondrial isoforms, ARH3 is the only PAR hydrolyzing enzyme in mitochondria (By similarity)
1500015O10Rik	0.04656	1.54	Ecrg4 - RIKEN cDNA 1500015O10 gene; Probable hormone that may induce senescence of oligodendrocyte and neural precursor cells, characterized by G1 arrest, RB1 dephosphorylation and accelerated CCND1 and CCND3 proteasomal degradation
Sfrp5	0.04547	1.54	Sfrp5 - secreted frizzled-related sequence protein 5; Soluble frizzled-related proteins (sFRPS) function as modulators of Wnt signaling through direct interaction with Wnts. They have a role in regulating cell growth and differentiation in specific cell types. SFRP5 may be involved in determining the polarity of photoreceptor, and perhaps, other cells in the retina
Alox5ap	0.02535	1.54	Alox5ap - arachidonate 5-lipoxygenase activating protein; Required for leukotriene biosynthesis by ALOX5 (5- lipoxygenase). Anchors ALOX5 to the membrane. Binds arachidonic acid, and could play an essential role in the transfer of arachidonic acid to ALOX5. Binds to MK-886, a compound that blocks the biosynthesis of leukotrienes (By similarity). unstructured (By similarity)
Lum	0.02881	1.54	Lum - lumican
Osgin1	0.01416	1.54	Osgin1 - oxidative stress induced growth

			inhibitor 1
Nup35	0.00078	1.53	Nup35 - nucleoporin 35; Functions as a component of the nuclear pore complex (NPC). NPC components, collectively referred to as nucleoporins (NUPs), can play the role of both NPC structural components and of docking or interaction partners for transiently associated nuclear transport factors. May play a role in the association of MAD1 with the NPC (By similarity)
Gemin6	0.00047	1.53	Gemin6 - gem (nuclear organelle) associated protein 6; The SMN complex plays an essential role in spliceosomal snRNP assembly in the cytoplasm and is required for pre-mRNA splicing in the nucleus
Cd300lh	0.01961	1.53	Cd300lh - CD300 antigen like family member H; Acts as an activating receptor inducing cytokine production in mast cells. Can act as a positive regulator of TLR9 signaling in macrophages, leading to enhanced production of proinflammatory cytokines
Arhgdib	0.00242	1.53	Arhgdib - Rho, GDP dissociation inhibitor (GDI) beta; Regulates the GDP/GTP exchange reaction of the Rho proteins by inhibiting the dissociation of GDP from them, and the subsequent binding of GTP to them
Ska2	0.00102	1.53	Ska2 - spindle and kinetochore associated complex subunit 2; Component of the SKA1 complex, a microtubule-binding subcomplex of the outer kinetochore that is essential for proper chromosome segregation. Required for timely anaphase onset during mitosis, when chromosomes undergo bipolar attachment on spindle microtubules leading to silencing of the spindle checkpoint. The SKA1 complex is a direct component of the kinetochore-microtubule interface and directly associates with microtubules as oligomeric assemblies. The complex facilitates the processive movement of microspheres along a microtubu [...]
Zfp580	0.01289	1.53	Zfp580 - zinc finger protein 580; May be involved in transcriptional regulation
Haghl	0.00450	1.53	Haghl - hydroxyacylglutathione hydrolase-like; Hydrolase acting on ester bonds (Potential)
Snupn	0.01165	1.52	Snupn - snurportin 1; Functions as an U snRNP-specific nuclear import adapter. Involved in the trimethylguanosine (m3G)-cap-dependent nuclear import of U snRNPs. Binds specifically to the terminal m3G-cap U snRNAs (By similarity)

Nudt16	0.00475	1.52	Nudt16 - nudix (nucleoside diphosphate linked moiety X)-type motif 16; RNA-binding and decapping enzyme that catalyzes the cleavage of the cap structure of snoRNAs and mRNAs in a metal- dependent manner. Part of the U8 snoRNP complex that is required for the accumulation of mature 5.8S and 28S rRNA. Has diphosphatase activity and removes m7G and/or m227G caps from U8 snoRNA and leaves a 5'monophosphate on the RNA. Catalyzes also the cleavage of the cap structure on mRNAs. Does not hydrolyze cap analog structures like 7-methylguanosine nucleoside triphosphate (m7GpppG). Also hydrolysis m7G- and [...]
Ccdc8	0.04001	1.52	Ccdc8 - coiled-coil domain containing 8
Rangrf	0.02071	1.52	Rangrf - RAN guanine nucleotide release factor; May regulate the intracellular trafficking of RAN. In cardiac cells seems to regulate the cell surface localization of SCN5A
Cd53	0.04923	1.52	Cd53 - CD53 antigen; May be involved in growth regulation in hematopoietic cells
Ipp	0.00004	1.52	Ipp - IAP promoted placental gene; May play a role in organizing the actin cytoskeleton
Fam105a	0.01302	1.51	Fam105a - family with sequence similarity 105, member A
Frk	0.03149	1.51	Frk - fyn-related kinase; Non-receptor tyrosine-protein kinase that negatively regulates cell proliferation. Positively regulates PTEN protein stability through phosphorylation of PTEN on 'Tyr-336', which in turn prevents its ubiquitination and degradation, possibly by reducing its binding to NEDD4. May function as a tumor suppressor (By similarity)
Cdc42ep3	0.01588	1.51	Cdc42ep3 - CDC42 effector protein (Rho GTPase binding) 3; Probably involved in the organization of the actin cytoskeleton. May act downstream of CDC42 to induce actin filament assembly leading to cell shape changes. Induces pseudopodia formation in fibroblasts (By similarity)
Oxr1	0.00471	1.51	Oxr1 - oxidation resistance 1; May be involved in protection from oxidative damage
Nufip1	0.00138	1.51	Nufip1 - nuclear fragile X mental retardation protein interacting protein 1; Binds RNA
Dus4l	0.01846	1.51	Dus4l - dihydrouridine synthase 4-like (S. cerevisiae); Catalyzes the synthesis of dihydrouridine, a modified base found in the D-loop of most tRNAs (By similarity)

Ddx58	0.01335	1.51	Ddx58 - DEAD (Asp-Glu-Ala-Asp) box polypeptide 58; Innate immune receptor which acts as a cytoplasmic sensor of viral nucleic acids and plays a major role in sensing viral infection and in the activation of a cascade of antiviral responses including the induction of type I interferons and proinflammatory cytokines. Its ligands include: 5'-triphosphorylated ssRNA and dsRNA and short dsRNA (<1 kb in length). In addition to the 5'-triphosphate moiety, blunt-end base pairing at the 5'-end of the RNA is very essential. Overhangs at the non-triphosphorylated end of the dsRNA RNA have no major impac [...]
Trim34a	0.04171	1.51	Trim34a - tripartite motif-containing 34A
Tuft1	0.00588	1.51	Tuft1 - tuftelin 1; Involved in the mineralization and structural organization of enamel
Pigh	0.00072	1.51	Pigh - phosphatidylinositol glycan anchor biosynthesis, class H; Part of the complex catalyzing the transfer of N-acetylglucosamine from UDP-N-acetylglucosamine to phosphatidylinositol, the first step of GPI biosynthesis (By similarity)
Zfp958	0.01237	1.50	Zfp958 - zinc finger protein 958
Hao2	0.00106	1.50	Hao2 - hydroxyacid oxidase 2; Has 2-hydroxyacid oxidase activity. Most active on medium-chain substrates
