# Supplementary material for: NNT is a key regulator of adrenal redox homeostasis and steroidogenesis in male mice
Source: J Endocrinol. 2017 Oct 18;236(1):13–28. doi: 10.1530/JOE-16-0638 (PMC5744559; doi:10.1530/JOE-16-0638)
Supplement: Supporting Table 7 [file erc-236-13-t007.pdf]

Supplementary Table 7. Differential gene expression between *Nnt*$^{+/+}$ and *Nnt*^{BAC} : Genes downregulated in *Nnt*^{BAC}

Gene Symbol	p-value	Fold Change	Gene Description
Wdfy1	0.00000	2.54	Wdfy1 - WD repeat and FYVE domain containing 1
RP24-378G4.3	0.00004	2.34	predicted gene 43305, known TEC
Cml5	0.00055	2.15	Cml5 - camello-like 5; May play a role in regulation of gastrulation
Nat8	0.00044	2.04	Nat8 - N-acetyltransferase 8 (GCN5-related, putative); Plays a role in regulation of gastrulation
Ppl	0.00015	1.85	Ppl - periplakin; Component of the cornified envelope of keratinocytes. May link the cornified envelope to desmosomes and intermediate filaments. May act as a localization signal in PKB/AKT-mediated signaling
Rmrp	0.02272	1.81	RNA component of mitochondrial RNA processing endoribonuclease
RP23-338P12.2	0.02875	1.80	RIKEN cDNA 7330423F06
Gm26547	0.00319	1.76	Predicted gene, 26547
Malat1	0.00622	1.73	Metastasis associated lung adenocarcinoma transcript 1
RP24-230J14.5	0.00013	1.72	Predicted gene 42427
Gm17275	0.01015	1.71	Predicted gene, 17275
Trank1	0.00264	1.70	Trank1 - tetratricopeptide repeat and ankyrin repeat containing 1
Gm26880	0.00011	1.69	predicted gene, 26880 known lincRNA
Cep85	0.00033	1.64	Ccdc21 - coiled-coil domain containing 21
Raph1	0.00406	1.60	Raph1 - Ras association (RalGDS/AF-6) and pleckstrin homology domains 1
AA986860	0.00028	1.59	AA986860 - expressed sequence AA986860; Putative androgen-specific receptor (By similarity)
A330023F24Rik	0.00481	1.58	RIKEN cDNA A330023F24 gene
Prrg4	0.04403	1.54	Prrg4 - proline rich Gla (G-carboxyglutamic acid) 4 (transmembrane)
Pfkfb2	0.00255	1.53	Pfkfb2 - 6-phosphofructo-2-kinase/fructose-2,6-biphosphatase 2; Synthesis and degradation of fructose 2,6-bisphosphate
Susd3	0.02218	1.51	Susd3 - sushi domain containing 3
Sema5b	0.03605	1.51	Sema5b - sema domain, seven thrombospondin repeats (type 1 and type 1-

			like), transmembrane domain (TM) and short cytoplasmic domain, (semaphorin) 5B; May act as positive axonal guidance cues
Gm28382	0.0411 2	1.50	predicted gene 28382 known lincRNA
