# Supplementary material for: NNT is a key regulator of adrenal redox homeostasis and steroidogenesis in male mice
Source: J Endocrinol. 2017 Oct 18;236(1):13–28. doi: 10.1530/JOE-16-0638 (PMC5744559; doi:10.1530/JOE-16-0638)
Supplement: Supporting Table 8 [file erc-236-13-t008.pdf]

Supplementary Table 8. Differential gene expression between *Nnt*+/+ and *Nnt*^{BAC} : Genes upregulated in Nnt^{BAC}

Gene Symbol	p-value	Fold Change	Gene Description
Igha	0.02643	6.26	Immunoglobulin heavy constant alpha
Igkc	0.02971	4.89	Immunoglobulin kappa constant
Gm9780	0.00000	2.83	Gm10393 - predicted gene 10393
Clec3b	0.04322	2.77	Clec3b - C-type lectin domain family 3, member b; Tetranectin binds to plasminogen and to isolated kringle 4. May be involved in the packaging of molecules destined for exocytosis (By similarity)
Dpt	0.02792	2.72	Dpt - dermatopontin; Seems to mediate adhesion by cell surface integrin binding. May serve as a communication link between the dermal fibroblast cell surface and its extracellular matrix environment. Enhances TGFB1 activity (By similarity). Inhibits cell proliferation. Accelerates collagen fibril formation, and stabilizes collagen fibrils against low-temperature dissociation
Plac9a	0.00004	2.70	Gm10393 - predicted gene 10393
Nnt	0.00002	2.67	Nnt - nicotinamide nucleotide transhydrogenase; The transhydrogenation between NADH and NADP is coupled to respiration and ATP hydrolysis and functions as a proton pump across the membrane. May play a role in reactive oxygen species (ROS) detoxification in the adrenal gland (By similarity)
Cd248	0.00820	2.20	Cd248 - CD248 antigen, endosialin; May play a role in angiogenesis or vascular function
Tmem254b	0.00125	2.20	Gm9746 - predicted gene 9746
Col3a1	0.01621	2.16	Col3a1 - collagen, type III, alpha 1; Collagen type III occurs in most soft connective tissues along with type I collagen
Hmgcs2	0.03288	2.08	Hmgcs2 - 3-hydroxy-3-methylglutaryl-Coenzyme A synthase 2; This enzyme condenses acetyl-CoA with acetoacetyl-CoA to form HMG-CoA, which is the substrate for HMG-CoA reductase
Mid1	0.00174	2.04	Mid1 - midline 1; Has E3 ubiquitin ligase activity towards IGBP1, promoting its monoubiquitination, which results in deprotection of the catalytic subunit of protein phosphatase PP2A, and its subsequent degradation by polyubiquitination (By similarity)
Mmp2	0.02358	2.02	Mmp2 - matrix metallopeptidase 2; Ubiquitinous metalloproteinase that is involved in diverse functions such as remodeling of the vasculature, angiogenesis, tissue repair, tumor invasion, inflammation, and atherosclerotic plaque rupture. As well as degrading extracellular matrix proteins, can also act on

			several nonmatrix proteins such as big endothelial 1 and beta-type CGRP promoting vasoconstriction. Also cleaves KISS at a Gly-|-Leu bond. Appears to have a role in myocardial cell death pathways. Contributes to myocardial oxidative stress by regulating the activity of GSK3beta.
Cartpt	0.02098	1.97	Cartpt - CART prepropeptide; Satiety factor closely associated with the actions of leptin and neuropeptide y; this anorectic peptide inhibits both normal and starvation-induced feeding and completely blocks the feeding response induced by neuropeptide Y and regulated by leptin in the hypothalamus (By similarity)
Gulp1	0.00193	1.97	Gulp1 - GULP, engulfment adaptor PTB domain containing 1; Modulates cellular glycosphingolipid and cholesterol transport. May play a role in the internalization of various LRP1 ligands, such as PSAP (By similarity). May function as an adapter protein. Required for efficient phagocytosis of apoptotic cells. Increases cellular levels of GTP-bound ARF6
Pf4	0.04665	1.96	Pf4 - platelet factor 4; Released during platelet aggregation. Neutralizes the anticoagulant effect of heparin because it binds more strongly to heparin than to the chondroitin-4-sulfate chains of the carrier molecule. Chemotactic for neutrophils and monocytes. Inhibits endothelial cell proliferation (By similarity)
Pcsk6	0.01313	1.95	Pcsk6 - proprotein convertase subtilisin/kexin type 6
Gm2564	0.04951	1.94	Gm2442 - predicted gene 2442
Spp1	0.01725	1.91	Spp1 - secreted phosphoprotein 1; Binds tightly to hydroxyapatite. Appears to form an integral part of the mineralized matrix. Probably important to cell-matrix interaction
Wnt6	0.02776	1.90	Wnt6 - wingless-related MMTV integration site 6; Ligand for members of the frizzled family of seven transmembrane receptors. Probable developmental protein. May be a signaling molecule which affects the development of discrete regions of tissues. Is likely to signal over only few cell diameters
Pdgfra	0.01750	1.89	Pdgfra - platelet derived growth factor receptor, alpha polypeptide; Tyrosine-protein kinase that acts as a cell-surface receptor for PDGFA, PDGFB and PDGFC and plays an essential role in the regulation of embryonic development, cell proliferation, survival and chemotaxis. Depending on the context, promotes or inhibits cell proliferation and cell migration. Plays an important role in the differentiation of bone marrow-derived mesenchymal stem cells. Required for normal

			skeleton development and cephalic closure during embryonic development.
Hoxc8	0.00647	1.88	Hoxc8 - homeobox C8; Sequence-specific transcription factor which is part of a developmental regulatory system that provides cells with specific positional identities on the anterior-posterior axis
Gsn	0.02935	1.87	Gsn - gelsolin; Calcium-regulated, actin-modulating protein that binds to the plus (or barbed) ends of actin monomers or filaments, preventing monomer exchange (end-blocking or capping). It can promote the assembly of monomers into filaments (nucleation) as well as sever filaments already formed. Plays a role in ciliogenesis
Olfml1	0.00100	1.86	Olfml1 - olfactomedin-like 1
Srpx2	0.00361	1.84	Srpx2 - sushi-repeat-containing protein, X-linked 2; Acts as a ligand for the urokinase plasminogen activator surface receptor. Plays a role in angiogenesis by inducing endothelial cell migration and the formation of vascular network (cords). Involved in cellular migration and adhesion in cancer cells. Increases the phosphorylation levels of FAK
C6	0.01225	1.82	C6 - complement component 6
Gm14403	0.01122	1.80	Gm14403 - predicted gene 14403
Igf1	0.03899	1.80	Igf1 - insulin-like growth factor 1; The insulin-like growth factors, isolated from plasma, are structurally and functionally related to insulin but have a much higher growth-promoting activity. May be a physiological regulator of [1-14C]-2-deoxy-D-glucose (2DG) transport and glycogen synthesis in osteoblasts. Stimulates glucose transport in rat bone-derived osteoblastic (PyMS) cells and is effective at much lower concentrations than insulin, not only regarding glycogen and DNA synthesis but also with regard to enhancing glucose uptake (By similarity)
Myl9	0.01018	1.78	Myl9 - myosin, light polypeptide 9, regulatory; Myosin regulatory subunit that plays an important role in regulation of both smooth muscle and nonmuscle cell contractile activity via its phosphorylation. Implicated in cytokinesis, receptor capping, and cell locomotion (By similarity)
Tagln	0.04092	1.78	Tagln - transgelin; Actin cross-linking/gelling protein (By similarity)
Vit	0.00130	1.78	Vit - vitrin; Promotes matrix assembly and cell adhesiveness
Wfdc1	0.02878	1.77	Wfdc1 - WAP four-disulfide core domain 1; Has growth inhibitory activity (By similarity)

Serpinf1	0.02924	1.75	Serpinf1 - serine (or cysteine) peptidase inhibitor, clade F, member 1; Neurotrophic protein; induces extensive neuronal differentiation in retinoblastoma cells. Potent inhibitor of angiogenesis. As it does not undergo the S (stressed) to R (relaxed) conformational transition characteristic of active serpins, it exhibits no serine protease inhibitory activity
Rarres2	0.01058	1.73	Rarres2 - retinoic acid receptor responder (tazarotene induced) 2
Lmcd1	0.02028	1.73	Lmcd1 - LIM and cysteine-rich domains 1; Transcriptional cofactor that restricts GATA6 function by inhibiting DNA-binding, resulting in repression of GATA6 transcriptional activation of downstream target genes. Represses GATA6-mediated trans activation of lung- and cardiac tissue- specific promoters. Inhibits DNA-binding by GATA4 and GATA1 to the cTNC promoter. Plays a critical role in the development of cardiac hypertrophy via activation of calcineurin/nuclear factor of activated T-cells signaling pathway
Igfbp4	0.00139	1.73	Igfbp4 - insulin-like growth factor binding protein 4; IGF-binding proteins prolong the half-life of the IGFs and have been shown to either inhibit or stimulate the growth promoting effects of the IGFs on cell culture. They alter the interaction of IGFs with their cell surface receptors
Gm9938	0.00093	1.73	Gm9938 - predicted gene 9938
Pth1r	0.04127	1.73	Pth1r - parathyroid hormone 1 receptor; This is a receptor for parathyroid hormone and for parathyroid hormone-related peptide. The activity of this receptor is mediated by G proteins which activate adenylyl cyclase and also a phosphatidylinositol-calcium second messenger system
Pianp	0.00581	1.72	Pianp - PILR alpha associated neural protein; Acts as a ligand for PILRA in neuronal tissues, where it may be involved in immune regulation
Cryab	0.00652	1.71	Cryab - crystallin, alpha B; May contribute to the transparency and refractive index of the lens. Has chaperone-like activity, preventing aggregation of various proteins under a wide range of stress conditions
Gm13152	0.00143	1.70	Gm13152 - predicted gene 13152
Gfra2	0.03706	1.70	Gfra2 - glial cell line derived neurotrophic factor family receptor alpha 2; Receptor for neurturin. Mediates the NRTN-induced autophosphorylation and activation of the RET receptor. Also able to mediate GDNF signaling through the RET tyrosine kinase receptor
Gpr153	0.03110	1.70	Gpr153 - G protein-coupled receptor 153;

			Orphan receptor
Loxl1	0.00468	1.70	Loxl1 - lysyl oxidase-like 1; Active on elastin and collagen substrates (By similarity)
Cdkn2b	0.00668	1.69	Cdkn2b - cyclin-dependent kinase inhibitor 2B (p15, inhibits CDK4); Interacts strongly with CDK4 and CDK6. Potent inhibitor. Potential effector of TGF-beta induced cell cycle arrest (By similarity)
Pdgfc	0.00009	1.69	Pdgfc - platelet-derived growth factor, C polypeptide; Growth factor that plays an essential role in the regulation of embryonic development, cell proliferation, cell migration, survival and chemotaxis. Potent mitogen and chemoattractant for cells of mesenchymal origin. Required for normal skeleton formation during embryonic development, especially for normal development of the craniofacial skeleton and for normal development of the palate. Required for normal skin morphogenesis during embryonic development.
Slc6a2	0.01062	1.68	Slc6a2 - solute carrier family 6 (neurotransmitter transporter, noradrenalin), member 2; Amine transporter. Terminates the action of noradrenaline by its high affinity sodium-dependent reuptake into presynaptic terminals
Itga1	0.00141	1.68	Itga1 - integrin alpha 1; Integrin alpha-1/beta-1 is a receptor for laminin and collagen. It recognizes the proline-hydroxylated sequence G-F-P-G- E-R in collagen (By similarity)
Sardh	0.03280	1.67	Sardh - sarcosine dehydrogenase
Fcgr2b	0.01436	1.67	Fcgr2b - Fc receptor, IgG, low affinity IIb
Srpx	0.00114	1.66	Srpx - sushi-repeat-containing protein
S100a11	0.03175	1.66	S100a11 - S100 calcium binding protein A11 (calgizzarin); Facilitates the differentiation and the cornification of keratinocytes (By similarity)
Pdlim4	0.00662	1.66	Pdlim4 - PDZ and LIM domain 4
Sdc1	0.00129	1.66	Sdc1 - syndecan 1; Cell surface proteoglycan that bears both heparan sulfate and chondroitin sulfate and that links the cytoskeleton to the interstitial matrix
Cdh11	0.00861	1.65	Cdh11 - cadherin 11; Cadherins are calcium-dependent cell adhesion proteins. They preferentially interact with themselves in a homophilic manner in connecting cells; cadherins may thus contribute to the sorting of heterogeneous cell types
Rnase4	0.00021	1.65	Rnase4 - ribonuclease, RNase A family 4; This RNase has marked specificity towards the 3' side of uridine nucleotides (By similarity)
Ogn	0.00032	1.65	Ogn - osteoglycin; Induces bone formation in conjunction with TGF-beta-1 or TGF-beta-2 (By similarity)

Zkscan3	0.00008	1.64	Zkscan3 - zinc finger with KRAB and SCAN domains 3; Transcriptional factor that binds to the consensus sequence 5'-[GT][AG][AGT]GGGG-3' and acts as a repressor of autophagy. Specifically represses expression of genes involved in autophagy and lysosome biogenesis/function such as MAP1LC3B, ULK1 or WIPI2. Associates with chromatin at the ITGB4 and VEGF promoters (By similarity)
Klf5	0.02535	1.64	Klf5 - Kruppel-like factor 5; Transcription factor that binds to GC box promoter elements. Activates the transcription of these genes
Scara5	0.00492	1.64	Scara5 - scavenger receptor class A, member 5 (putative); Ferritin receptor that mediates non-transferrin- dependent delivery of iron. Mediates cellular uptake of ferritin- bound iron by stimulating ferritin endocytosis from the cell surface with consequent iron delivery within the cell. Delivery of iron to cells by ferritin is required for the development of specific cell types, suggesting the existence of cell type-specific mechanisms of iron traffic in organogenesis, which alternatively utilize transferrin or non-transferrin iron delivery pathways.
Rspo1	0.03058	1.64	Rspo1 - R-spondin homolog (Xenopus laevis); Activator of the beta-catenin signaling cascade, leading to TCF-dependent gene activation. Acts both in the canonical Wnt/beta-catenin-dependent pathway and in non-canonical Wnt signaling pathway, probably by acting as an inhibitor of ZNRF3, an important regulator of the Wnt signaling pathway. Acts as a ligand for frizzled FZD8 and LRP6. May negatively regulate the TGF-beta pathway. Has a essential roles in ovary determination (By similarity)
Gm14306	0.00078	1.64	Gm14306 - predicted gene 14306
Man1a	0.00127	1.64	Man1a - mannosidase 1, alpha; Involved in the maturation of Asn-linked oligosaccharides. Progressively trim alpha-1,2-linked mannose residues from Man(9)GlcNAc(2) to produce Man(5)GlcNAc(2)
Htra3	0.00145	1.63	Htra3 - HtrA serine peptidase 3; Serine protease that cleaves beta-casein/CSN2 as well as several extracellular matrix (ECM) proteoglycans such as decorin/DCN, biglycan/BGN and fibronectin/FN1. Inhibits signaling mediated by TGF-beta family proteins possibly indirectly by degradation of these ECM proteoglycans. May act as a tumor suppressor. Negatively regulates, in vitro, trophoblast invasion during placental development and may

			be involved in the development of the placenta in vivo. May also have a role in ovarian development, granulosa cell differentiation and luteinization
AC153941.1	0.02681	1.63	Gene AC153941.1
Cyp7b1	0.02274	1.62	Cyp7b1 - cytochrome P450, family 7, subfamily b, polypeptide 1
Dcn	0.01229	1.62	Dcn - decorin; May affect the rate of fibrils formation
Cilp	0.01282	1.61	Cilp - cartilage intermediate layer protein, nucleotide pyrophosphohydrolase; Probably plays a role in cartilage scaffolding. May act by antagonizing TGF-beta1 (TGFB1) and IGF1 functions. Has the ability to suppress IGF1-induced proliferation and sulfated proteoglycan synthesis, and inhibits ligand-induced IGF1R autophosphorylation. May inhibit TGFB1-mediated induction of cartilage matrix genes via its interaction with TGFB1. Overexpression may lead to impair chondrocyte growth and matrix repair and indirectly promote inorganic pyrophosphate (PPi) supersaturation in aging and osteoarthritis
Scx	0.04543	1.61	Scx - scleraxis; Plays an early essential role in mesoderm formation, as well as a later role in formation of somite-derived chondrogenic lineages
Prr5	0.00595	1.61	Prr5 - proline rich 5 (renal); Subunit of mTORC2, which regulates cell growth and survival in response to hormonal signals. mTORC2 is activated by growth factors, but, in contrast to mTORC1, seems to be nutrient-insensitive. mTORC2 seems to function upstream of Rho GTPases to regulate the actin cytoskeleton, probably by activating one or more Rho-type guanine nucleotide exchange factors. mTORC2 promotes the serum-induced formation of stress-fibers or F-actin.
Penk	0.00811	1.61	Penk - preproenkephalin; Met- and Leu-enkephalins compete with and mimic the effects of opiate drugs. They play a role in a number of physiologic functions, including pain perception and responses to stress. PENK(114-133) and PENK(238-259) increase glutamate release in the striatum. PENK(114-133) decreases GABA concentration in the striatum
Lnpep	0.00226	1.60	Lnpep - leucyl/cystinyl aminopeptidase; Release of an N-terminal amino acid, cleave before cysteine, leucine as well as other amino acids. Degrades peptide hormones such as oxytocin, vasopressin and angiotensin III, and plays a role in maintaining homeostasis during pregnancy. May be involved in the inactivation

			of neuronal peptides in the brain. Cleaves Met-enkephalin and dynorphin. Binds angiotensin IV and may be the angiotensin IV receptor in the brain (By similarity)
S100a6	0.03924	1.60	S100a6 - S100 calcium binding protein A6 (calcyclin); May function as calcium sensor and contribute to cellular calcium signaling (Potential). May function by interacting with other proteins and indirectly play a role in the reorganization of the actin cytoskeleton and in cell motility. Binds 2 calcium ions. Calcium binding is cooperative (By similarity). Interacts with FKBP4 (By similarity)
Mfap4	0.01066	1.60	Mfap4 - microfibrillar-associated protein 4; Could be involved in calcium-dependent cell adhesion or intercellular interactions (By similarity)
Mcam	0.02331	1.60	Mcam - melanoma cell adhesion molecule; Plays a role in cell adhesion, and in cohesion of the endothelial monolayer at intercellular junctions in vascular tissue. Its expression may allow melanoma cells to interact with cellular elements of the vascular system, thereby enhancing hematogeneous tumor spread. Could be an adhesion molecule active in neural crest cells during embryonic development. Acts as surface receptor that triggers tyrosine phosphorylation of FYN and PTK2/FAK1, and a transient increase in the intracellular calcium concentration (By similarity)
Col6a2	0.00963	1.59	Col6a2 - collagen, type VI, alpha 2; Collagen VI acts as a cell-binding protein
Fmod	0.01612	1.59	Fmod - fibromodulin; Affects the rate of fibrils formation. May have a primary role in collagen fibrillogenesis
Prss23	0.01218	1.59	Prss23 - protease, serine, 23
Mrvi1	0.03286	1.59	Mrvi1 - MRV integration site 1; Plays a role as NO/PRKG1-dependent regulator of IP3-induced calcium release; its phosphorylation by PRKG1 inhibits bradykinin and IP3-induced calcium release from intracellular stores. Recruits PRKG1 to the endoplasmic reticulum and may mediate the assembly of PRKG1 and ITPR1 in a macrocomplex. Involved in PRKG1 signaling cascade leading to inhibition of platelet activation and aggregation. Mediates also NO-dependent inhibition of calcium signaling in gastrointestinal smooth muscle contributing to NO-dependent relaxation
Fbn1	0.00688	1.59	Fbn1 - fibrillin 1; Fibrillins are structural components of 10-12 nm extracellular calcium-binding microfibrils, which occur either in association with elastin or in elastin-free bundles. Fibrillin-1- containing microfibrils

			provide long-term force bearing structural support. Regulates osteoblast maturation by controlling TGF-beta bioavailability and calibrating TGF-beta and BMP levels, respectively
Rgma	0.02846	1.59	Rgma - RGM domain family, member A; Member of the repulsive guidance molecule (RGM) family that performs several functions in the developing and adult nervous system. Regulates cephalic neural tube closure, inhibits neurite outgrowth and cortical neuron branching, and the formation of mature synapses. Binding to its receptor NEO1/neogenin induces activation of RHOA-ROCK1/Rho-kinase signaling pathway through UNC5B-ARHGEF12/LARG-PTK2/FAK1 cascade, leading to collapse of the neuronal growth cone and neurite outgrowth inhibition.
Etohi1	0.00745	1.58	Etohi1 - ethanol induced 1
Cpne8	0.00016	1.58	Cpne8 - copine VIII; May function in membrane trafficking. Exhibits calcium- dependent phospholipid binding properties (By similarity)
Rps4l	0.00858	1.57	Ribosomal protein S4-like
Mpp7	0.03836	1.57	Mpp7 - membrane protein, palmitoylated 7 (MAGUK p55 subfamily member 7); Acts as an important adapter that promotes epithelial cell polarity and tight junction formation via its interaction with DLG1. Involved in the assembly of protein complexes at sites of cell-cell contact (By similarity)
Zswim7	0.03580	1.57	Zswim7 - zinc finger, SWIM-type containing 7; Involved in early stages of the homologous recombination repair (HRR) pathway of double-stranded DNA breaks arising during DNA replication or induced by DNA-damaging agents (By similarity)
Tuba1b	0.00073	1.57	Tuba1b - tubulin, alpha 1B; Tubulin is the major constituent of microtubules. It binds two moles of GTP, one at an exchangeable site on the beta chain and one at a non-exchangeable site on the alpha chain (By similarity)
Sh3pxd2a	0.01860	1.57	Sh3pxd2a - SH3 and PX domains 2A; Adapter protein involved in invadopodia and podosome formation, extracellular matrix degradation and invasiveness of some cancer cells. Binds matrix metalloproteinases (ADAMs), NADPH oxidases (NOXs) and phosphoinositides. Acts as an organizer protein that allows NOX1- or NOX3-dependent reactive oxygen species (ROS) generation and ROS localization. In association with ADAM12, mediates the neurotoxic effect of beta-amyloid peptide (By similarity)
Col1a2	0.01209	1.56	Col1a2 - collagen, type I, alpha 2; Type I collagen is a member of group I collagen

			(fibrillar forming collagen)
Abi3bp	0.00305	1.56	Abi3bp - ABI gene family, member 3 (NESH) binding protein
Omd	0.01566	1.56	Omd - osteomodulin; May be implicated in biomineralization processes. Has a function in binding of osteoblasts via the alpha(V)beta(3)-integrin (By similarity)
Rbp1	0.02841	1.56	Rbp1 - retinol binding protein 1, cellular; Intracellular transport of retinol
Fscn1	0.00845	1.56	Fscn1 - fascin homolog 1, actin bundling protein (Strongylocentrotus purpuratus); Organizes filamentous actin into bundles with a minimum of 4.1:1 actin/fascin ratio. Plays a role in the organization of actin filament bundles and the formation of microspikes, membrane ruffles, and stress fibers. Important for the formation of a diverse set of cell protrusions, such as filopodia, and for cell motility and migration (By similarity)
Fdps	0.02259	1.55	Fdps - farnesyl diphosphate synthetase; Key enzyme in isoprenoid biosynthesis which catalyzes the formation of farnesyl diphosphate (FPP), a precursor for several classes of essential metabolites including sterols, dolichols, carotenoids, and ubiquinones. FPP also serves as substrate for protein farnesylation and geranylgeranylation. Catalyzes the sequential condensation of isopentenyl pyrophosphate with the allylic pyrophosphates, dimethylallyl pyrophosphate, and then with the resultant geranylpyrophosphate to the ultimate product farnesyl pyrophosphate (By similarity)
Fbln7	0.00336	1.55	Fbln7 - fibulin 7; An adhesion molecule that interacts with extracellular matrix molecules in developing teeth and may play important roles in differentiation and maintenance of odontoblasts as well as in dentin formation
Cyp1b1	0.00273	1.55	Cyp1b1 - cytochrome P450, family 1, subfamily b, polypeptide 1; Cytochromes P450 are a group of heme-thiolate monooxygenases. In liver microsomes, this enzyme is involved in an NADPH-dependent electron transport pathway. It oxidizes a variety of structurally unrelated compounds, including steroids, fatty acids, and xenobiotics
Col1a1	0.02833	1.55	Col1a1 - collagen, type I, alpha 1; Type I collagen is a member of group I collagen (fibrillar forming collagen)
Emp2	0.00233	1.55	Emp2 - epithelial membrane protein 2
Gm21685	0.00004	1.55	Lysal1 - Ectonucleoside triphosphate diphosphohydrolase 4 ; Hydrolyzes preferentially nucleoside 5'-diphosphates, nucleoside 5'-triphosphates are hydrolyzed only

			to a minor extent (By similarity)
Zfp524	0.00639	1.55	Zfp524 - zinc finger protein 524; May be involved in transcriptional regulation
Sparcl1	0.00720	1.55	Sparcl1 - SPARC-like 1
Aspn	0.01046	1.54	Aspn - asporin; Binds calcium and plays a role in osteoblast-driven collagen biomineralization activity (By similarity). Critical regulator of TGF-beta in articular cartilage and plays an essential role in cartilage homeostasis and osteoarthritis (OA) pathogenesis. Negatively regulates chondrogenesis in the articular cartilage by blocking the TGF-beta/receptor interaction on the cell surface and inhibiting the canonical TGF-beta/Smad signal
Ceacam1	0.00122	1.54	Ceacam1 - carcinoembryonic antigen-related cell adhesion molecule 1; Unknown. In case of murine coronavirus (MHV) infection, serves as receptor for MHV S1 spike glycoprotein
Dusp23	0.00449	1.54	Dusp23 - dual specificity phosphatase 23; Protein phosphatase that mediates dephosphorylation of proteins phosphorylated on Tyr and Ser/Thr residues. In vitro, it can dephosphorylate p44-ERK1 (MAPK3) but not p54 SAPK-beta (MAPK10) in vitro. Able to enhance activation of JNK and p38 (MAPK14)
Sncg	0.02908	1.54	Sncg - synuclein, gamma; Plays a role in neurofilament network integrity. May be involved in modulating axonal architecture during development and in the adult. In vitro, increases the susceptibility of neurofilament-H to calcium-dependent proteases. May also function in modulating the keratin network in skin. Activates the MAPK and Elk-1 signal transduction pathway (By similarity)
Dhrs3	0.01642	1.53	Dhrs3 - dehydrogenase/reductase (SDR family) member 3; Catalyzes the reduction of all-trans-retinal to all- trans-retinol in the presence of NADPH (By similarity)
Mgp	0.03721	1.53	Mgp - matrix Gla protein; Associates with the organic matrix of bone and cartilage. Thought to act as an inhibitor of bone formation
Arhgdib	0.00235	1.53	Arhgdib - Rho, GDP dissociation inhibitor (GDI) beta; Regulates the GDP/GTP exchange reaction of the Rho proteins by inhibiting the dissociation of GDP from them, and the subsequent binding of GTP to them
Slc18b1	0.00109	1.52	Slc18b1 - RIKEN cDNA 1110021L09 gene
Des	0.01877	1.52	Des - desmin; Desmin are class-III intermediate filaments found in muscle cells. In adult striated muscle they form a fibrous network connecting myofibrils to each other and to the plasma membrane from the periphery of the Z-line

			structures
Tmem38b	0.01834	1.52	Tmem38b - transmembrane protein 38B; Monovalent cation channel required for maintenance of rapid intracellular calcium release. May act as a potassium counter-ion channel that functions in synchronization with calcium release from intracellular stores
Hacd1	0.01229	1.52	Ptpla - protein tyrosine phosphatase-like (proline instead of catalytic arginine), member a; Responsible for the dehydration step in very long-chain fatty acid (VLCFA) synthesis (By similarity)
Cldn5	0.01464	1.51	Cldn5 - claudin 5; Plays a major role in tight junction-specific obliteration of the intercellular space, through calcium- independent cell-adhesion activity (By similarity)
Lsp1	0.02356	1.51	Lsp1 - lymphocyte specific 1
Ptpn4	0.00796	1.51	Ptpn4 - protein tyrosine phosphatase, non-receptor type 4; May act at junctions between the membrane and the cytoskeleton (By similarity)
2300009A05Rik	0.00035	1.51	ENSMUSG00000032403 - RIKEN cDNA 2300009A05 gene
Prrg3	0.03046	1.51	Prrg3 - proline rich Gla (G-carboxyglutamic acid) 3 (transmembrane)
Zfp955b	0.00977	1.51	Zfp955b - zinc finger protein 955B
Tgm2	0.00716	1.50	Tgm2 - transglutaminase 2, C polypeptide; Catalyses the cross-linking of proteins and the conjugation of polyamines to proteins
